# Transmembrane Batten Disease Proteins Interact With a Shared Network of Vesicle Sorting Proteins, Impacting Their Synaptic Enrichment

**DOI:** 10.3389/fnins.2022.834780

**Published:** 2022-05-25

**Authors:** Mitchell J. Rechtzigel, Brandon L. Meyerink, Hannah Leppert, Tyler B. Johnson, Jacob T. Cain, Gavin Ferrandino, Danielle G. May, Kyle J. Roux, Jon J. Brudvig, Jill M. Weimer

**Affiliations:** ^1^Pediatrics and Rare Diseases Group, Sanford Research, Sioux Falls, SD, United States; ^2^Basic Biomedical Sciences, Sanford School of Medicine at the University of South Dakota, Vermillion, SD, United States; ^3^Department of Pediatrics, Sanford School of Medicine at the University of South Dakota, Vermillion, SD, United States

**Keywords:** lysosome, neurodegeneration, vesicle traffic, Batten disease, SNARE (soluble N-ethylmaleimide-sensitive fusion protein attachment protein receptor)

## Abstract

Batten disease is unique among lysosomal storage disorders for the early and profound manifestation in the central nervous system, but little is known regarding potential neuron-specific roles for the disease-associated proteins. We demonstrate substantial overlap in the protein interactomes of three transmembrane Batten proteins (CLN3, CLN6, and CLN8), and that their absence leads to synaptic depletion of key partners (i.e., SNAREs and tethers) and altered synaptic SNARE complexing *in vivo*, demonstrating a novel shared etiology.

## Introduction

Batten disease (also known as Neuronal Ceroid Lipofuscinoses, NCL) is a family of neurodegenerative lysosomal storage disorders caused by mutations in one of at least 13 Ceroid Lipofuscinosis Neuronal (CLN) genes (Johnson et al., 2019). Batten disease is unique among lysosomal storage disorders for the early and profound disease manifestation in the central nervous system, which has frequently been attributed (with little evidence) to a selective vulnerability of neurons to downstream consequences of lysosomal dysfunction. However, while some forms of Batten disease are caused by mutations in genes encoding for lysosomal machinery including catabolic enzymes, other forms are caused by deficiencies in extralysosomal proteins, suggesting that lysosomal dysfunction could be just one consequence of upstream primary defects that are not entirely understood.

To gain insights into upstream neuronal functions, we investigated the protein interactomes of three transmembrane Batten disease proteins: CLN3, CLN6, and CLN8. Decades of research have uncovered a range of secondary dysfunctions present in cell models of these three disorders, and recent studies in non-neuronal cell models have shed light on their participation in pathways important for lysosomal biogenesis. CLN3 influences the recycling of lysosomal cargo receptors by regulating retromer recruitment and post-Golgi trafficking (Metcalf et al., 2008; Yasa et al., 2020, 2021), and CLN6 and CLN8 cooperatively facilitate the anterograde trafficking of lysosomal cargoes (di Ronza et al., 2018; Bajaj et al., 2020). However, reliance on non-neuronal cell models has created potential knowledge gaps in our understanding of neuronal etiology. Synaptic dysfunction precedes lysosomal defects and responds differentially to therapies in some *in vivo* models of these disorders (Ahrens-Nicklas et al., 2019, 2021; Gomez-Giro, 2019), and little is known regarding how these synaptic deficits manifest.

## CLN3, CLN6, and CLN8 have Overlapping Protein Interactomes Enriched for Regulators of Vesicle Identity, Fusion, and Composition

**Figure 1:**
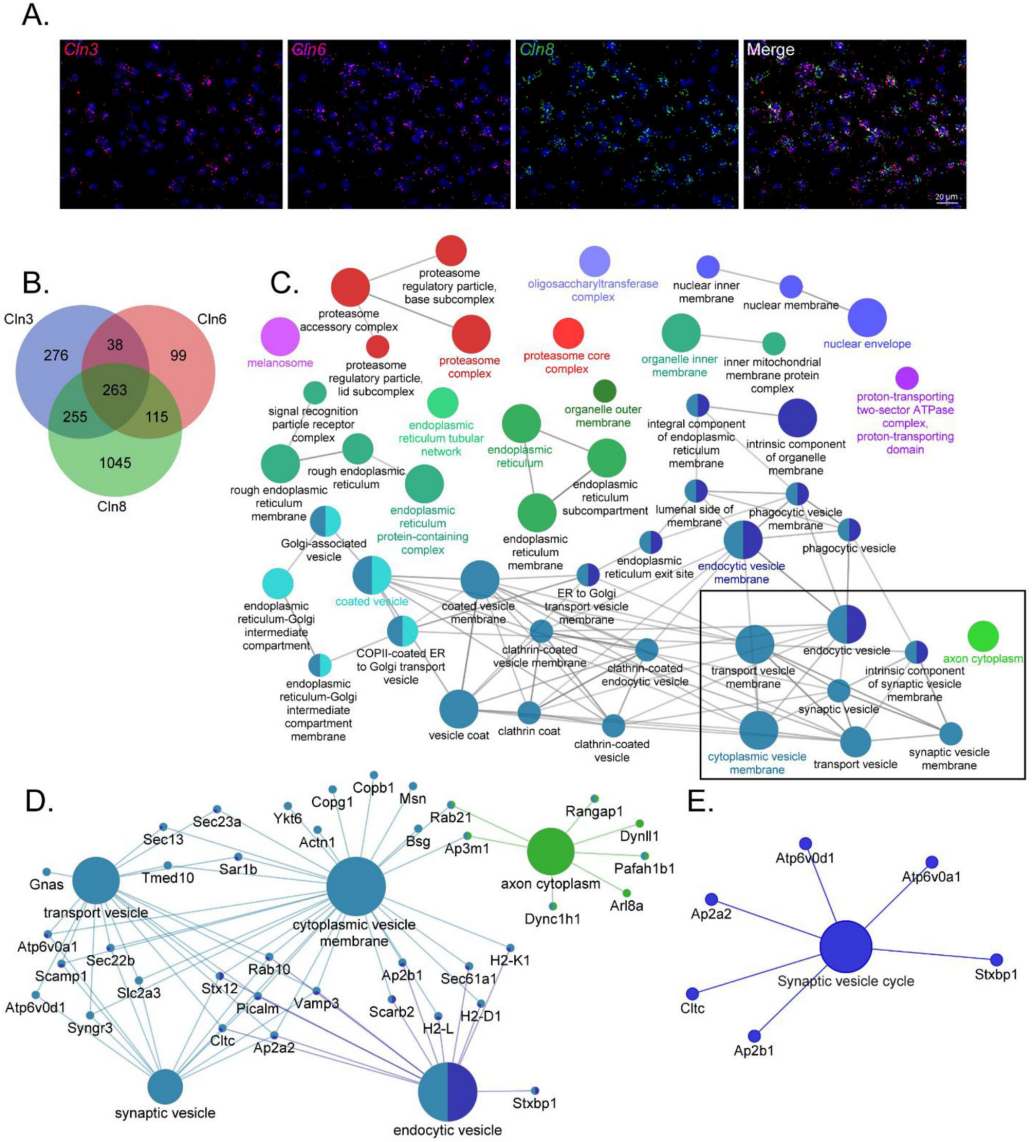
CLN3, CLN6, and CLN8-BioID share a core set of protein interactors with roles in vesicular sorting and presynaptic function. **(A)** RNAscope transcript localization indicates cellular co-occurrence of CLN3, CLN6, and CLN8 in the P21 mouse cortex. **(B)** Venn diagram depicting the number of significantly enriched proteins for each bait protein. **(C)** ClueGO network of significantly enriched Gene Ontology Consortium (GO) cellular compartment terms of common BioID interactors, with a box indicating categories involved in vesicle trafficking and sorting. **(D)** Shared protein interactors are associated with key cellular compartment terms. **(E)** KEGG Functional terms with corresponding proteins from common BioID interactors.

**Figure 2:**
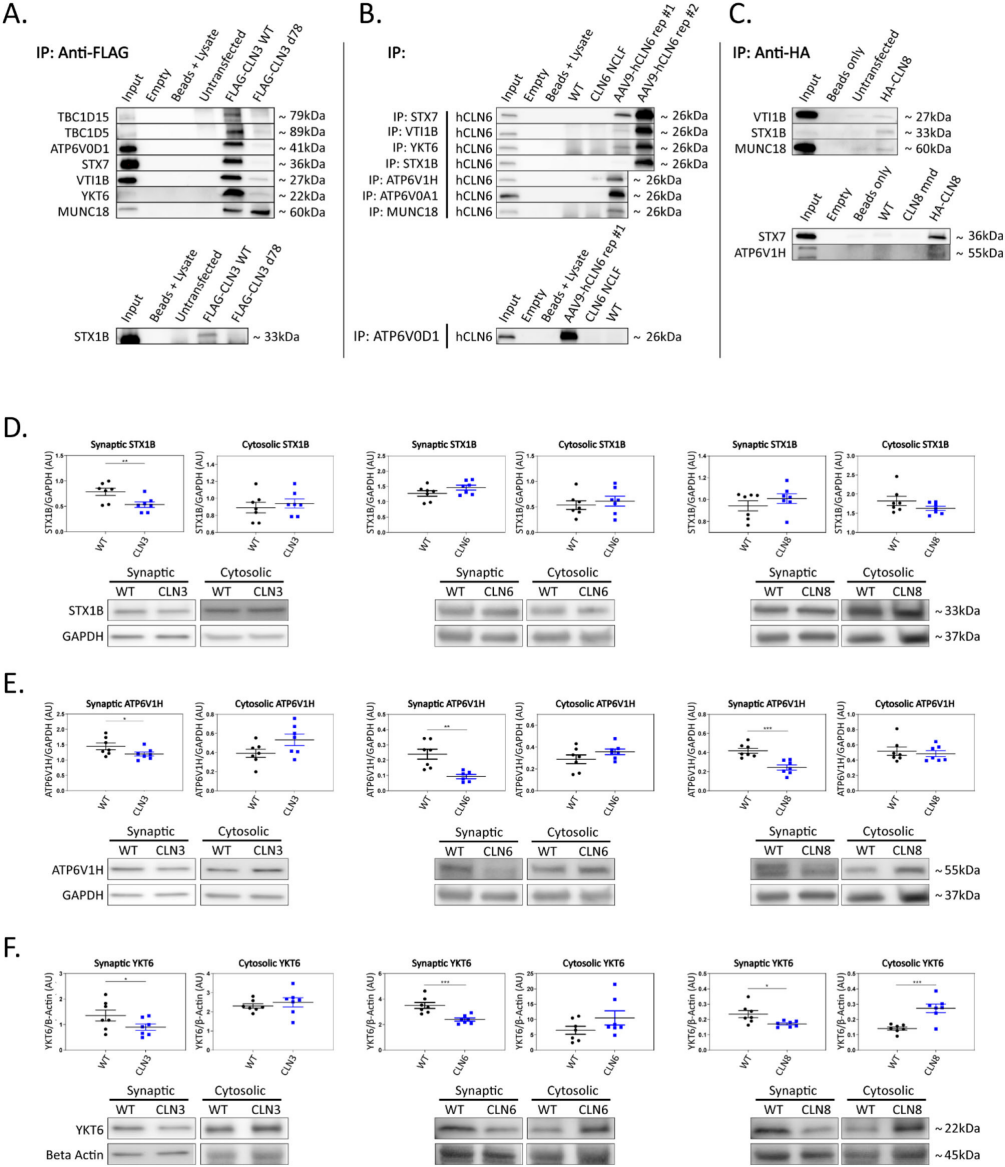
Shared interactors are depleted in cortical synapses. **(A)** Western blot analyses confirm stable CLN3 interaction with TBC1D15, TBC1D5, ATP6V0D1, STX7, VTI1B, YKT6, MUNC18, and STX1B, **(B)** stable hCLN6 interaction with STX7, VTI1B, YKT6, STX1B, ATP6V1H, ATP6V0A1, MUNC18, and ATP6V0D1, **(C)** and stable CLN8 interaction with STX1B, STX7, and ATP6V1H. Input ~ 3% total protein loaded on IPs. Due to the lack of commercially available antibodies, tagged CLN3 and CLN8 expression plasmids were used for immunoprecipitation experiments. **(D)** Analysis of synaptic and cytosolic brain fractions show synaptic depletion of STX1B in *Cln3*^Δ7/8^ samples **(E)**, and synaptic depletion of ATP6V1H in *Cln3*^Δ7/8^, *Cln6*^*nclf*^, and *Cln8*^*mnd*^ samples. **(F)** YKT6 was significantly depleted in synaptic fractions of *Cln3*^Δ7/8^, *Cln6*^*nclf*^, and *Cln8*^*mnd*^ mutant lines. Also noted was cytosolic accumulation in *Cln8*^*mnd*^ samples. AAV9-hCLN6 representatives showed varied transgene expression through interactions maintained. Multiple targets were probed on the same membrane following antibody stripping. Outliers identified by ROUT analyses, Q = 5%. One-tailed *t*-test (synaptic), two-tailed *t*-test (cytosolic), *p < 0.05, **p < 0.01, ***p < 0.001, *n* = 7 mice, mean ± SEM.

Given the phenotypic, pathological, and cell biological similarities between CLN3, CLN6, and CLN8 Batten disease, and their overlapping expression patterns in cortical neurons (Figure 1A), we hypothesized that the three proteins may participate in shared pathways essential for neuronal function. We performed proximity-dependent biotin identification (BioID) screens for each of the three proteins in neuroblastoma cells to elucidate their protein interactomes in a neuron-like cell line (Supplementary Figure 1). This identified a large number of enriched proteins in CLN3 (832), CLN6 (515), and CLN8 (1678) samples. Remarkably, there was a substantial degree of overlap between the three groups, with a core set of 263 shared proteins (Figure 1B). Within this shared interactome, pathway analysis demonstrated enrichment for proteins involved in vesicle identity, fusion, and composition [i.e., soluble N-ethylmaleimide-sensitive factor attachment protein receptor (SNAREs), tethers, and adapter proteins; Figures 1C,D], as well as proteins essential for synaptic vesicle function (Figure 1E). We confirmed a number of these interactions with co-immunoprecipitation using neuroblastoma cells and mouse cortical lysates, validating the specificity of our BioID screen (Figures 2A–C; Supplementary Table 1). While these shared interactions may be due to interactions between CLN3, CLN6, and CLN8, there was no enrichment between these proteins based on our BioID screens.

## CLN3, CLN6, and CLN8 Interactors are Depleted in Cortical Synaptosomes, Leading to Defects in the Synaptic SNARE Association State

To investigate the functional relevance of the shared interactions captured in our screen, we examined the subcellular localization of a subset of proteins with roles in vesicle fusion and presynaptic function. Fresh brain cortices were micro-dissected from postnatal day 30 wild type, *Cln3*^Δ*ex*7/8^, *Cln6*^*nclf*^, and *Cln8*^*mnd*^ mice [loss of function models for Batten disease (Bronson et al., 1993; Cotman et al., 2002; Morgan et al., 2013)], followed by synaptic and cytosolic isolation by density separation (Supplementary Figure 2F). This revealed striking defects in synaptic composition across all three genotypes (Figures 2D–F; Supplementary Figures 2A–E; Supplementary Table 2). Some targets displayed decreased synaptic abundance in all three genotypes, including ATPase H+ Transporting V1 Subunit H [ATP6V1H, a component of the vacuolar ATPase complex that acidifies lysosomes and synaptic vesicles, facilitating neurotransmitter loading and SNARE-mediated exoctytosis (Hiesinger et al., 2005; Poëa-Guyon et al., 2013; Bodzeta et al., 2017); Figure 2E], and Synaptobrevin homolog YKT6 [YKT6, a neuron-enriched SNARE protein localized to a novel vesicular compartment (Hasegawa et al., 2003), Figure 2F], while other targets displayed genotype-specific patterns of depletion, including Syntaxin 7 [STX7, an endosomal Q-SNARE that defines a specialized synaptic vesicle pool in hippocampal neurons (Mori et al., 2021), Supplementary Figures 2A,E] and ATPase H+ Transporting V0 subunit D1 (ATP6V0D1, another component of the vacuolar ATPase that acidifies lysosomes and synaptic vesicles Supplementary Figures 2C,E) in *Cln3*^Δ*ex*7/8^, and mammalian uncoordinated-18 [MUNC18, an essential component of the synaptic vesicle SNARE complex (Verhage et al., 2000), Supplementary Figures 2D,E] in *Cln6*^*nclf*^. In many cases, synaptic depletion was accompanied by cytosolic enrichment, suggesting defects in soma to synaptic terminal sorting or trafficking.

**Figure 3:**
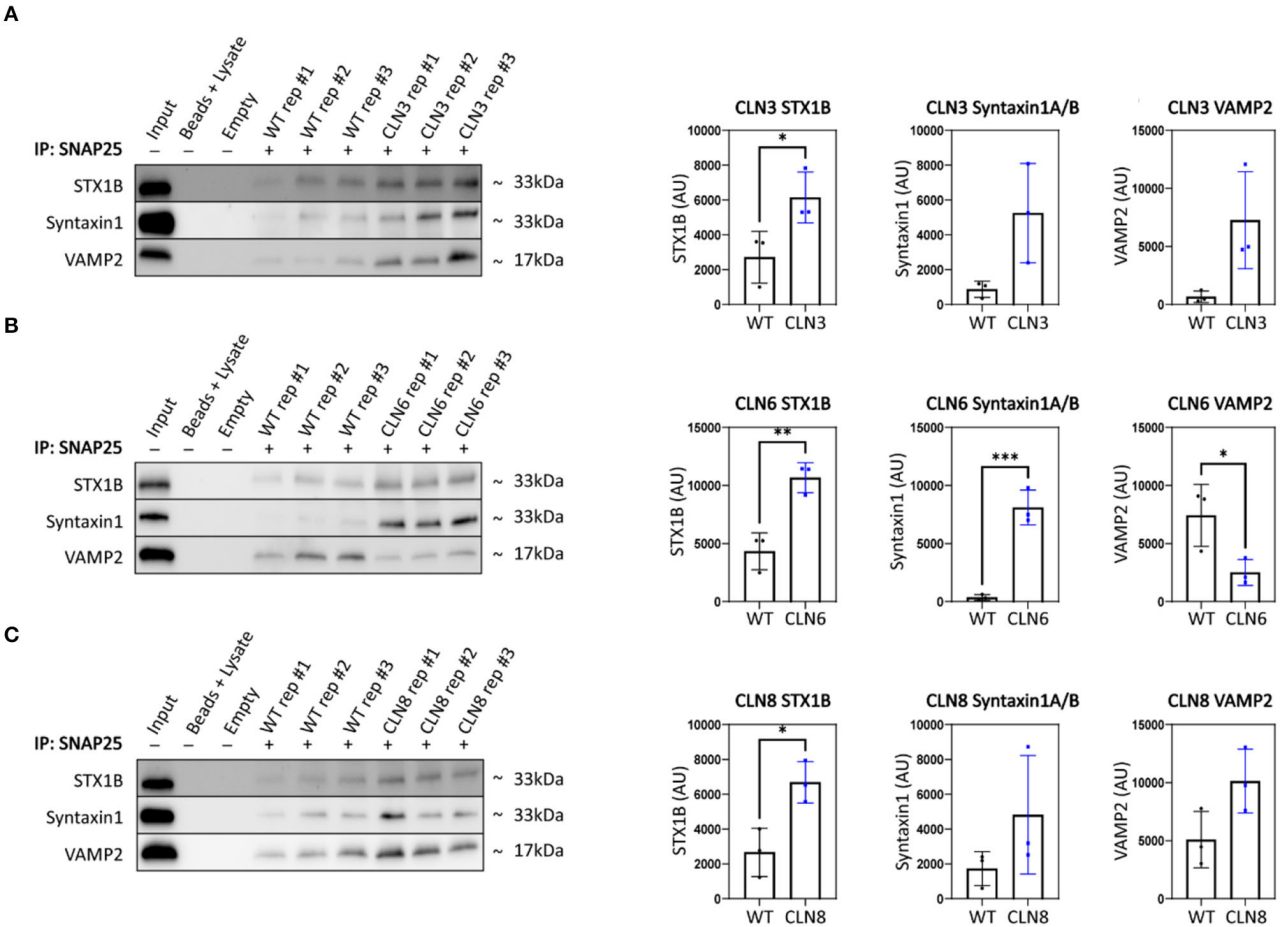
SNAP25 coimmunoprecipitations demonstrate synaptic SNARE dysfunction in *Cln3*^Δ7/8^, *Cln6*^*nclf*^, and *Cln8*^*mnd*^ mice. To analyze core SNARE complex formation, P30 mutant brain lysate was immunoprecipitated with anti-SNAP25 antibody and probed for antibodies directed against STX1B, STX1 (recognizes A and B isoforms), and VAMP. **(A)** Analyses reveal a significant increase in SNAP25-bound STX1B in CLN3, **(B)** a significant increase in SNAP25-bound STX1B and STX1, and a significant decrease in VAMP2 in Cln6 mutants, **(C)** an increased binding of SNAP25 and STX1B in CLN8 mutants. Input ~ 3% total protein loaded on IPs. Two-tailed *t*-test, *p < 0.05, **p < 0.01, ***p < 0.001, *n* = 3, mean ± SEM.

Synaptic terminal composition is tightly regulated and altered stoichiometry of key partners could lead to impaired function. To investigate this possibility, we examined the synaptic vesicle SNARE association state in our disease models. When functioning properly, synaptic vesicles dock and fuse *via* engagement between vesicular SNAREs on synaptic vesicles (e.g., Synaptobrevin 2, VAMP2) and target snares on the plasma membrane (e.g., Syntaxins 1A and/or 1B, STX1A/STX1B; Synaptosome associated protein 25, SNAP-25). Following neurotransmitter exocytosis, the vesicle fusing ATPase (NSF) dissociates the SNARE complex allowing for vesicle recycling and further rounds of loading and release. Thus, physical associations between these SNARE components can be used to monitor the extent of docking and fusion of synaptic vesicles (Sharma et al., 2011). We performed SNAP25 coimmunoprecipitations on mouse cortices and quantified levels of bound STX1 and VAMP2, finding prominent aberrations in all three disease models (Figures 3A–C). Levels of bound STX1 were significantly increased by at least 2-fold in *Cln3*^Δ*ex*7/8^, *Cln6*^*nclf*^, and *Cln8*^*mnd*^. VAMP2 trended toward greater levels in *Cln3*^Δ*ex*7/8^ and *Cln8*^*mnd*^ but was bound at significantly lower levels in *Cln6*^*nclf*^ cortices. These results suggest synaptic SNARE complex formation or dissociation may be altered in all three of these Batten disease models as a consequence of disrupted abundance in various cellular compartments.

Collectively, our results demonstrate a new etiology shared across three neurodegenerative lysosomal storage disorders and demonstrate novel neuron-specific roles for CLN3, CLN6, and CLN8 in the regulation of synaptic composition. The fact that key regulators of vesicle targeting (i.e., SNAREs and tethers) are dysregulated similarly to lysosomal proteins (e.g., vacuolar ATPase subunits), provides an intriguing link between seemingly disparate dysfunctions in synapses and lysosomes. Further work will be required to define the molecular-level causes of these defects and the relative contributions of the various synaptically depleted interacting proteins. This work suggests that these transmembrane Batten proteins interact with a diverse repertoire of proteins important for cellular trafficking and vesicular sorting and supports a multi-faceted disease etiology wherein lysosomal dysfunction is only one salient consequence.

## Data Availability Statement

The original contributions presented in the study are included in the article/Supplementary Material, further inquiries can be directed to the corresponding author/s.

## Ethics Statement

The animal study was reviewed and approved by Sanford Research IACUC.

## Author Contributions

The project was conceived by JB, TJ, JC, and JW. JB, MR, and BM designed the experiments. Experiments were performed by MR, BM, DM, KR, HL, and GF. The manuscript was prepared by MR, BM, and JB. JB, JW, MR, BM, TJ, JC, HL, GF, DM, and KR reviewed and edited the manuscript. All authors contributed to the article and approved the submitted version.

## Funding

The authors acknowledge the support of the Sanford Research Biochemistry Core (NIGMS CoBRE P20GM106320, and the Histology and Imaging Core (NIGMS CoBRE P20GM103548). Additionally, this work was supported by the National Institutes of Health (NIH #R01NS113233 and NIH #R35GM126949) and the ForeBatten Foundation. Additional support by a fellowship to BM by the USD Neuroscience, Nanotechnology and Networks program through a grant from NSF (DGE-1633213).

## Conflict of Interest

JB and JW are employees of Amicus Therapeutics Inc. and hold equity in the company in the form of stock-based compensation. JW receives sponsored research support for the Sanford laboratory from Amicus Therapeutics. The remaining authors declare that the research was conducted in the absence of any commercial or financial relationships that could be construed as a potential conflict of interest.

## Publisher's Note

All claims expressed in this article are solely those of the authors and do not necessarily represent those of their affiliated organizations, or those of the publisher, the editors and the reviewers. Any product that may be evaluated in this article, or claim that may be made by its manufacturer, is not guaranteed or endorsed by the publisher.

## References

[B1] Ahrens-Nicklas R. C. Tecedor L. Hall A. F. Kane O. Chung R. J. Lysenko E.. (2019). Neuronal network dysfunction precedes storage and neurodegeneration in a lysosomal storage disorder. JCI Insight 4, e131961. 10.1172/jci.insight.131961 31573978 PMC6948765

[B2] Ahrens-Nicklas R. C. Tecedor L. Hall A. F. Lysenko E. Cohen A. S. Davidson B. L.. (2021). Neuronal genetic rescue normalizes brain network dynamics in a lysosomal storage disorder despite persistent storage accumulation. BiorXiv. 10.1101/2021.05.03.442437 PMC9263320 35395398

[B3] Bajaj L. Sharma J. di Ronza A. Zhang P. Eblimit A. Pal R.. (2020). A CLN6-CLN8 complex recruits lysosomal enzymes at the ER for Golgi transfer. J. Clin. Invest. 130, 4118–4132. 10.1172/JCI130955 32597833 PMC7410054

[B4] Bodzeta A. Kahms M. Klingauf J. (2017). The presynaptic v-ATPase reversibly disassembles and thereby modulates exocytosis but is not part of the fusion machinery. Cell Rep. 20, 1348–1359. 10.1016/j.celrep.2017.07.040 28793259

[B5] Bronson R. T. Lake B. D. Cook S. Taylor S. Davisson M. T. (1993). Motor neuron degeneration of mice is a model of neuronal ceroid lipofuscinosis (Batten's disease). Ann. Neurol. 33, 381–385. 10.1002/ana.410330408 7683855

[B6] Cotman S. L. Vrbanac V. Lebel L. A. Lee R. L. Johnson K. A. Donahue L. R.. (2002). Cln3(Deltaex7/8) knock-in mice with the common JNCL mutation exhibit progressive neurologic disease that begins before birth. Hum. Mol. Genet. 11, 2709–2721. 10.1093/hmg/11.22.2709 12374761

[B7] di Ronza A. Bajaj L. Sharma J. Sanagasetti D. Lotfi P. Adamski C. J.. (2018). CLN8 is an endoplasmic reticulum cargo receptor that regulates lysosome biogenesis. Nat. Cell Biol. 20, 1370–1377. 10.1038/s41556-018-0228-7 30397314 PMC6277210

[B8] Gomez-Giro G. Arias-Fuenzalida J. Jarazo J. Zeuschner D. Ali M. Possemis N.. (2019). Synapse alterations precede neuronal damage and storage pathology in a human cerebral organoid model of CLN3-juvenile neuronal ceroid lipofuscinosis. Acta Neuropathol. Commun. 7, 222. 10.1186/s40478-019-0871-7 31888773 PMC6937812

[B9] Hasegawa H. Zinsser S. Rhee Y. Vik-Mo E. O. Davanger S. Hay J. C.. (2003). Mammalian ykt6 is a neuronal SNARE targeted to a specialized compartment by its profilin-like amino terminal domain. Mol. Biol. Cell 14, 698–720. 10.1091/mbc.e02-09-0556 12589064 PMC150002

[B10] Hiesinger P. R. Fayyazuddin A. Mehta S. Q. Rosenmund T. Schulze K. L. Zhai R. G.. (2005). The v-ATPase V0 subunit a1 is required for a late step in synaptic vesicle exocytosis in Drosophila. Cell 121, 607–620. 10.1016/j.cell.2005.03.012 15907473 PMC3351201

[B11] Johnson T. B. Cain J. T. White K. A. Ramirez-Montealegre D. Pearce D. A. Weimer J. M.. (2019). Therapeutic landscape for Batten disease: current treatments and prospects. Nat. Rev. Neurol. 15, 161–178. 10.1038/s41582-019-0138-8 30783219 PMC6681450

[B12] Metcalf D. J. Calvi A. A. Seaman M. Mitchison H. M. Cutler D. F. (2008). Loss of the Batten disease gene CLN3 prevents exit from the TGN of the mannose 6-phosphate receptor. Traffic 9, 1905–1914. 10.1111/j.1600-0854.2008.00807.x 18817525

[B13] Morgan J. P. Magee H. Wong A. Nelson T. Koch B. Cooper J. D.. (2013). A murine model of variant late infantile ceroid lipofuscinosis recapitulates behavioral and pathological phenotypes of human disease. PLoS ONE 8, e78694. 10.1371/journal.pone.0078694 24223841 PMC3815212

[B14] Mori Y. Takenaka K. I. Fukazawa Y. Takamori S. (2021). The endosomal Q-SNARE, Syntaxin 7, defines a rapidly replenishing synaptic vesicle recycling pool in hippocampal neurons. Commun. Biol. 4, 981. 10.1038/s42003-021-02512-4 34408265 PMC8373932

[B15] Poëa-Guyon S. Ammar M. R. Erard M. Amar M. Moreau A. W. Fossier P.. (2013). The V-ATPase membrane domain is a sensor of granular pH that controls the exocytotic machinery. J. Cell Biol. 203, 283–298. 10.1083/jcb.201303104 24165939 PMC3812966

[B16] Sharma M. Burré J. Südhof T. C. (2011). CSPα promotes SNARE-complex assembly by chaperoning SNAP-25 during synaptic activity. Nat. Cell Biol. 13, 30–39. 10.1038/ncb2131 21151134

[B17] Verhage M. Maia A. S. Plomp J. J. Brussaard A. B. Heeroma J. H. Vermeer H.. (2000). Synaptic assembly of the brain in the absence of neurotransmitter secretion. Science 287, 864–869. 10.1126/science.287.5454.864 10657302

[B18] Yasa S. Modica G. Sauvageau E. Kaleem A. Hermey G. Lefrancois S.. (2020). CLN3 regulates endosomal function by modulating Rab7A-effector interactions. J. Cell Sci. 133, jcs234047. 10.1242/jcs.234047 32034082

[B19] Yasa S. Sauvageau E. Modica G. Lefrancois S. (2021). CLN5 and CLN3 function as a complex to regulate endolysosome function. Biochem. J. 478, 2339–2357. 10.1042/BCJ20210171 34060589

