# Supplementary material for: Transmembrane Batten Disease Proteins Interact With a Shared Network of Vesicle Sorting Proteins, Impacting Their Synaptic Enrichment
Source: Front Neurosci. 2022 May 25;16:834780. doi: 10.3389/fnins.2022.834780 (PMC9174988; doi:10.3389/fnins.2022.834780)
Supplement: Supplementary file 2 [file Data_Sheet_1.PDF]

Section 1: Uncropped blots from figure 2

CLN3 Isoform CoIP
200ug load, 50uL beads, RT Laemmli buffer elution
anti-FLAG (F1804) @ 1:50, MUNC18 @ 1:1000
Expected Size: 65kDa
06Jul2021

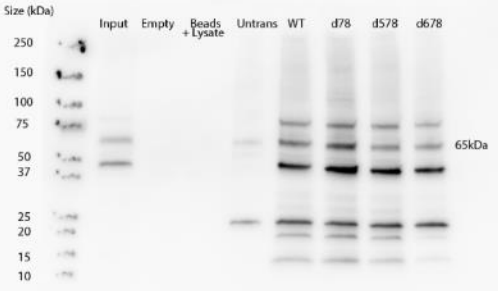

CLN3 Isoform CoIP
200ug load, 60uL beads, RT Laemmli buffer elution
anti-FLAG (F1804) @ 1:50, STX1B @ 1:1000
Expected Size: 33kDa
01Apr2021

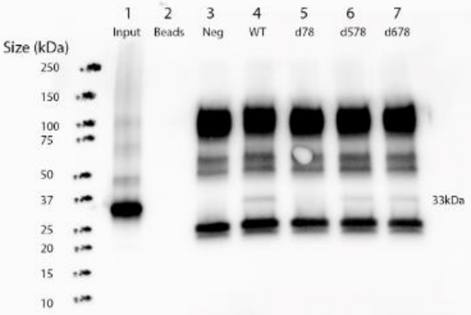

1: Input
2: Beads + Lysate
3: Untransfected N2As
4: pCAG-FLAG-CLN3 WT
5: pCAG-FLAG CLN3 d78
6: pCAG-FLAG-CLN3 d578
7: pCAG-FLAG-CLN3 d678

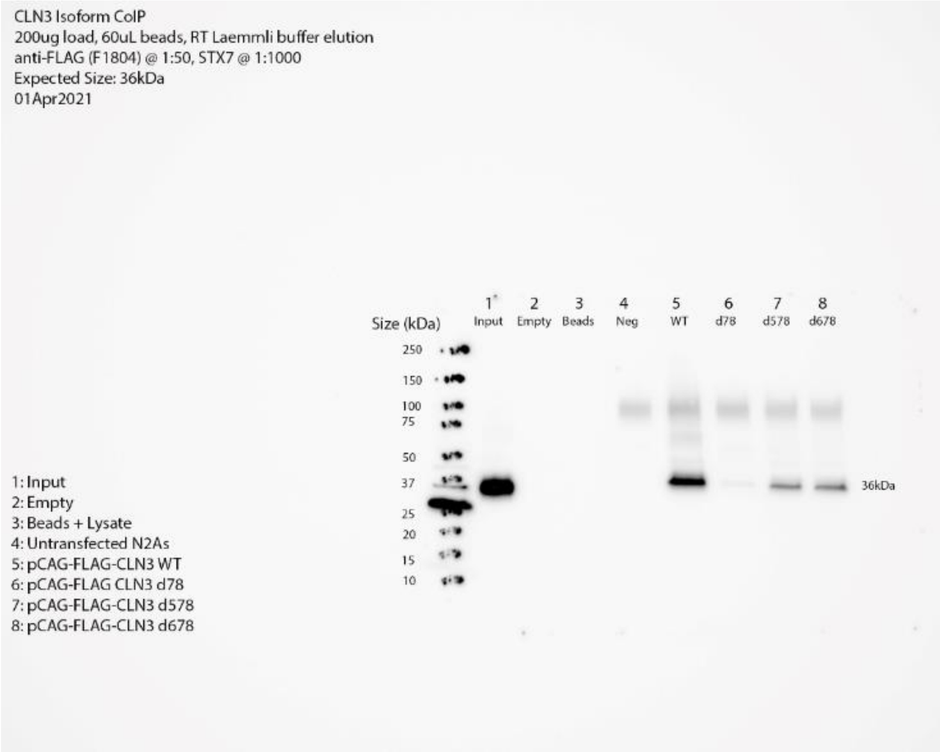
CLN3 Isoform CoIP
200ug load, 60uL beads, RT Laemmli buffer elution
anti-FLAG (F1804) @ 1:50, STX7 @ 1:1000
Expected Size: 36kDa
01Apr2021
Size (kDa)
1 Input
2 Empty
3 Beads
4 Neg
5 WT
6 d78
7 d578
8 d678
250
150
100
75
50
37
25
20
15
10
36kDa
1: Input
2: Empty
3: Beads + Lysate
4: Untransfected N2As
5: pCAG-FLAG-CLN3 WT
6: pCAG-FLAG CLN3 d78
7: pCAG-FLAG-CLN3 d578
8: pCAG-FLAG-CLN3 d678

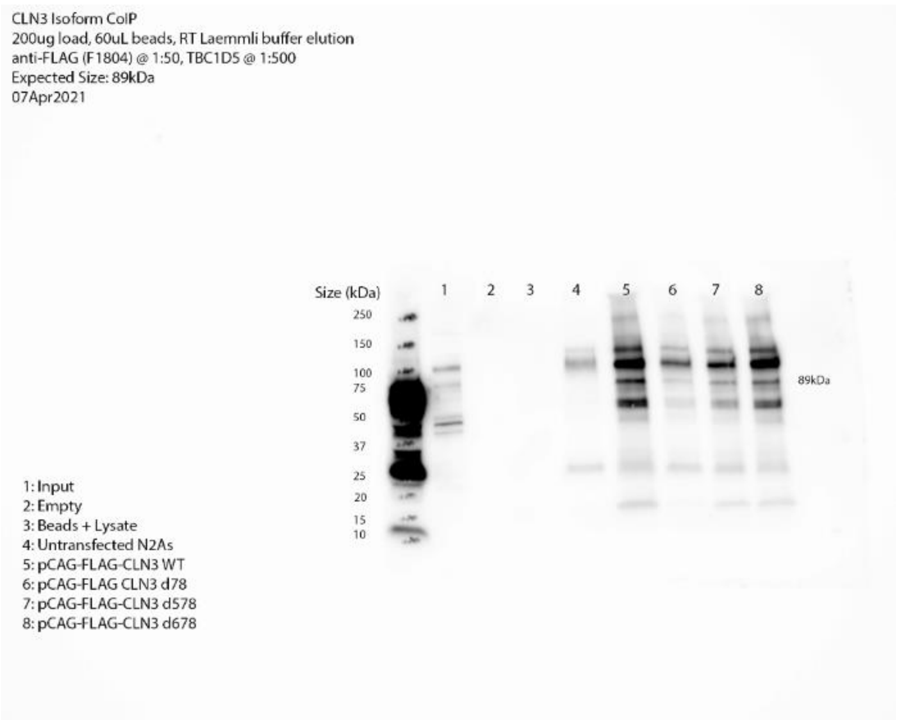
CLN3 Isoform CoIP
200ug load, 60uL beads, RT Laemmli buffer elution
anti-FLAG (F1804) @ 1:50, TBC1D5 @ 1:500
Expected Size: 89kDa
07Apr2021
Size (kDa)
1
2
3
4
5
6
7
8
250
150
100
75
50
37
25
20
15
10
89kDa
1: Input
2: Empty
3: Beads + Lysate
4: Untransfected N2As
5: pCAG-FLAG-CLN3 WT
6: pCAG-FLAG CLN3 d78
7: pCAG-FLAG-CLN3 d578
8: pCAG-FLAG-CLN3 d678

CLN3 Isoform CoIP
200ug load, 60uL beads, RT Laemmli buffer elution
anti-FLAG (F1804) @ 1:50, TBC1D15 @ 1:500
Expected Size: 79kDa
07Apr2021

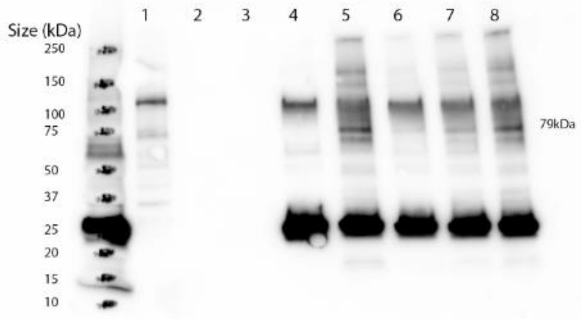

1: Input
2: Empty
3: Beads + Lysate
4: Untransfected N2As
5: pCAG-FLAG-CLN3 WT
6: pCAG-FLAG CLN3 d78
7: pCAG-FLAG-CLN3 d578
8: pCAG-FLAG-CLN3 d678

CLN3 Isoform CoIP
200ug load, 60uL beads, RT Laemmli buffer elution
anti-FLAG (F1804) @ 1:50, VTI1B @ 1:500
Expected Size: 27kDa
02Apr2021

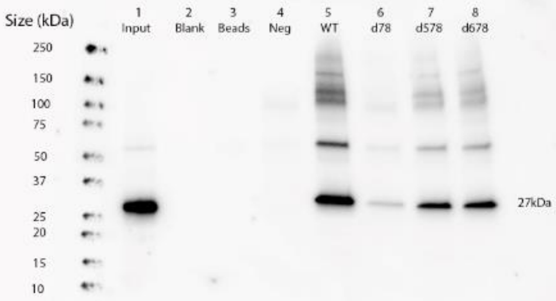

1: Input
2: Blank
3: Beads + Lysate
4: Untransfected N2As
5: pCAG-FLAG-CLN3 WT
6: pCAG-FLAG CLN3 d78
7: pCAG-FLAG-CLN3 d578
8: pCAG-FLAG-CLN3 d678

CLN3 Isoform CoIP
200ug load, 60uL beads, RT Laemmli buffer elution
anti-FLAG (F1804) @ 1:50, YKT6 @ 1:500
Expected Size: 22kDa
06Apr2021

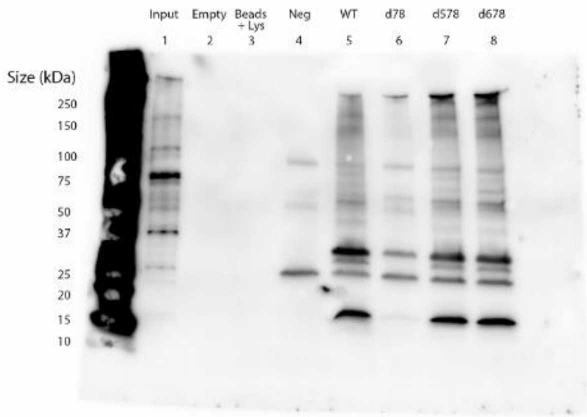

1: Input
2: Blank
3: Beads + Lysate
4: Untransfected N2As
5: pCAG-FLAG-CLN3 WT
6: pCAG-FLAG CLN3 d78
7: pCAG-FLAG-CLN3 d578
8: pCAG-FLAG-CLN3 d678

CLN6 CoIPs
200ug load, RT laemmli elution
ATP6V0a1 PD @ 1:100, CLN6 probe @ 1:1000
Expected Size: 26kDa
23Jun2021

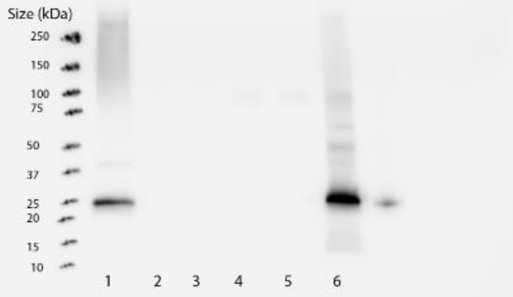

1: Input (6ug)
2: Empty
3: Beads+ lysate
4: WT
5: KO
6: CLN6 AAV

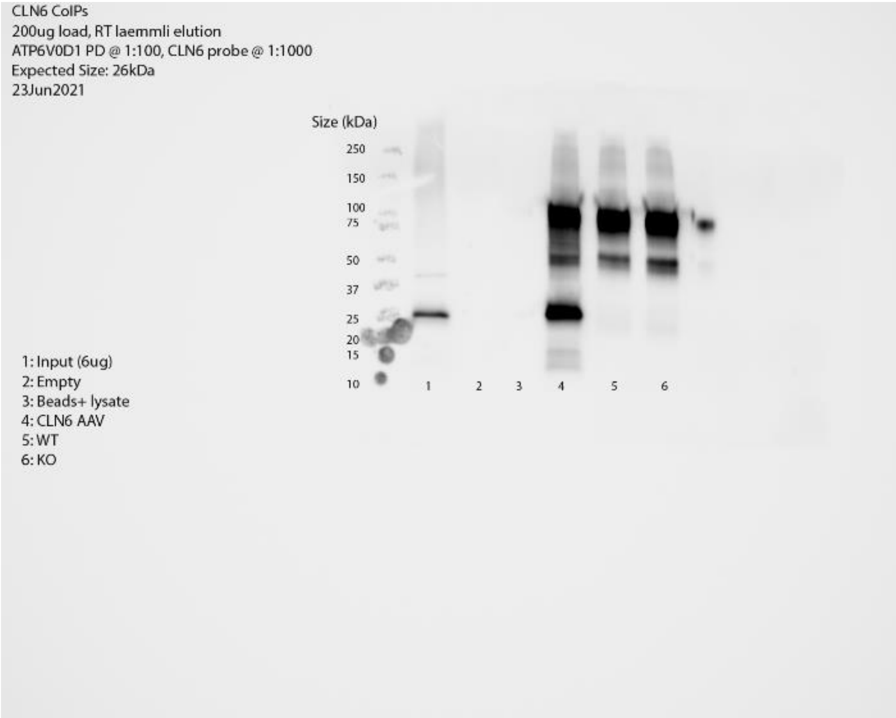
CLN6 CoIPs
200ug load, RT laemmli elution
ATP6V0D1 PD @ 1:100, CLN6 probe @ 1:1000
Expected Size: 26kDa
23Jun2021
Size (kDa)
250
150
100
75
50
37
25
20
15
10
1 2 3 4 5 6
1: Input (6ug)
2: Empty
3: Beads+ lysate
4: CLN6 AAV
5: WT
6: KO

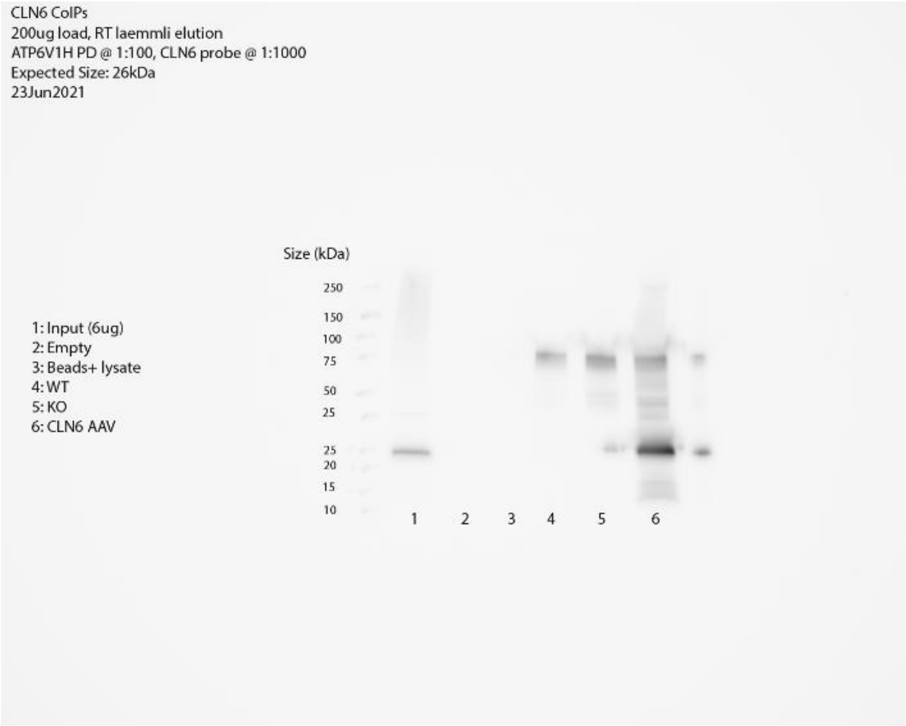
CLN6 CoIPs
200ug load, RT laemmli elution
ATP6V1H PD @ 1:100, CLN6 probe @ 1:1000
Expected Size: 26kDa
23Jun2021
Size (kDa)
250
150
100
75
50
25
25
20
15
10
1 2 3 4 5 6
1: Input (6ug)
2: Empty
3: Beads+ lysate
4: WT
5: KO
6: CLN6 AAV

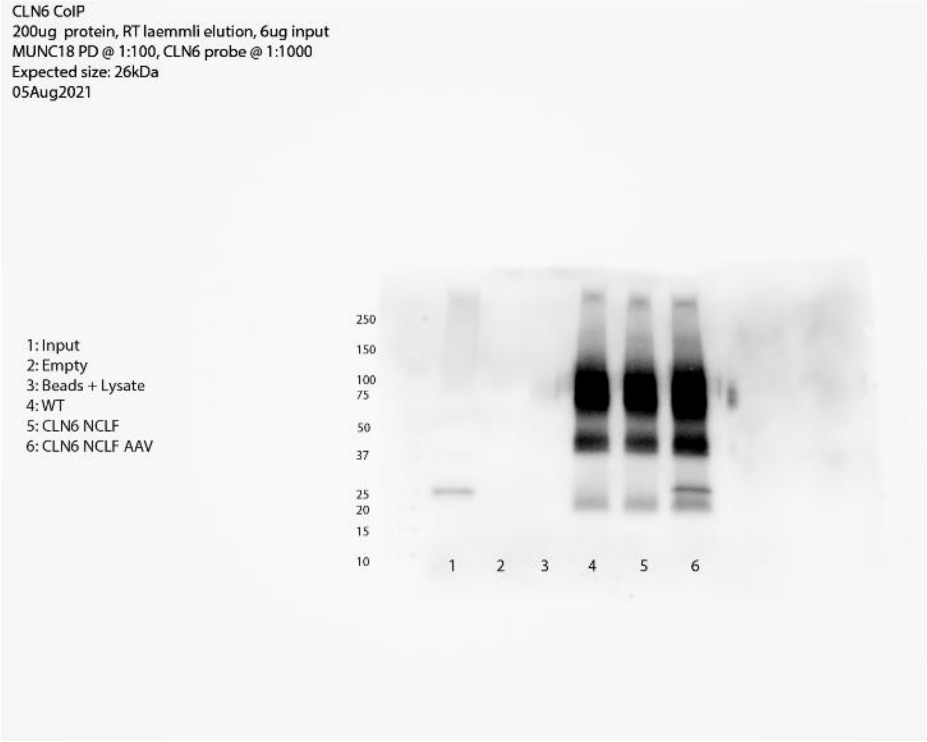
CLN6 CoIP
200ug protein, RT laemmli elution, 6ug input
MUNC18 PD @ 1:100, CLN6 probe @ 1:1000
Expected size: 26kDa
05Aug2021
1: Input
2: Empty
3: Beads + Lysate
4: WT
5: CLN6 NCLF
6: CLN6 NCLF AAV
250
150
100
75
50
37
25
20
15
10
1
2
3
4
5
6

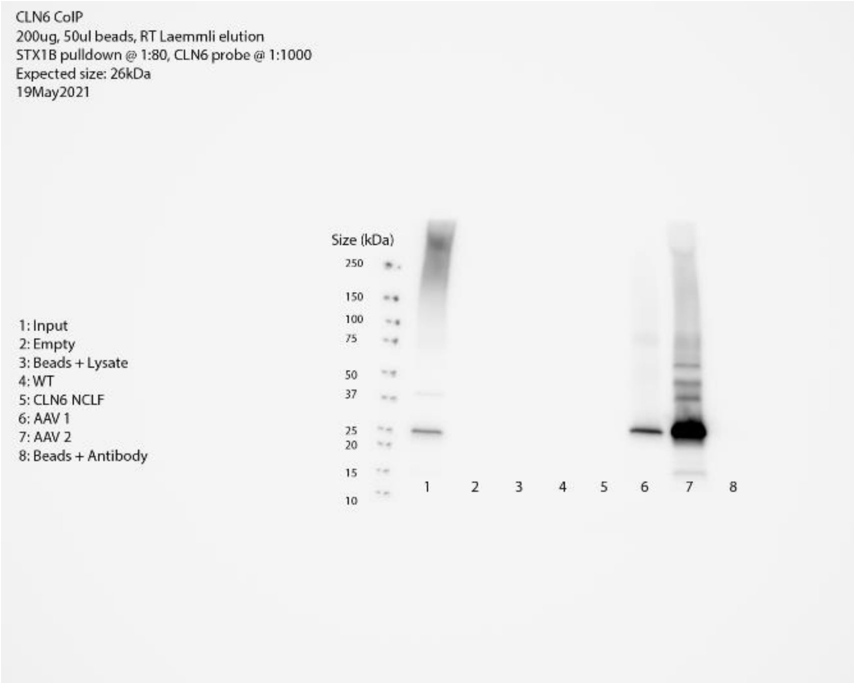
CLN6 CoIP
200ug, 50ul beads, RT Laemmli elution
STX1B pulldown @ 1:80, CLN6 probe @ 1:1000
Expected size: 26kDa
19May2021
1: Input
2: Empty
3: Beads + Lysate
4: WT
5: CLN6 NCLF
6: AAV 1
7: AAV 2
8: Beads + Antibody
Size (kDa)
250
150
100
75
50
37
25
20
15
10
1
2
3
4
5
6
7
8

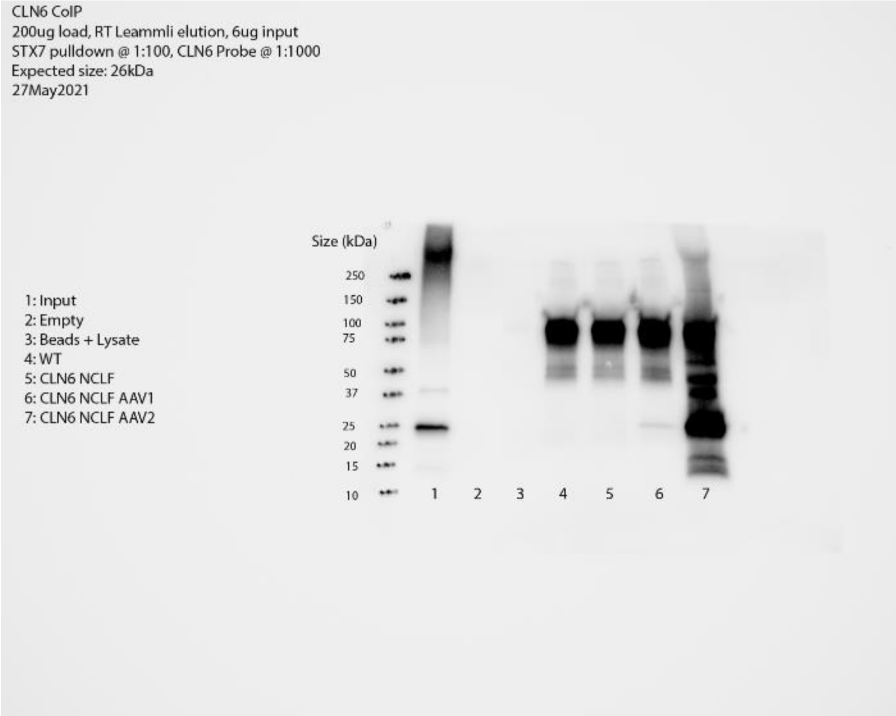
CLN6 CoIP
200ug load, RT Leammli elution, 6ug input
STX7 pulldown @ 1:100, CLN6 Probe @ 1:1000
Expected size: 26kDa
27May2021
Size (kDa)
250
150
100
75
50
37
25
20
15
10
1 2 3 4 5 6 7
1: Input
2: Empty
3: Beads + Lysate
4: WT
5: CLN6 NCLF
6: CLN6 NCLF AAV1
7: CLN6 NCLF AAV2

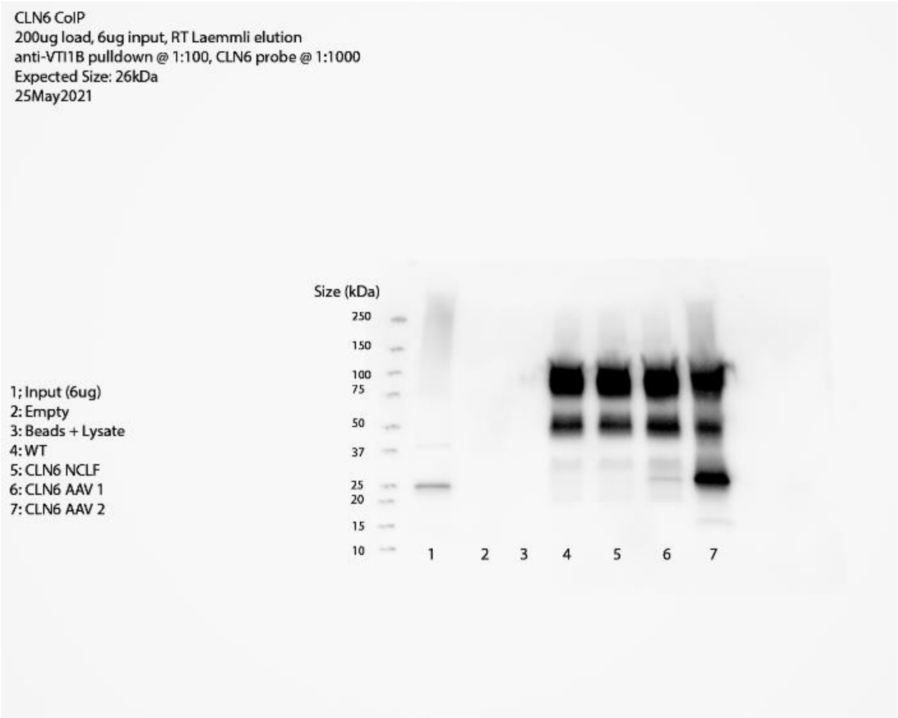
CLN6 CoIP
200ug load, 6ug input, RT Laemmli elution
anti-VTI1B pulldown @ 1:100, CLN6 probe @ 1:1000
Expected Size: 26kDa
25May2021
Size (kDa)
250
150
100
75
50
37
25
20
15
10
1 2 3 4 5 6 7
1; Input (6ug)
2: Empty
3: Beads + Lysate
4: WT
5: CLN6 NCLF
6: CLN6 AAV 1
7: CLN6 AAV 2

CLN6 CoIP
200ug load, 50uL beads, RT Laemmli elution
YKT6 pulldown @ 1:100, CLN6 probe @ 1:1000
Expected Size (22kDa)
20May2021

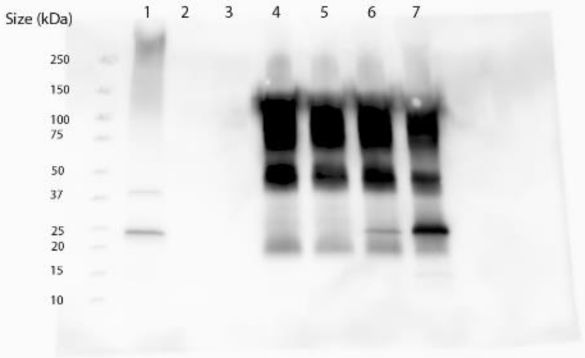

1: Input (6ug)
2: Empty
3: Beads + Lysate
4: WT
5: CLN6 NCLF
6: CLN6 AAV 1
7: CLN6 AAV 2

CLN8 CoIP (HA magnetic beads)
150ug load, RT Laemmli elution
ATP6V1h probe @ 1:1000
Expected size: 55kDa
27May2021

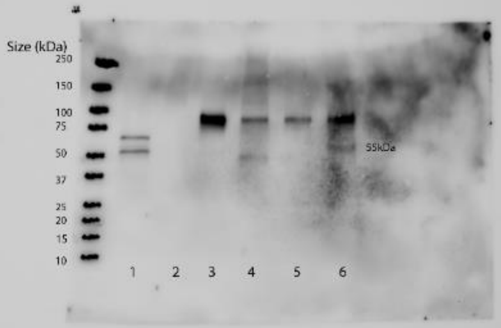

1: Input (6ug)
2: Empty
3: Beads CTRL
4: WT
5: CLN8 mnd
6: pEF1a-HA-CLN8

CLN8 CoIP (HA pulldown)
150ug load, RT laemmli elution
HA pulldown, MUNC18 probe @ 1:1000
expected size: 60kDa
13Jul2021

1: Input
2: Beads CTRL
3: Untransfectd N2As
4: pCAG-FLAG-HA-CLN8

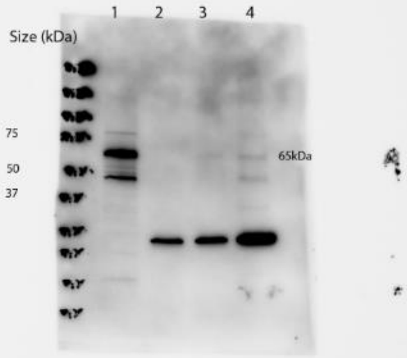

CLN8 CoIP (HA pulldown)
150ug load, RT laemmli elution, 6ug input
STX1B @ 1:1000
expectd size: 33kDa
14Jul2021

1: Input
2: Beads CTRL
3: Untransfected N2As
4: pCAG-HA-CLN8

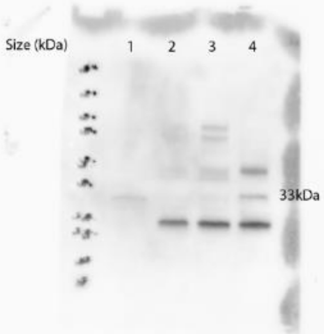

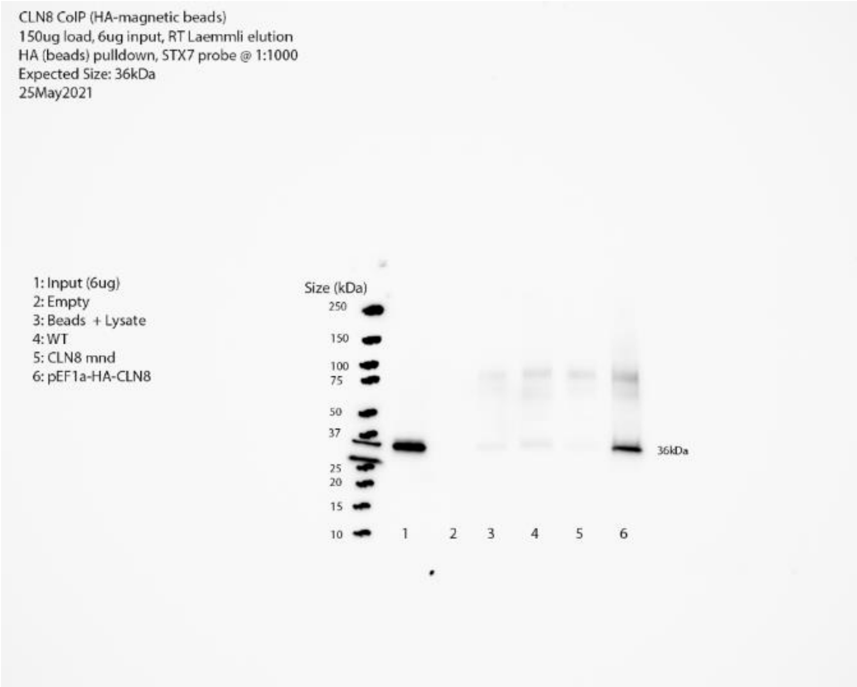
CLN8 CoIP (HA-magnetic beads)
150ug load, 6ug input, RT Laemmli elution
HA (beads) pulldown, STX7 probe @ 1:1000
Expected Size: 36kDa
25May2021
1: Input (6ug)
2: Empty
3: Beads + Lysate
4: WT
5: CLN8 mnd
6: pEF1a-HA-CLN8
Size (kDa)
250
150
100
75
50
37
25
20
15
10
1
2
3
4
5
6
36kDa

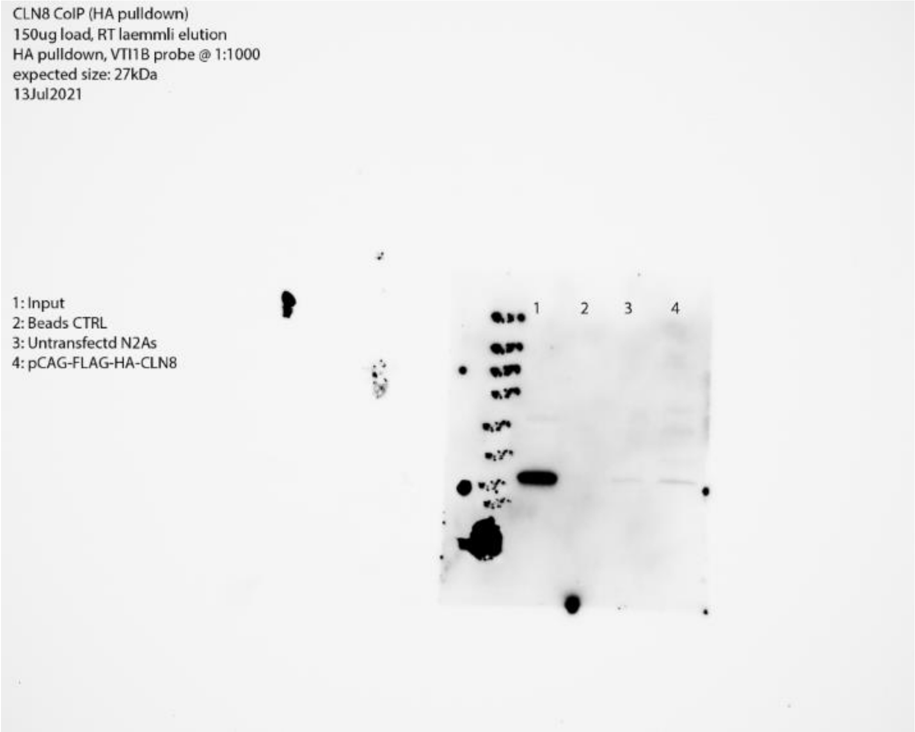
CLN8 CoIP (HA pulldown)
150ug load, RT laemmli elution
HA pulldown, VTI1B probe @ 1:1000
expected size: 27kDa
13Jul2021
1: Input
2: Beads CTRL
3: Untransfectd N2As
4: pCAG-FLAG-HA-CLN8
1
2
3
4

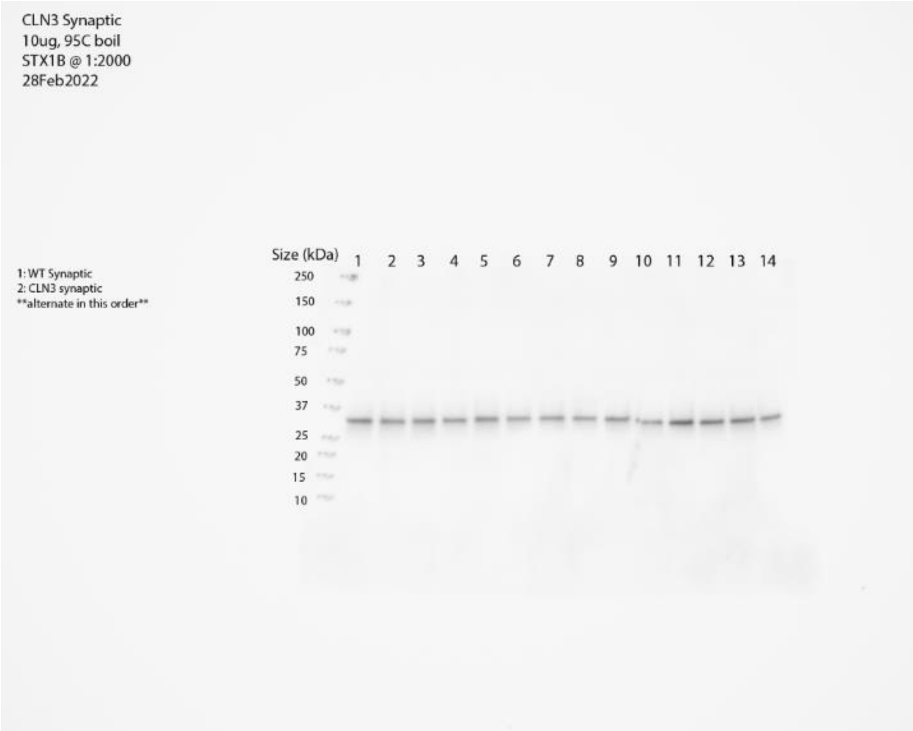
CLN3 Synaptic
10ug, 95C boil
STX1B @ 1:2000
28Feb2022
1: WT Synaptic
2: CLN3 synaptic
alternate in this order
Size (kDa)
1 2 3 4 5 6 7 8 9 10 11 12 13 14
250
150
100
75
50
37
25
20
15
10

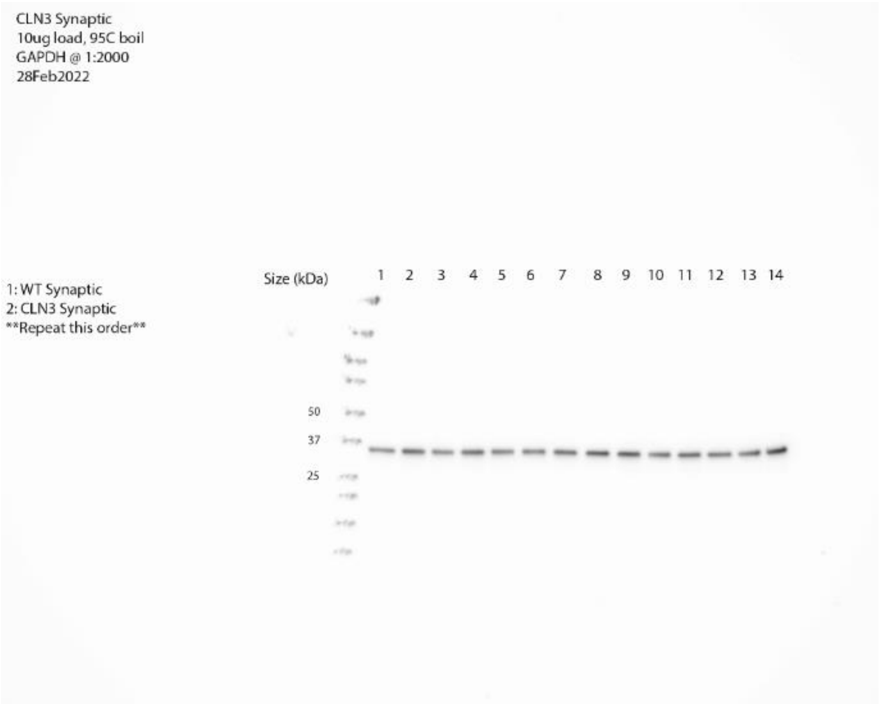
CLN3 Synaptic
10ug load, 95C boil
GAPDH @ 1:2000
28Feb2022
1: WT Synaptic
2: CLN3 Synaptic
Repeat this order
Size (kDa)
1 2 3 4 5 6 7 8 9 10 11 12 13 14
50
37
25

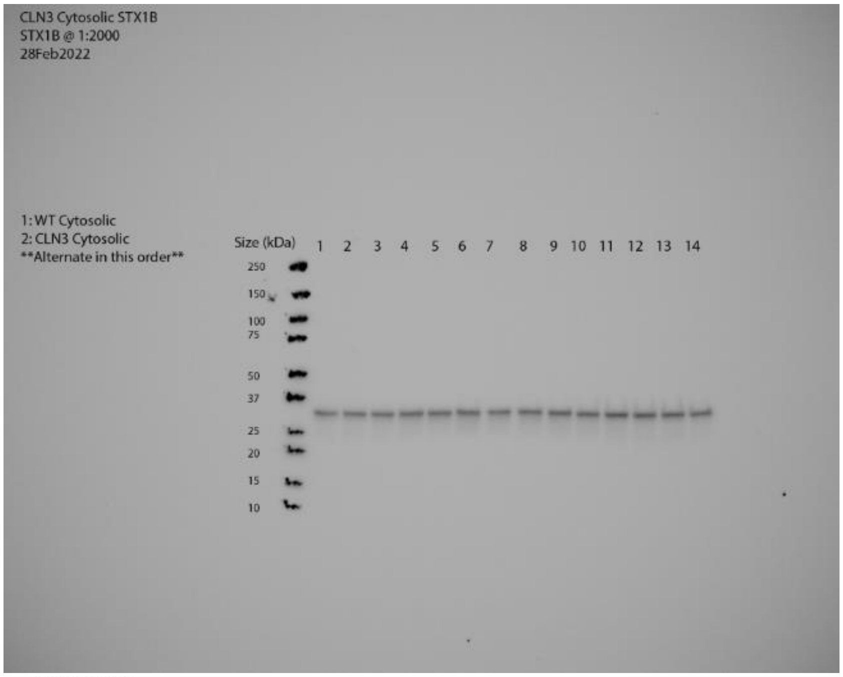
CLN3 Cytosolic STX1B
STX1B @ 1:2000
28Feb2022
1: WT Cytosolic
2: CLN3 Cytosolic
Alternate in this order
Size (kDa)
1 2 3 4 5 6 7 8 9 10 11 12 13 14
250
150
100
75
50
37
25
20
15
10

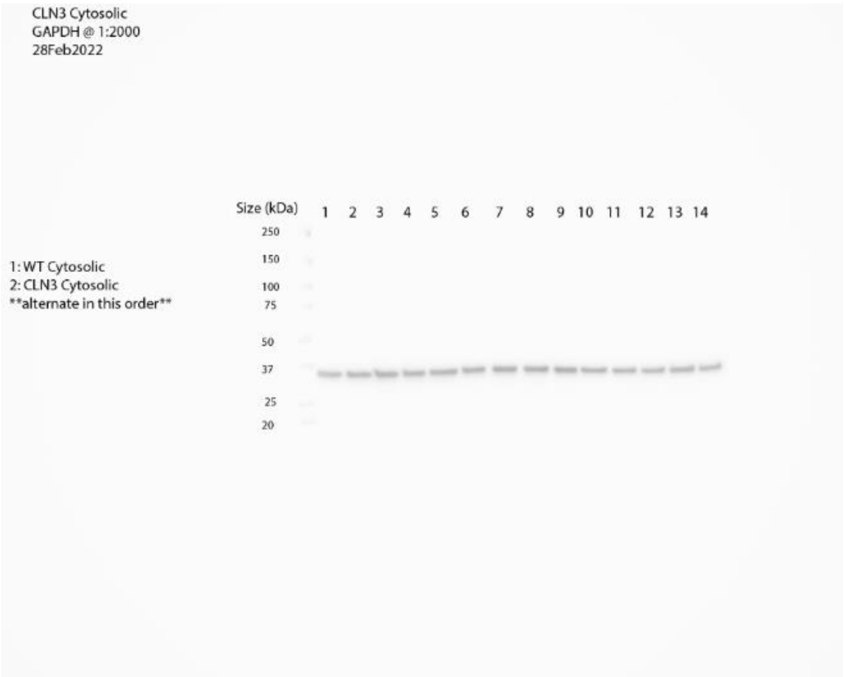
CLN3 Cytosolic
GAPDH @ 1:2000
28Feb2022
1: WT Cytosolic
2: CLN3 Cytosolic
alternate in this order
Size (kDa)
1 2 3 4 5 6 7 8 9 10 11 12 13 14
250
150
100
75
50
37
25
20

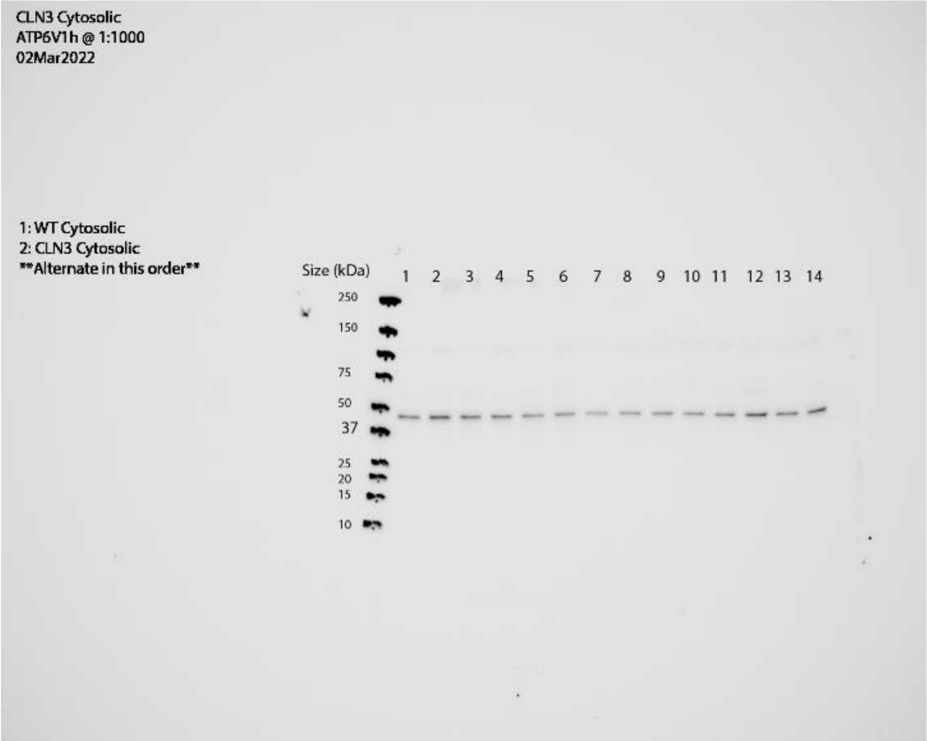
CLN3 Cytosolic
ATP6V1h @ 1:1000
02Mar2022
1: WT Cytosolic
2: CLN3 Cytosolic
Alternate in this order
Size (kDa)
1 2 3 4 5 6 7 8 9 10 11 12 13 14
250
150
75
50
37
25
20
15
10

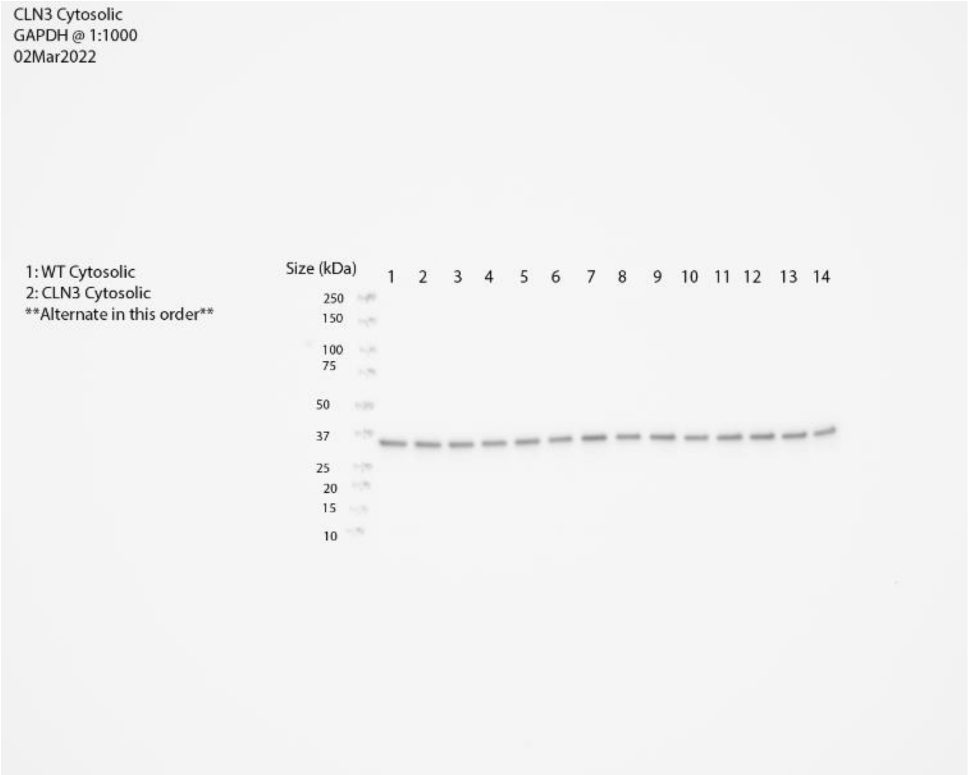
CLN3 Cytosolic
GAPDH @ 1:1000
02Mar2022
1: WT Cytosolic
2: CLN3 Cytosolic
Alternate in this order
Size (kDa)
1 2 3 4 5 6 7 8 9 10 11 12 13 14
250
150
100
75
50
37
25
20
15
10

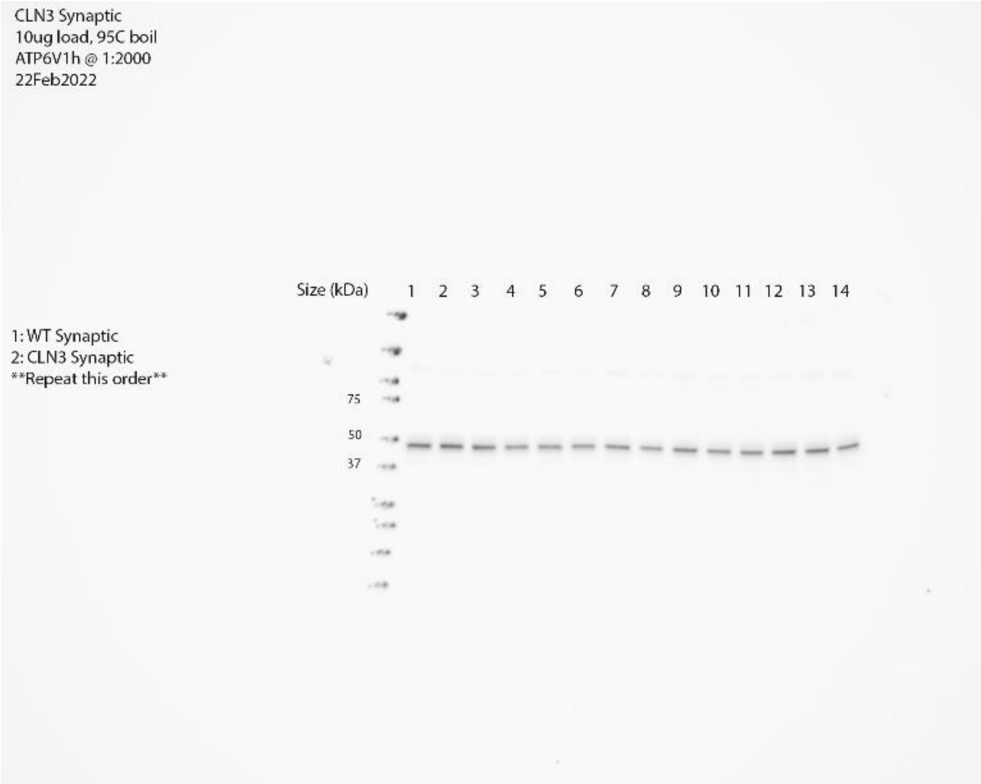

CLN3 Synaptic
10ug load, 95C boil
GAPDH @ 1:2000
22Feb2022

1: WT Synaptic
2: CLN3 Synaptic
Repeat this order

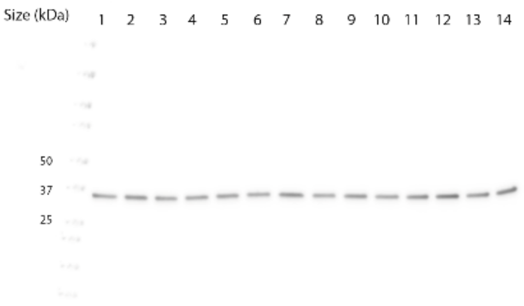

CLN3 Cytosolic
30ug, 95C boil
Beta Actin @ 1:3000
Expected Size: 45kDa
04Mar2021

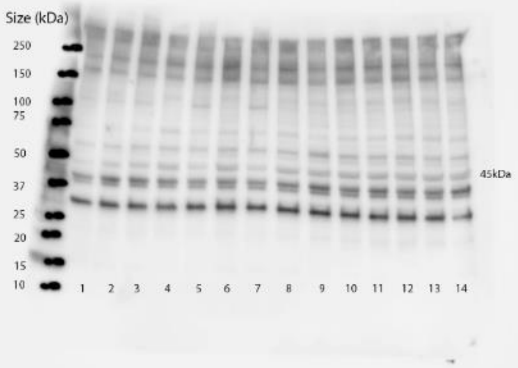

1: WT Cyto
2: WT Cyto
3: WT Cyto
4: WT Cyto
5: WT Cyto
6: CLN3 Cyto
7: WT Cyto
8: CLN3 Cyto
9: WT Cyto
10: CLN3 Cyto
11: CLN3 Cyto
12: CLN3 Cyto
13: CLN3 Cyto
14: CLN3 Cyto

CLN3 Cytosolic
30ug, 95C boil
YKT6 @ 1:1000
Expected Size: 22kDa
02Mar2021

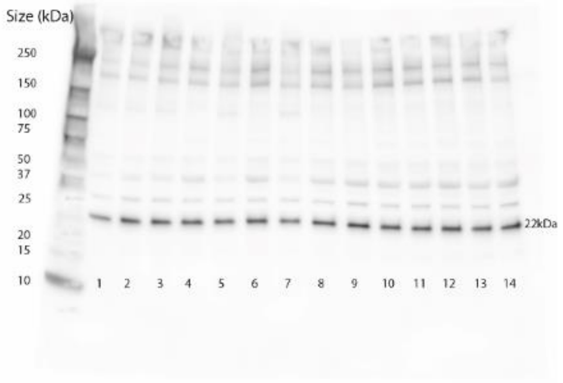

1: WT Cytosolic
2: WT Cytosolic
3: WT Cytosolic
4: WT Cytosolic
5: WT Cytosolic
6: CLN3 Cytosolic
7: WT Cytosolic
8: CLN3 Cytosolic
9: WT Cytosolic
10: CLN3 Cytosolic
11: CLN3 Cytosolic
12: CLN3 Cytosoli
13: CLN3 Cytosolic
14: CLN3 Cytosolic

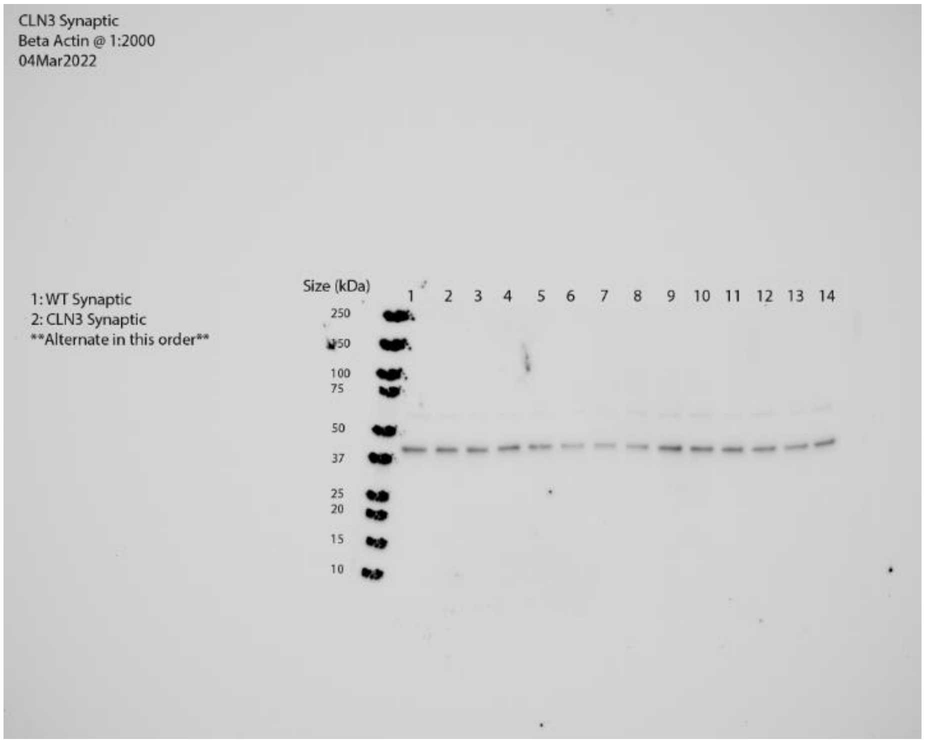
CLN3 Synaptic
Beta Actin @ 1:2000
04Mar2022
1: WT Synaptic
2: CLN3 Synaptic
Alternate in this order
Size (kDa)
1 2 3 4 5 6 7 8 9 10 11 12 13 14
250
150
100
75
50
37
25
20
15
10

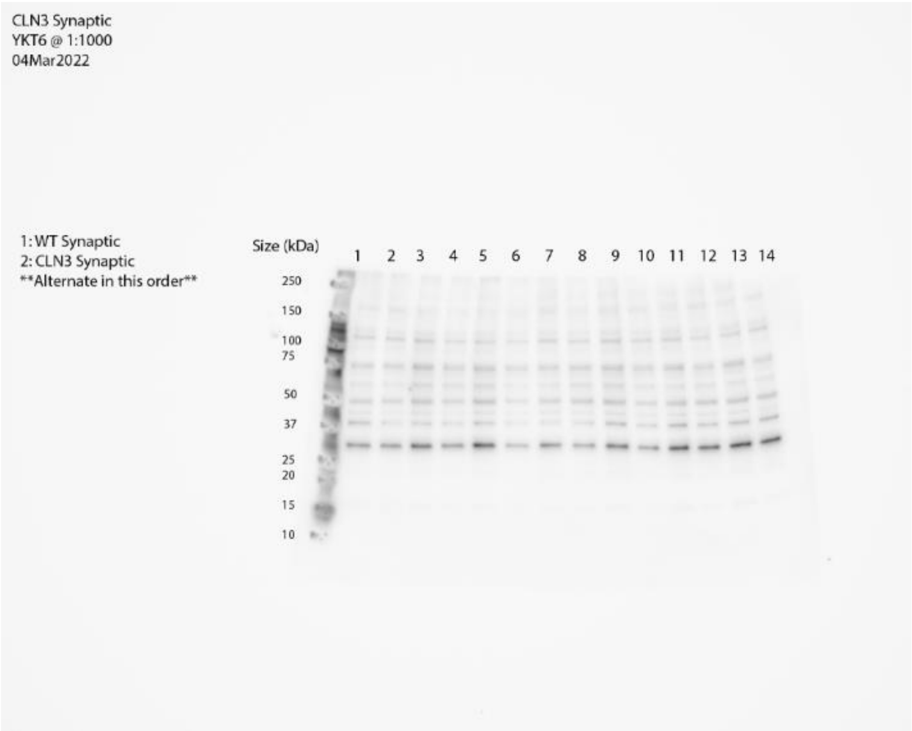
CLN3 Synaptic
YKT6 @ 1:1000
04Mar2022
1: WT Synaptic
2: CLN3 Synaptic
Alternate in this order
Size (kDa)
1 2 3 4 5 6 7 8 9 10 11 12 13 14
250
150
100
75
50
37
25
20
15
10

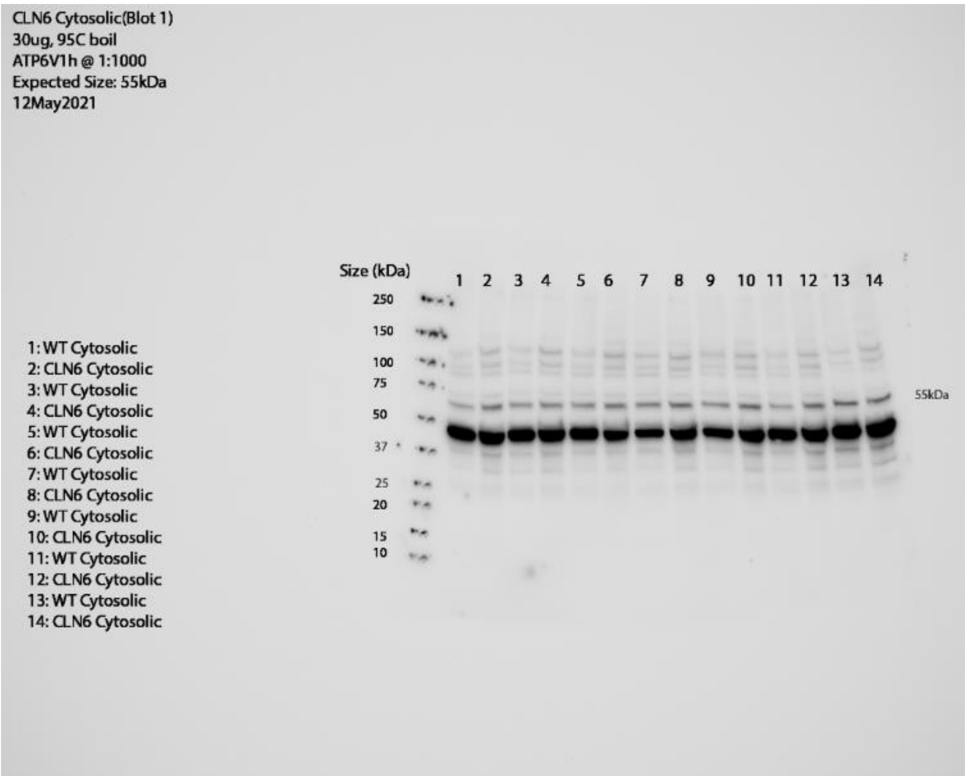
CLN6 Cytosolic(Blot 1)
30ug, 95C boil
ATP6V1h @ 1:1000
Expected Size: 55kDa
12May2021
Size (kDa)
1 2 3 4 5 6 7 8 9 10 11 12 13 14
250
150
100
75
50
37
25
20
15
10
55kDa
1: WT Cytosolic
2: CLN6 Cytosolic
3: WT Cytosolic
4: CLN6 Cytosolic
5: WT Cytosolic
6: CLN6 Cytosolic
7: WT Cytosolic
8: CLN6 Cytosolic
9: WT Cytosolic
10: CLN6 Cytosolic
11: WT Cytosolic
12: CLN6 Cytosolic
13: WT Cytosolic
14: CLN6 Cytosolic

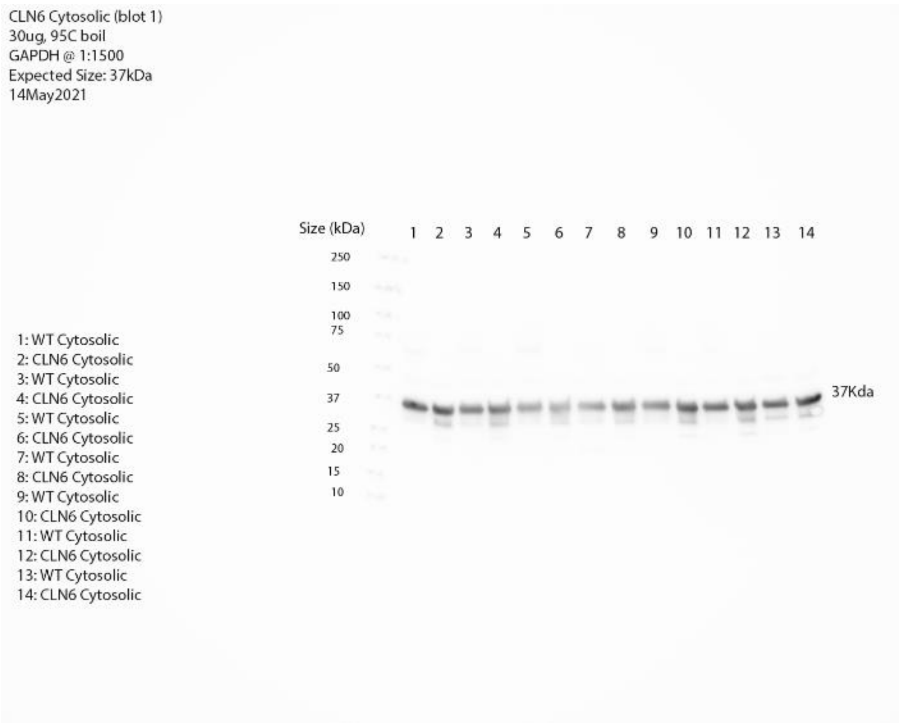
CLN6 Cytosolic (blot 1)
30ug, 95C boil
GAPDH @ 1:1500
Expected Size: 37kDa
14May2021
Size (kDa)
1 2 3 4 5 6 7 8 9 10 11 12 13 14
250
150
100
75
50
37
25
20
15
10
37Kda
1: WT Cytosolic
2: CLN6 Cytosolic
3: WT Cytosolic
4: CLN6 Cytosolic
5: WT Cytosolic
6: CLN6 Cytosolic
7: WT Cytosolic
8: CLN6 Cytosolic
9: WT Cytosolic
10: CLN6 Cytosolic
11: WT Cytosolic
12: CLN6 Cytosolic
13: WT Cytosolic
14: CLN6 Cytosolic

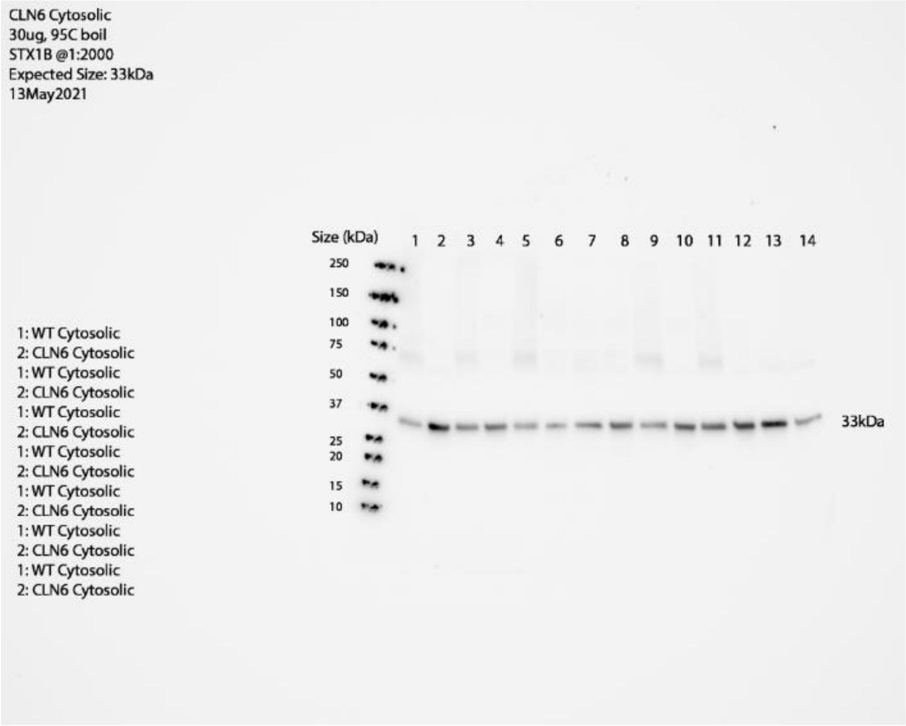
CLN6 Cytosolic
30ug, 95C boil
STX1B @1:2000
Expected Size: 33kDa
13May2021
Size (kDa)
1 2 3 4 5 6 7 8 9 10 11 12 13 14
250
150
100
75
50
37
25
20
15
10
33kDa
1: WT Cytosolic
2: CLN6 Cytosolic
1: WT Cytosolic
2: CLN6 Cytosolic
1: WT Cytosolic
2: CLN6 Cytosolic
1: WT Cytosolic
2: CLN6 Cytosolic
1: WT Cytosolic
2: CLN6 Cytosolic
1: WT Cytosolic
2: CLN6 Cytosolic
1: WT Cytosolic
2: CLN6 Cytosolic

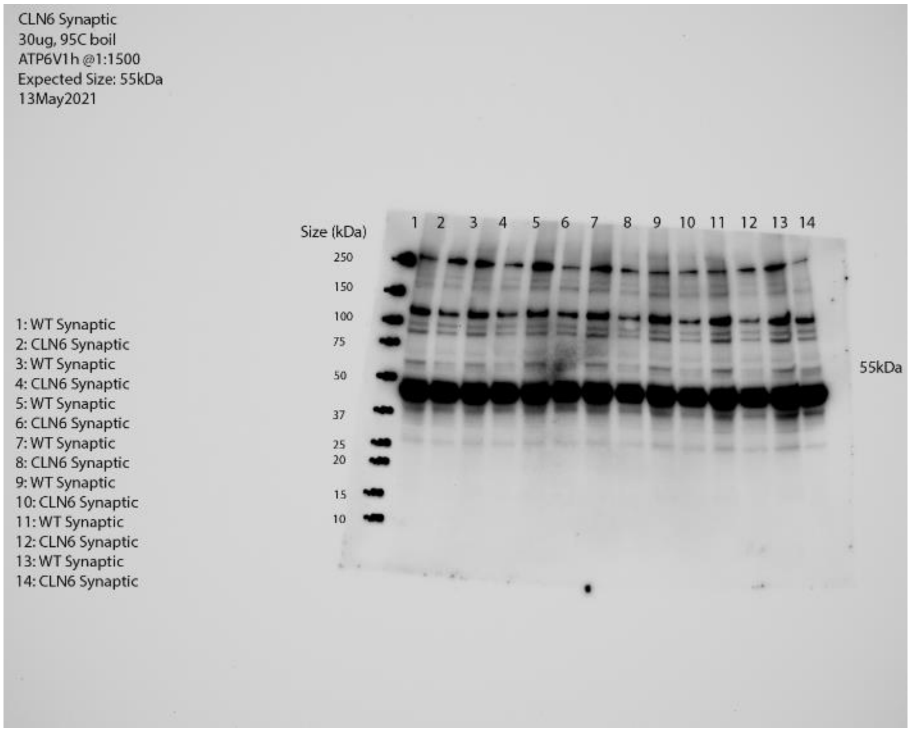
CLN6 Synaptic
30ug, 95C boil
ATP6V1h @1:1500
Expected Size: 55kDa
13May2021
Size (kDa)
1 2 3 4 5 6 7 8 9 10 11 12 13 14
250
150
100
75
50
37
25
20
15
10
55kDa
1: WT Synaptic
2: CLN6 Synaptic
3: WT Synaptic
4: CLN6 Synaptic
5: WT Synaptic
6: CLN6 Synaptic
7: WT Synaptic
8: CLN6 Synaptic
9: WT Synaptic
10: CLN6 Synaptic
11: WT Synaptic
12: CLN6 Synaptic
13: WT Synaptic
14: CLN6 Synaptic

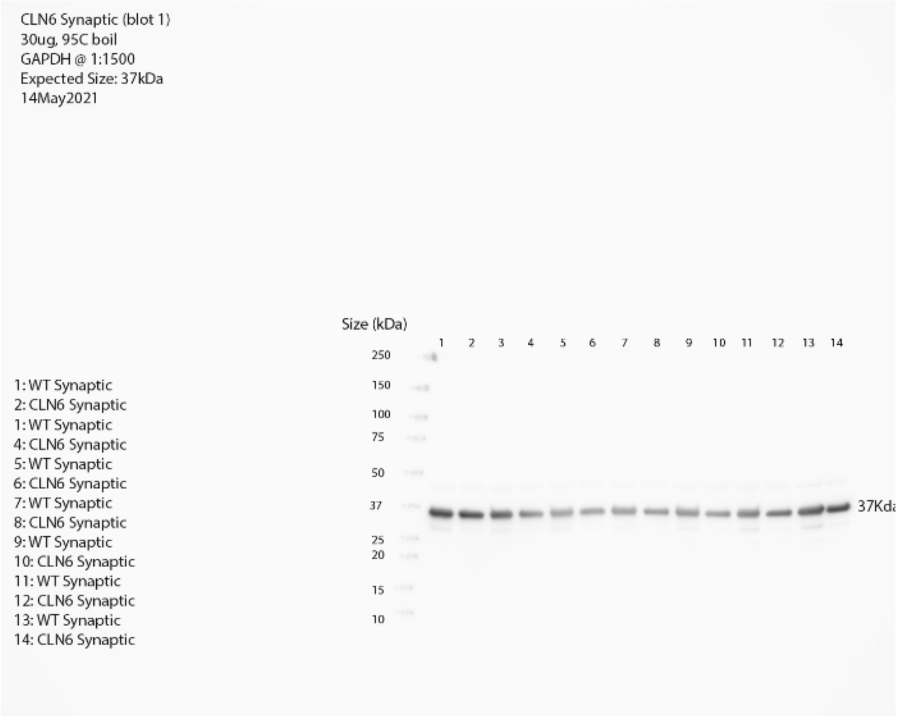
CLN6 Synaptic (blot 1)
30ug, 95C boil
GAPDH @ 1:1500
Expected Size: 37kDa
14May2021
Size (kDa)
1 2 3 4 5 6 7 8 9 10 11 12 13 14
250
150
100
75
50
37
25
20
15
10
37Kda
1: WT Synaptic
2: CLN6 Synaptic
1: WT Synaptic
4: CLN6 Synaptic
5: WT Synaptic
6: CLN6 Synaptic
7: WT Synaptic
8: CLN6 Synaptic
9: WT Synaptic
10: CLN6 Synaptic
11: WT Synaptic
12: CLN6 Synaptic
13: WT Synaptic
14: CLN6 Synaptic

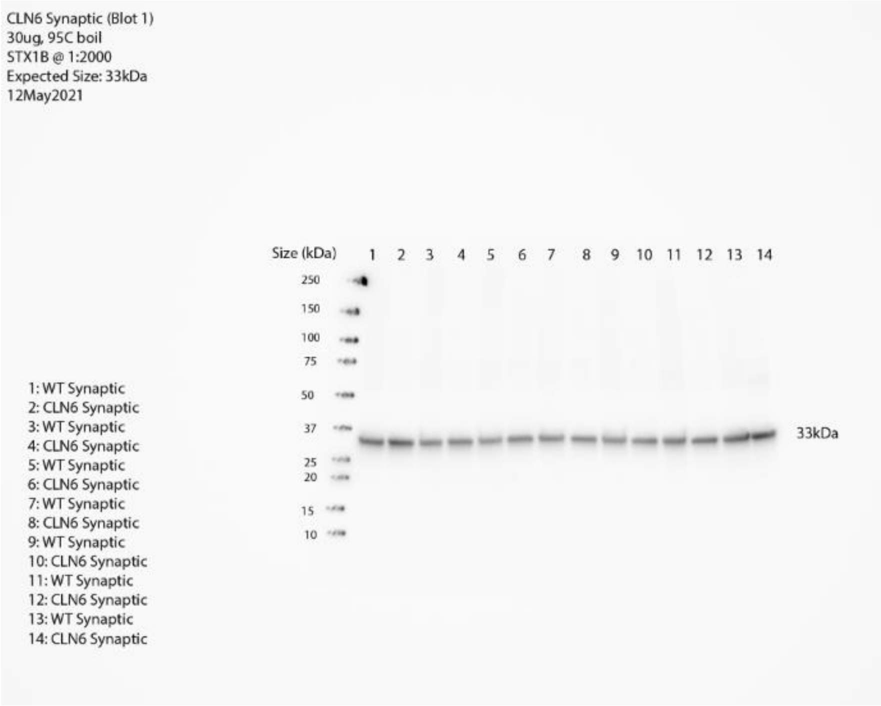
CLN6 Synaptic (Blot 1)
30ug, 95C boil
STX1B @ 1:2000
Expected Size: 33kDa
12May2021
Size (kDa)
1 2 3 4 5 6 7 8 9 10 11 12 13 14
250
150
100
75
50
37
25
20
15
10
33kDa
1: WT Synaptic
2: CLN6 Synaptic
3: WT Synaptic
4: CLN6 Synaptic
5: WT Synaptic
6: CLN6 Synaptic
7: WT Synaptic
8: CLN6 Synaptic
9: WT Synaptic
10: CLN6 Synaptic
11: WT Synaptic
12: CLN6 Synaptic
13: WT Synaptic
14: CLN6 Synaptic

CLN6 Cytosolic (blot 3)
30ug, 95C boil
Beta-Actin @ 1:1500
Expected Size: 45kDa
18May2021

1: WT Cytosolic
2: CLN6 Cytosolic
3: WT Cytosolic
4: CLN6 Cytosolic
5: WT Cytosolic
6: CLN6 Cytosolic
7: WT Cytosolic
8: CLN6 Cytosolic
9: WT Cytosolic
10: CLN6 Cytosolic
11: WT Cytosolic
12: CLN6 Cytosolic
13: WT Cytosolic
14: CLN6 Cytosolic

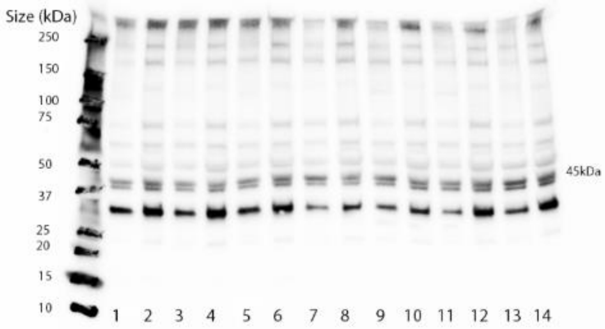

CLN6 Cytosolic (blot 3)
30ug, 95C boil
YKT6 @ 1:1000
Expected Size: 22kDa
14May2021

1: WT Cytosolic
2: CLN6 Cytosolic
3: WT Cytosolic
4: CLN6 Cytosolic
5: WT Cytosolic
6: CLN6 Cytosolic
7: WT Cytosolic
8: CLN6 Cytosolic
9: WT Cytosolic
10: CLN6 Cytosolic
11: WT Cytosolic
12: CLN6 Cytosolic
13: WT Cytosolic
14: CLN6 Cytosolic

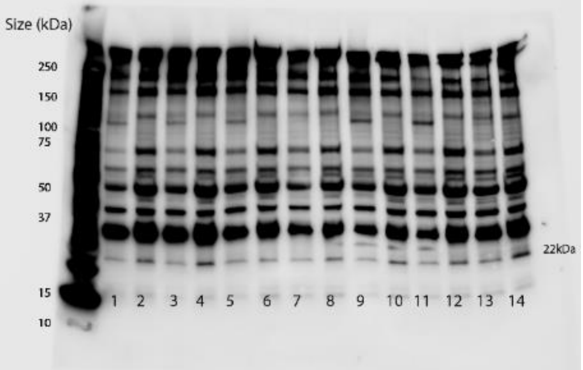

CLN6 Synaptic (blot 3)
30ug, 95C boil
Beta-Actin @ 1:1500
Expected Size: 45kDa
18May2021

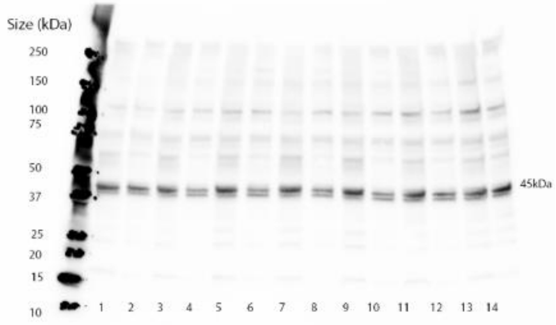

1: WT Synaptic
2: CLN6 Synaptic
3: WT Synaptic
4: CLN6 Synaptic
5: WT Synaptic
6: CLN6 Synaptic
7: WT Synaptic
8: CLN6 Synaptic
9: WT Synaptic
10: CLN6 Synaptic
11: WT Synaptic
12: CLN6 Synaptic
13: WT Synaptic
14: CLN6 Synaptic

CLN6 Synaptic (blot 3)
30ug, 95C boil
YKT6 @ 1:1000
Expected Size: 22kDa
14May2021

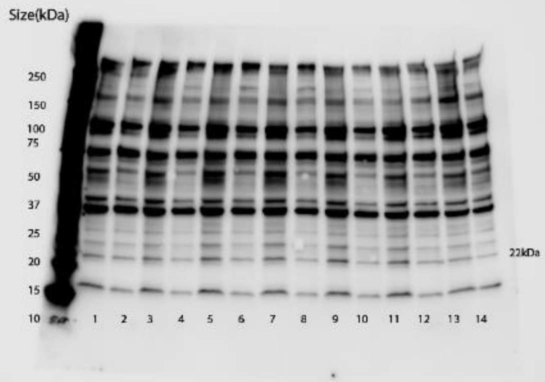

1: WT Synaptic
2: CLN6 Synaptic
1: WT Synaptic
4: CLN6 Synaptic
5: WT Synaptic
6: CLN6 Synaptic
7: WT Synaptic
8: CLN6 Synaptic
9: WT Synaptic
10: CLN6 Synaptic
11: WT Synaptic
12: CLN6 Synaptic
13: WT Synaptic
14: CLN6 Synaptic

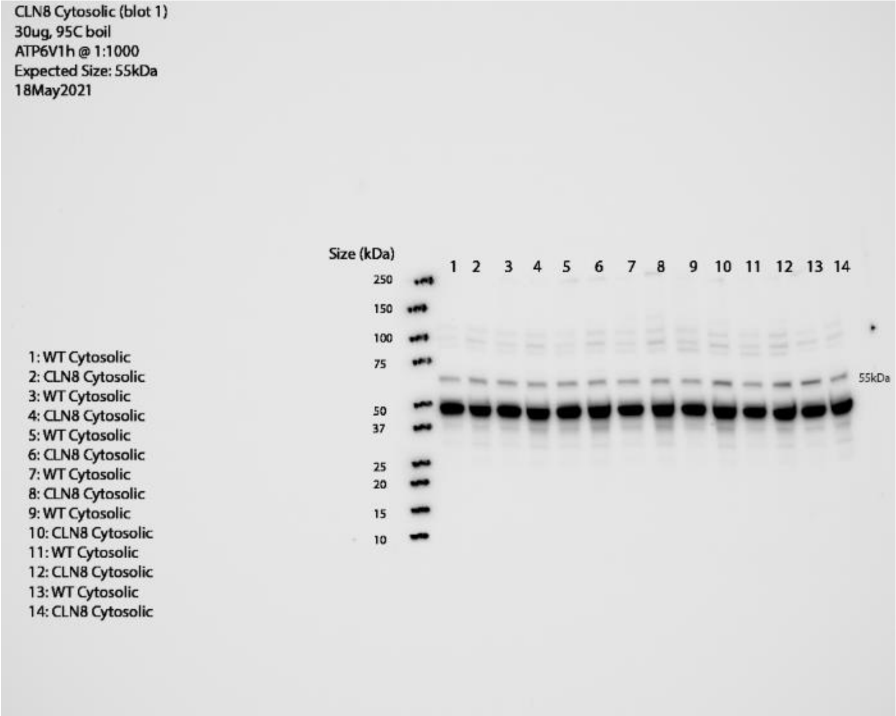
CLN8 Cytosolic (blot 1)
30ug, 95C boil
ATP6V1h @ 1:1000
Expected Size: 55kDa
18May2021
Size (kDa)
250
150
100
75
50
37
25
20
15
10
1 2 3 4 5 6 7 8 9 10 11 12 13 14
55kDa
1: WT Cytosolic
2: CLN8 Cytosolic
3: WT Cytosolic
4: CLN8 Cytosolic
5: WT Cytosolic
6: CLN8 Cytosolic
7: WT Cytosolic
8: CLN8 Cytosolic
9: WT Cytosolic
10: CLN8 Cytosolic
11: WT Cytosolic
12: CLN8 Cytosolic
13: WT Cytosolic
14: CLN8 Cytosolic

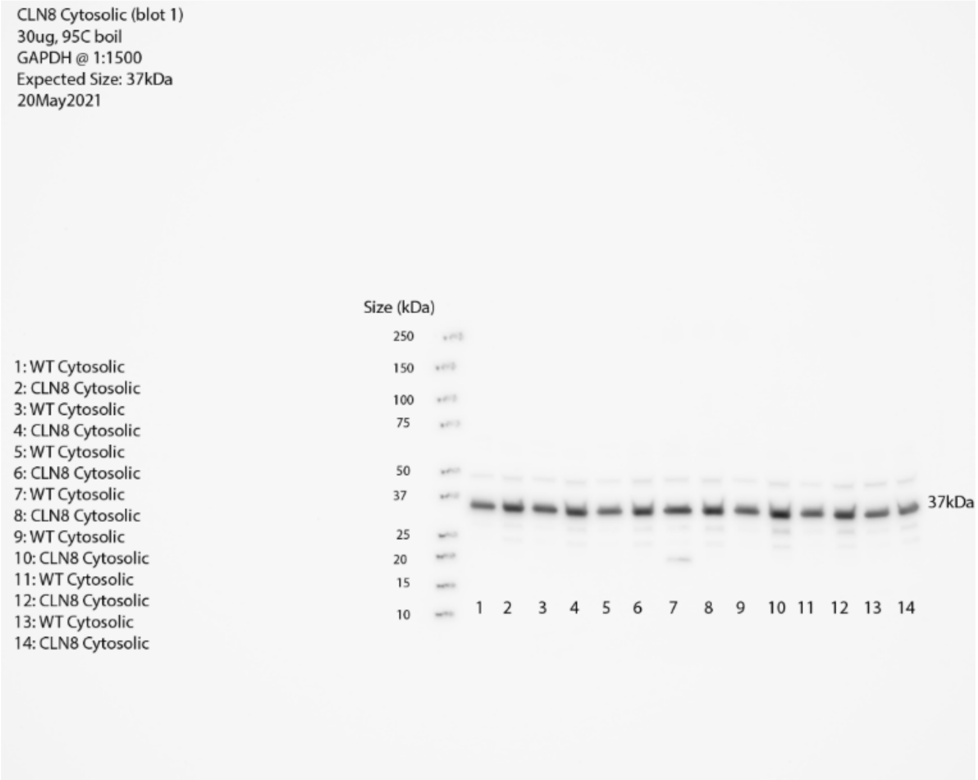
CLN8 Cytosolic (blot 1)
30ug, 95C boil
GAPDH @ 1:1500
Expected Size: 37kDa
20May2021
Size (kDa)
250
150
100
75
50
37
25
20
15
10
37kDa
1 2 3 4 5 6 7 8 9 10 11 12 13 14
1: WT Cytosolic
2: CLN8 Cytosolic
3: WT Cytosolic
4: CLN8 Cytosolic
5: WT Cytosolic
6: CLN8 Cytosolic
7: WT Cytosolic
8: CLN8 Cytosolic
9: WT Cytosolic
10: CLN8 Cytosolic
11: WT Cytosolic
12: CLN8 Cytosolic
13: WT Cytosolic
14: CLN8 Cytosolic

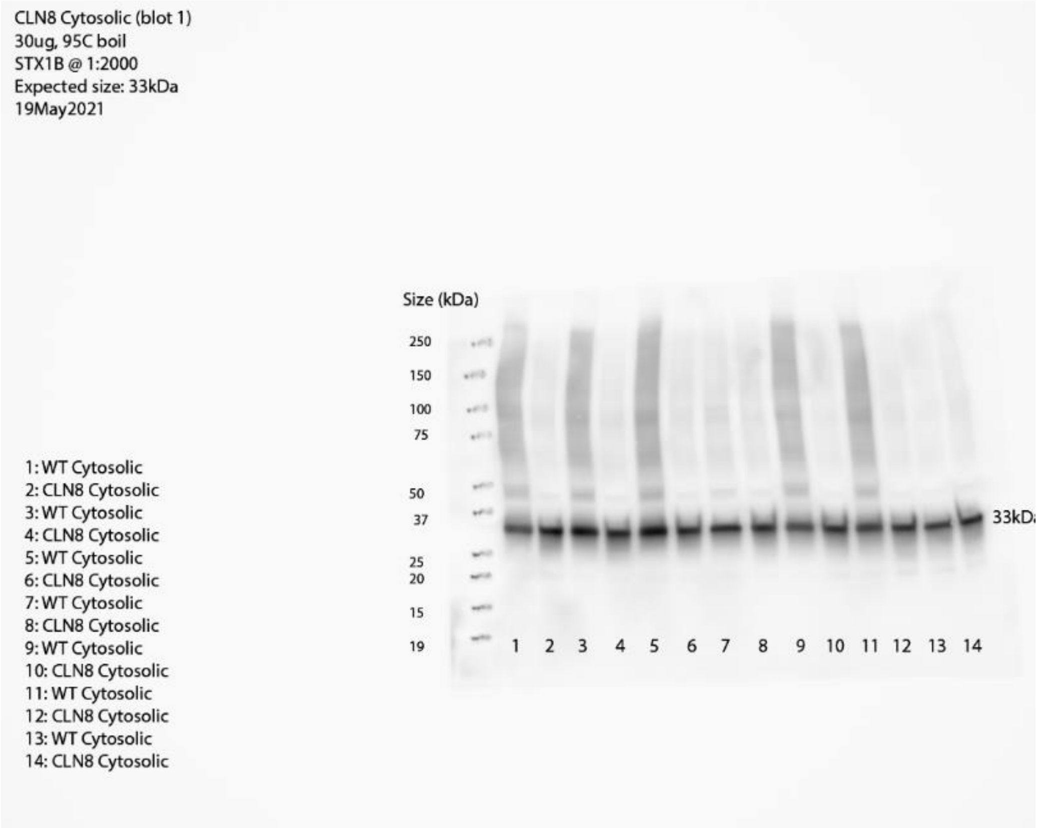
CLN8 Cytosolic (blot 1)
30ug, 95C boil
STX1B @ 1:2000
Expected size: 33kDa
19May2021
Size (kDa)
250
150
100
75
50
37
25
20
15
19
33kD
1 2 3 4 5 6 7 8 9 10 11 12 13 14
1: WT Cytosolic
2: CLN8 Cytosolic
3: WT Cytosolic
4: CLN8 Cytosolic
5: WT Cytosolic
6: CLN8 Cytosolic
7: WT Cytosolic
8: CLN8 Cytosolic
9: WT Cytosolic
10: CLN8 Cytosolic
11: WT Cytosolic
12: CLN8 Cytosolic
13: WT Cytosolic
14: CLN8 Cytosolic

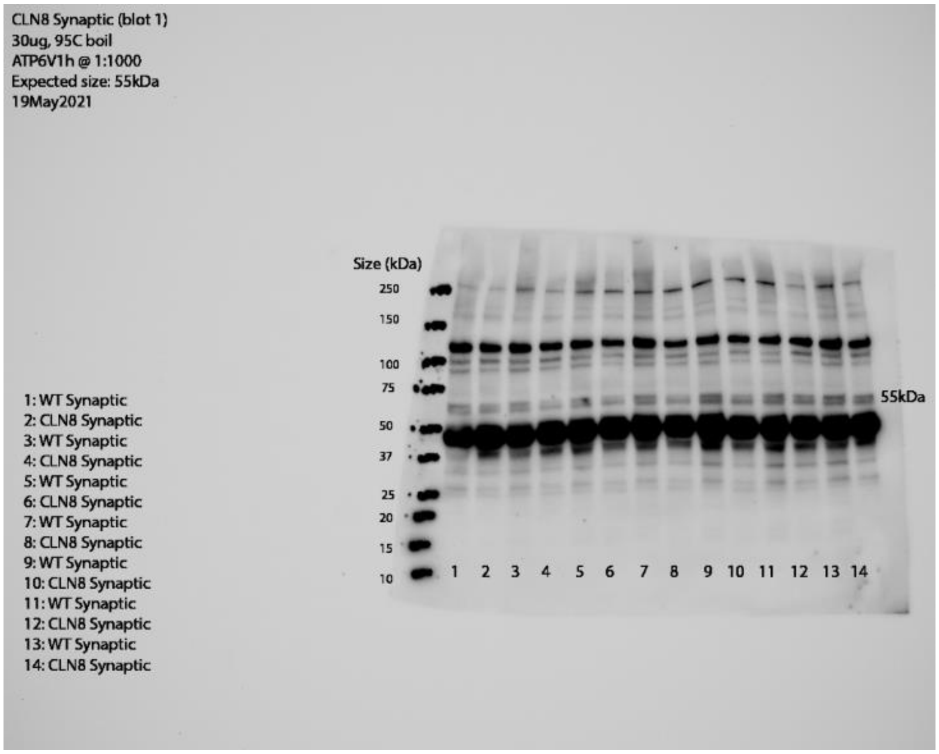
CLN8 Synaptic (blot 1)
30ug, 95C boil
ATP6V1h @ 1:1000
Expected size: 55kDa
19May2021
Size (kDa)
250
150
100
75
50
37
25
20
15
10
55kDa
1 2 3 4 5 6 7 8 9 10 11 12 13 14
1: WT Synaptic
2: CLN8 Synaptic
3: WT Synaptic
4: CLN8 Synaptic
5: WT Synaptic
6: CLN8 Synaptic
7: WT Synaptic
8: CLN8 Synaptic
9: WT Synaptic
10: CLN8 Synaptic
11: WT Synaptic
12: CLN8 Synaptic
13: WT Synaptic
14: CLN8 Synaptic

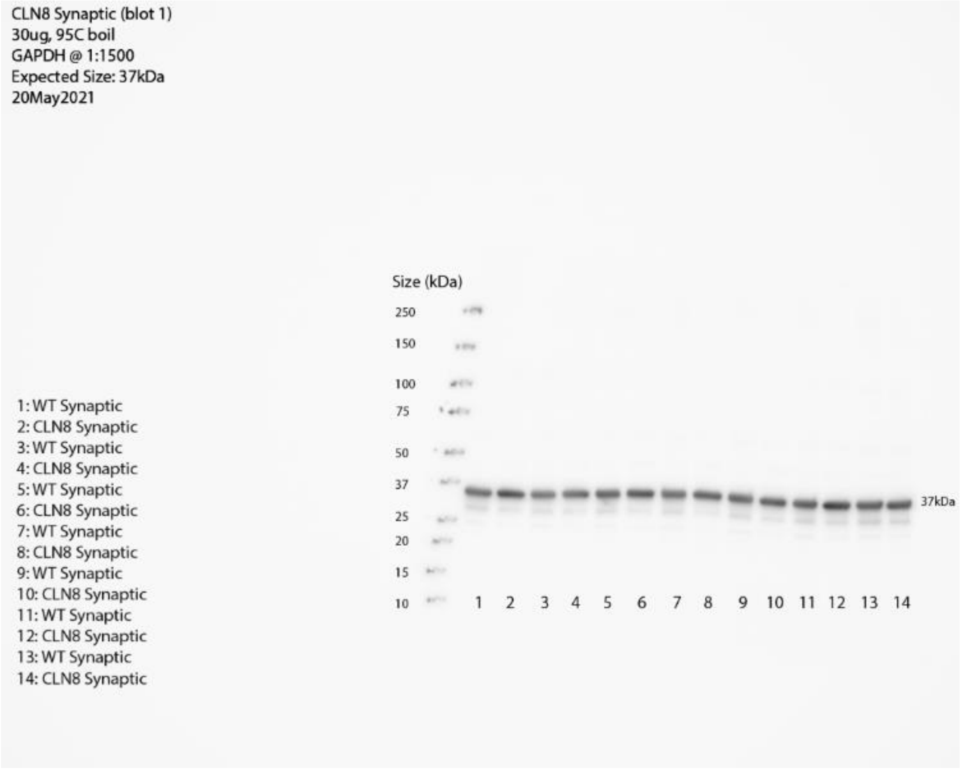
CLN8 Synaptic (blot 1)
30ug, 95C boil
GAPDH @ 1:1500
Expected Size: 37kDa
20May2021
Size (kDa)
250
150
100
75
50
37
25
20
15
10
1 2 3 4 5 6 7 8 9 10 11 12 13 14
37kDa
1: WT Synaptic
2: CLN8 Synaptic
3: WT Synaptic
4: CLN8 Synaptic
5: WT Synaptic
6: CLN8 Synaptic
7: WT Synaptic
8: CLN8 Synaptic
9: WT Synaptic
10: CLN8 Synaptic
11: WT Synaptic
12: CLN8 Synaptic
13: WT Synaptic
14: CLN8 Synaptic

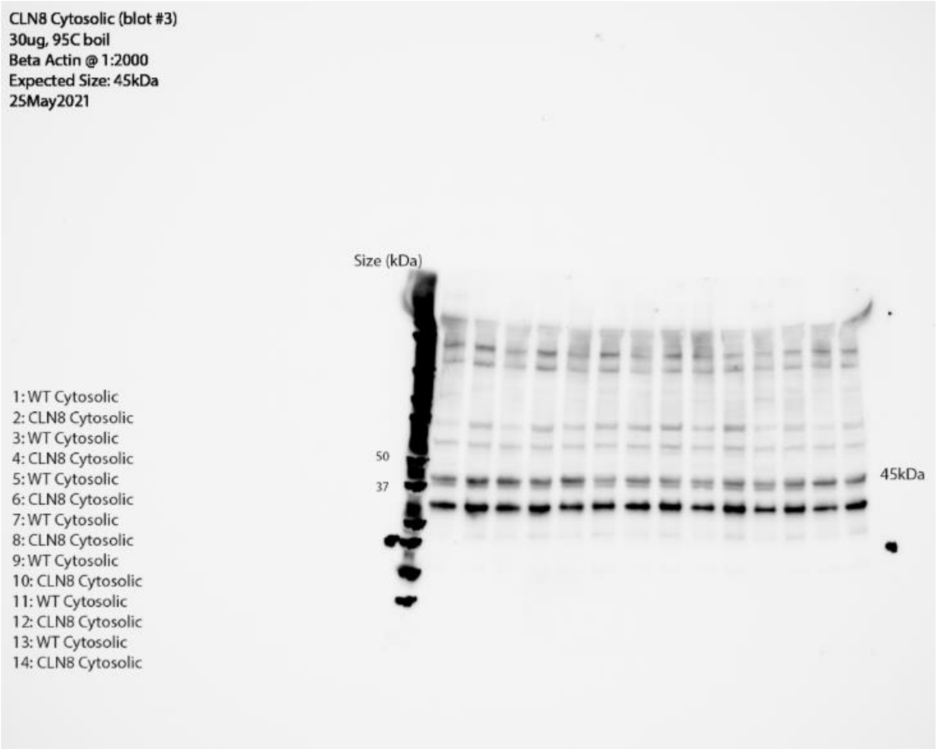
CLN8 Cytosolic (blot #3)
30ug, 95C boil
Beta Actin @ 1:2000
Expected Size: 45kDa
25May2021
Size (kDa)
50
37
45kDa
1: WT Cytosolic
2: CLN8 Cytosolic
3: WT Cytosolic
4: CLN8 Cytosolic
5: WT Cytosolic
6: CLN8 Cytosolic
7: WT Cytosolic
8: CLN8 Cytosolic
9: WT Cytosolic
10: CLN8 Cytosolic
11: WT Cytosolic
12: CLN8 Cytosolic
13: WT Cytosolic
14: CLN8 Cytosolic

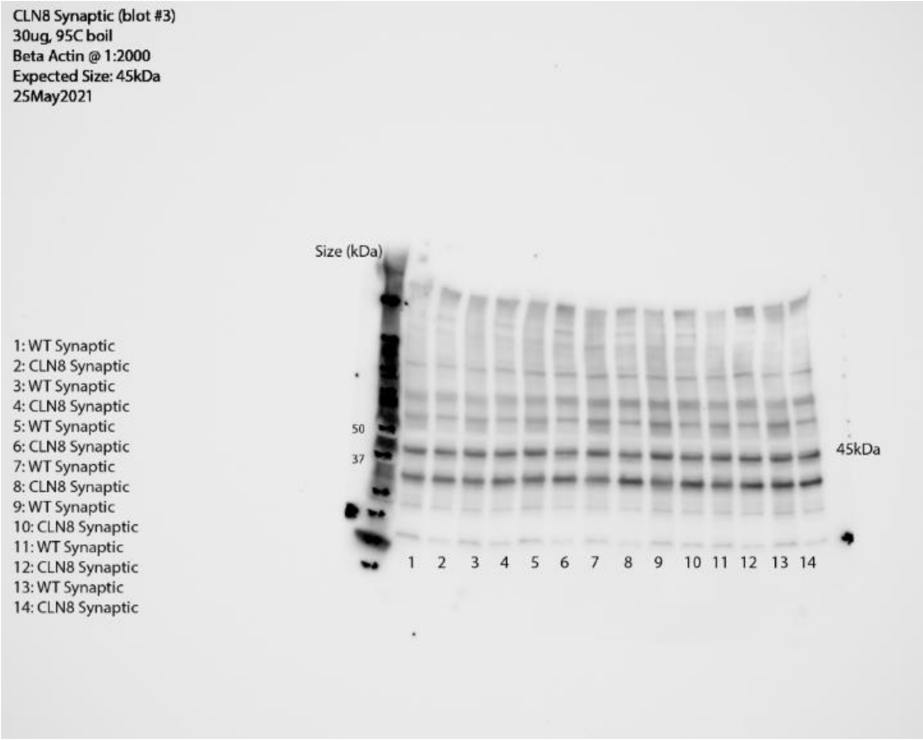
CLN8 Synaptic (blot #3)
30ug, 95C boil
Beta Actin @ 1:2000
Expected Size: 45kDa
25May2021
1: WT Synaptic
2: CLN8 Synaptic
3: WT Synaptic
4: CLN8 Synaptic
5: WT Synaptic
6: CLN8 Synaptic
7: WT Synaptic
8: CLN8 Synaptic
9: WT Synaptic
10: CLN8 Synaptic
11: WT Synaptic
12: CLN8 Synaptic
13: WT Synaptic
14: CLN8 Synaptic
Size (kDa)
50
37
45kDa
1 2 3 4 5 6 7 8 9 10 11 12 13 14

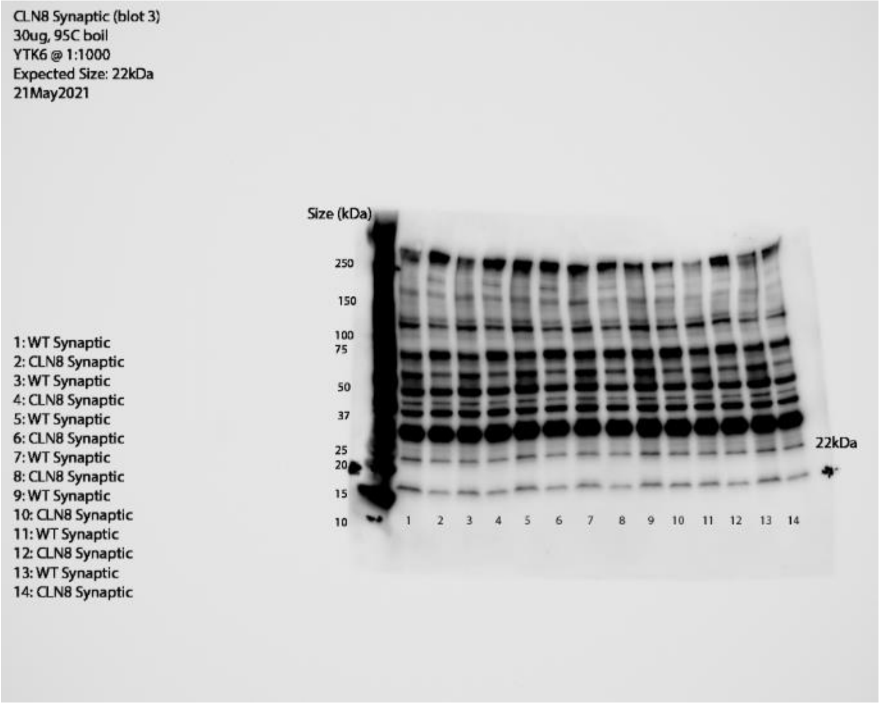
CLN8 Synaptic (blot 3)
30ug, 95C boil
YTK6 @ 1:1000
Expected Size: 22kDa
21May2021
1: WT Synaptic
2: CLN8 Synaptic
3: WT Synaptic
4: CLN8 Synaptic
5: WT Synaptic
6: CLN8 Synaptic
7: WT Synaptic
8: CLN8 Synaptic
9: WT Synaptic
10: CLN8 Synaptic
11: WT Synaptic
12: CLN8 Synaptic
13: WT Synaptic
14: CLN8 Synaptic
Size (kDa)
250
150
100
75
50
37
25
20
15
10
22kDa
1 2 3 4 5 6 7 8 9 10 11 12 13 14

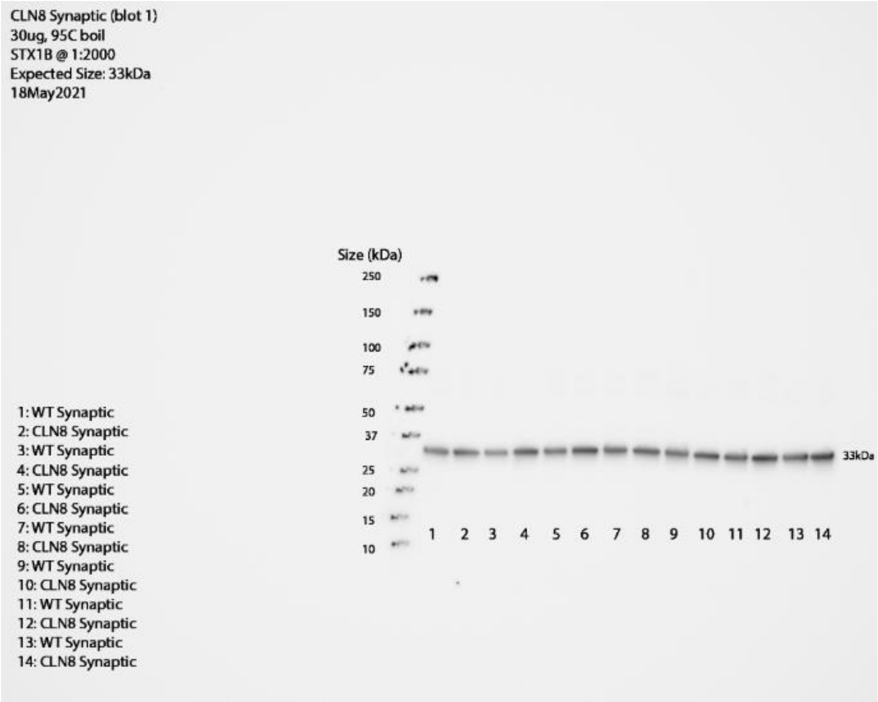

CLN8 Synaptic (blot 1)
30ug, 95C boil
STX1B @ 1:2000
Expected Size: 33kDa
18May2021
Size (kDa)
250
150
100
75
50
37
25
20
15
10
33kDa
1 2 3 4 5 6 7 8 9 10 11 12 13 14
1: WT Synaptic
2: CLN8 Synaptic
3: WT Synaptic
4: CLN8 Synaptic
5: WT Synaptic
6: CLN8 Synaptic
7: WT Synaptic
8: CLN8 Synaptic
9: WT Synaptic
10: CLN8 Synaptic
11: WT Synaptic
12: CLN8 Synaptic
13: WT Synaptic
14: CLN8 Synaptic

Uncropped blots from Figure 3

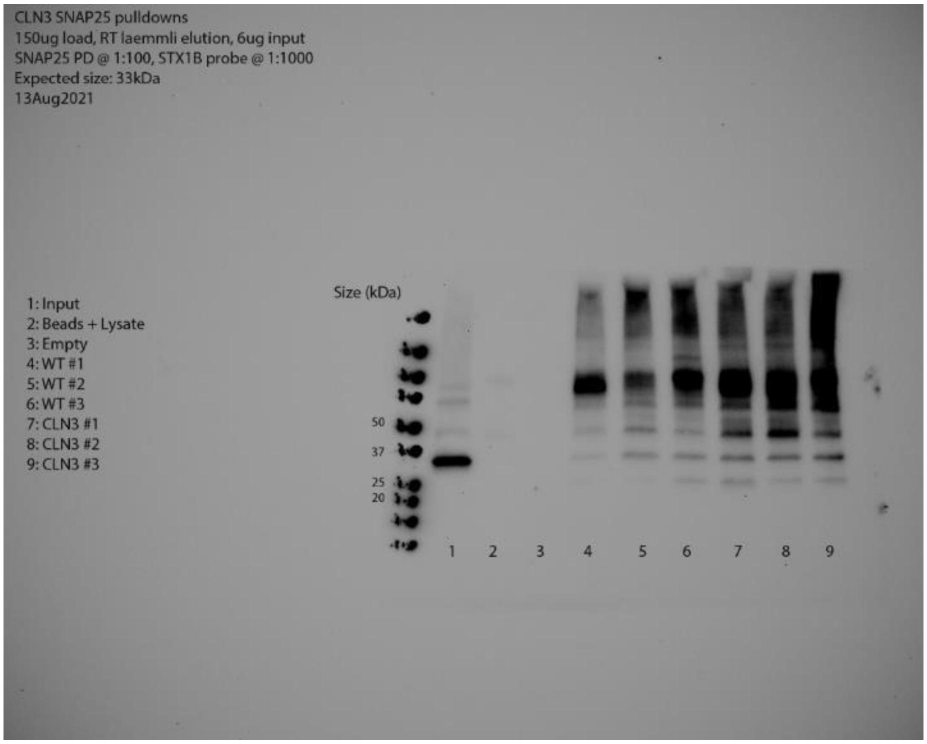
CLN3 SNAP25 pulldowns
150ug load, RT laemmli elution, 6ug input
SNAP25 PD @ 1:100, STX1B probe @ 1:1000
Expected size: 33kDa
13Aug2021
1: Input
2: Beads + Lysate
3: Empty
4: WT #1
5: WT #2
6: WT #3
7: CLN3 #1
8: CLN3 #2
9: CLN3 #3
Size (kDa)
50
37
25
20
1 2 3 4 5 6 7 8 9

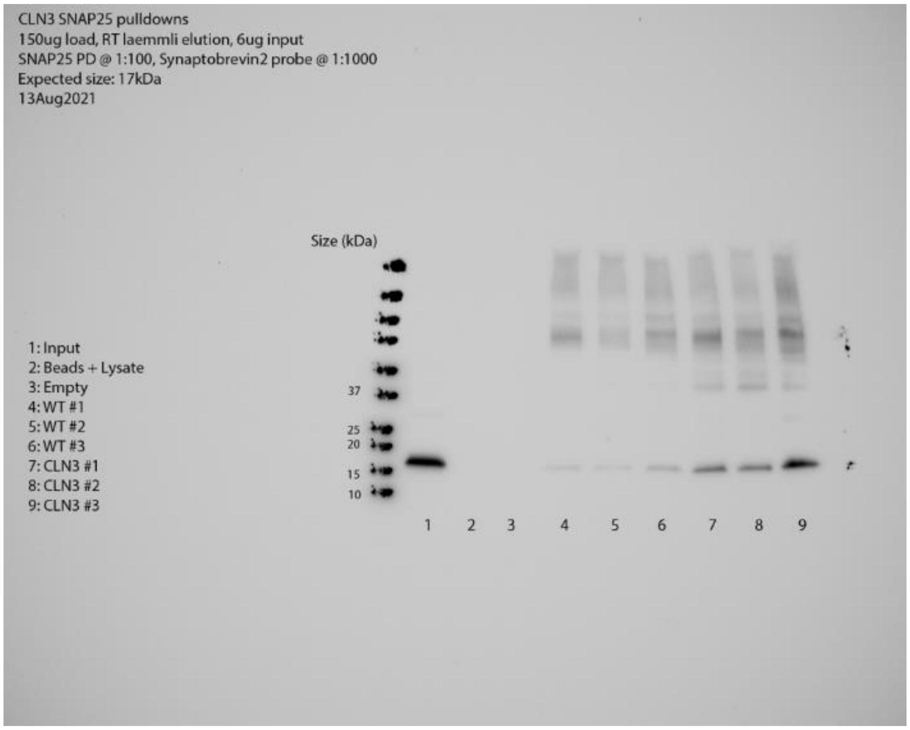
CLN3 SNAP25 pulldowns
150ug load, RT laemmli elution, 6ug input
SNAP25 PD @ 1:100, Synaptobrevin2 probe @ 1:1000
Expected size: 17kDa
13Aug2021
1: Input
2: Beads + Lysate
3: Empty
4: WT #1
5: WT #2
6: WT #3
7: CLN3 #1
8: CLN3 #2
9: CLN3 #3
Size (kDa)
37
25
20
15
10
1 2 3 4 5 6 7 8 9

CLN3 SNAP25 pulldowns
150ug load, RT laemmli elution, 6ug input
SNAP25 PD @ 1:100, Syntaxin1 probe @ 1:1000
Expected size: 33kDa
13Aug2021

1: Input
2: Beads + Lysate
3: Empty
4: WT #1
5: WT #2
6: WT #3
7: CLN3 #1
8: CLN3 #2
9: CLN3 #3

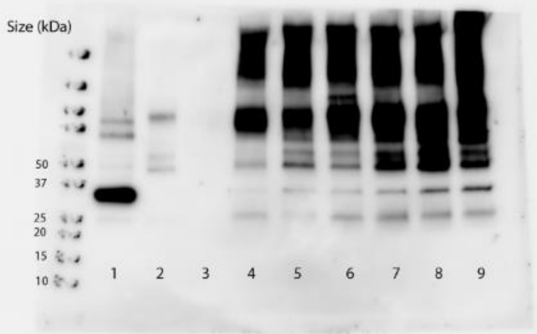

CLN6 FXNL CoIP
150 ug load, RT laemmli elution, 6ug input
SNAP25 PD @ 1:100, STX1B probe @ 1:1000
Expected size: 33kDa
06Aug2021

1: Input
2: Beads + Lysate
3: Empty
4: WT #1
5: WT #2
6: WT #3
7: CLN6 #1
8: CLN6 #2
9: CLN6 #3

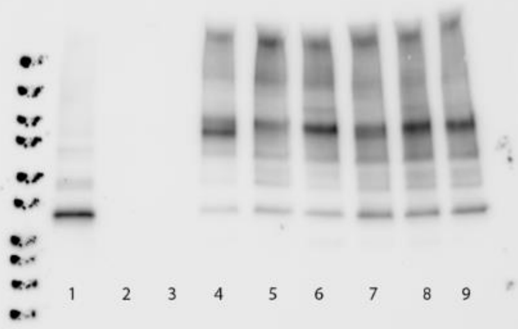

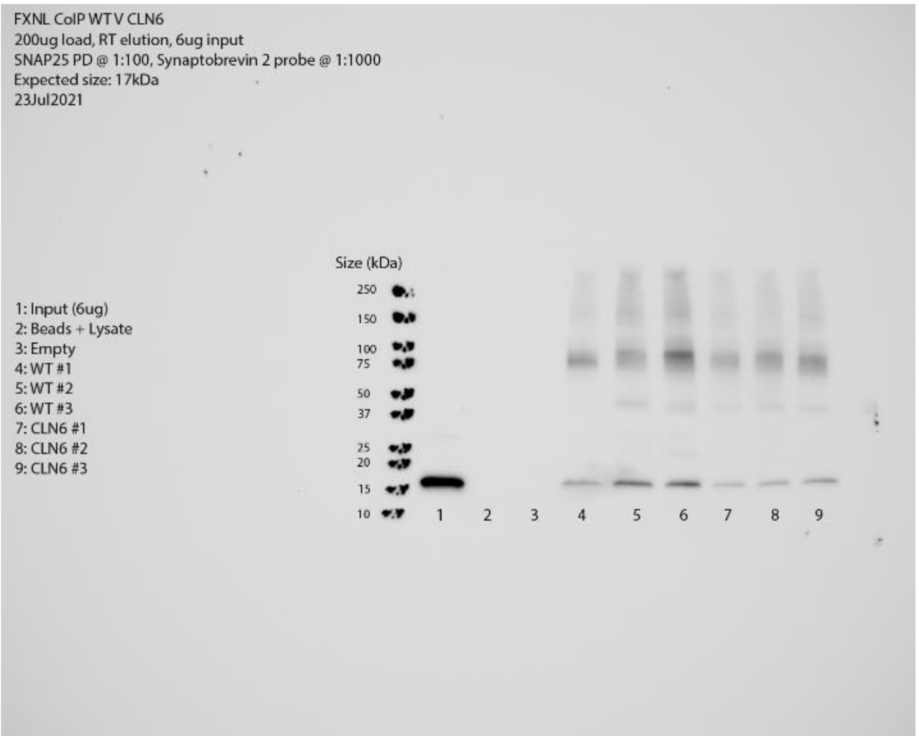
FXNL CoIP WT V CLN6
200ug load, RT elution, 6ug input
SNAP25 PD @ 1:100, Synaptobrevin 2 probe @ 1:1000
Expected size: 17kDa
23Jul2021
Size (kDa)
250
150
100
75
50
37
25
20
15
10
1: Input (6ug)
2: Beads + Lysate
3: Empty
4: WT #1
5: WT #2
6: WT #3
7: CLN6 #1
8: CLN6 #2
9: CLN6 #3
1 2 3 4 5 6 7 8 9

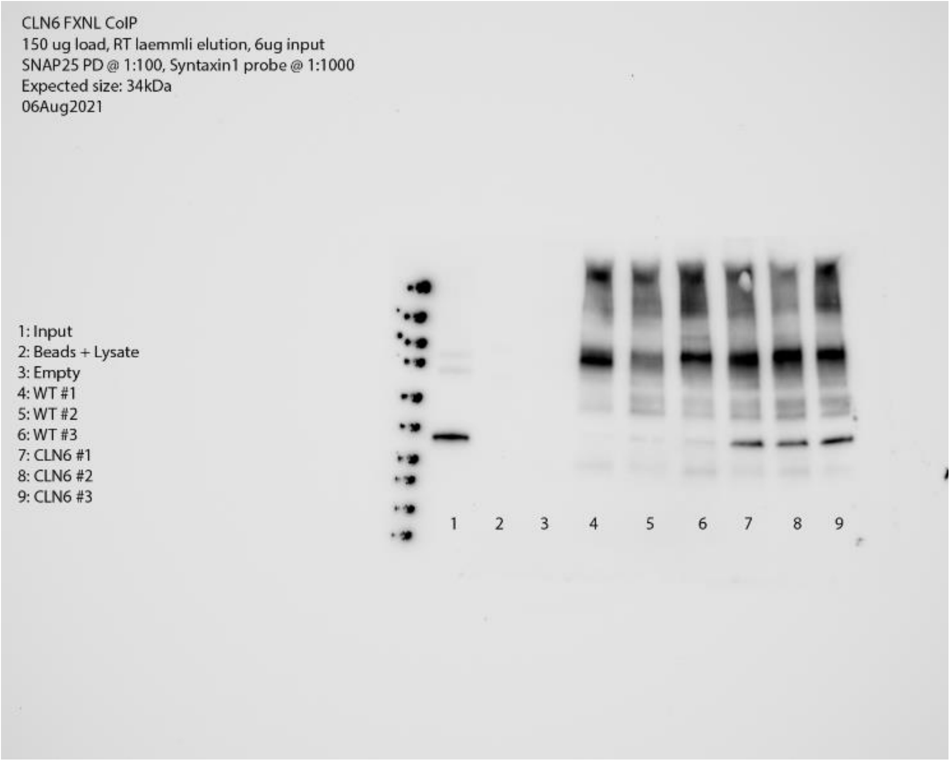
CLN6 FXNL CoIP
150 ug load, RT laemmli elution, 6ug input
SNAP25 PD @ 1:100, Syntaxin1 probe @ 1:1000
Expected size: 34kDa
06Aug2021
1: Input
2: Beads + Lysate
3: Empty
4: WT #1
5: WT #2
6: WT #3
7: CLN6 #1
8: CLN6 #2
9: CLN6 #3
1 2 3 4 5 6 7 8 9

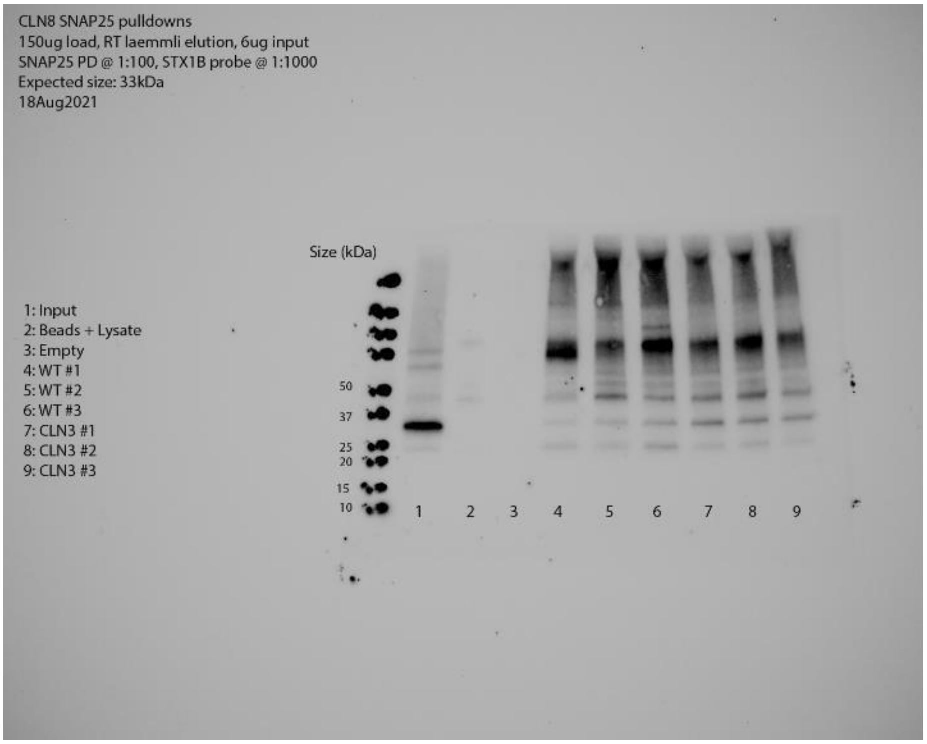
CLN8 SNAP25 pulldowns
150ug load, RT laemmli elution, 6ug input
SNAP25 PD @ 1:100, STX1B probe @ 1:1000
Expected size: 33kDa
18Aug2021
Size (kDa)
1: Input
2: Beads + Lysate
3: Empty
4: WT #1
5: WT #2
6: WT #3
7: CLN3 #1
8: CLN3 #2
9: CLN3 #3
50
37
25
20
15
10
1
2
3
4
5
6
7
8
9

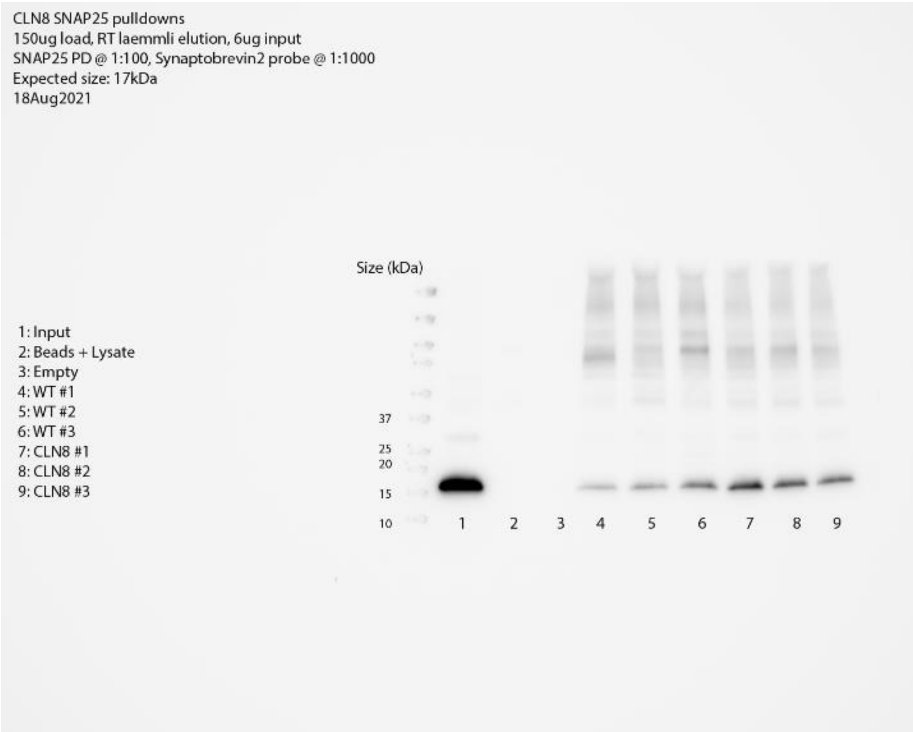
CLN8 SNAP25 pulldowns
150ug load, RT laemmli elution, 6ug input
SNAP25 PD @ 1:100, Synaptobrevin2 probe @ 1:1000
Expected size: 17kDa
18Aug2021
Size (kDa)
1: Input
2: Beads + Lysate
3: Empty
4: WT #1
5: WT #2
6: WT #3
7: CLN8 #1
8: CLN8 #2
9: CLN8 #3
37
25
20
15
10
1
2
3
4
5
6
7
8
9

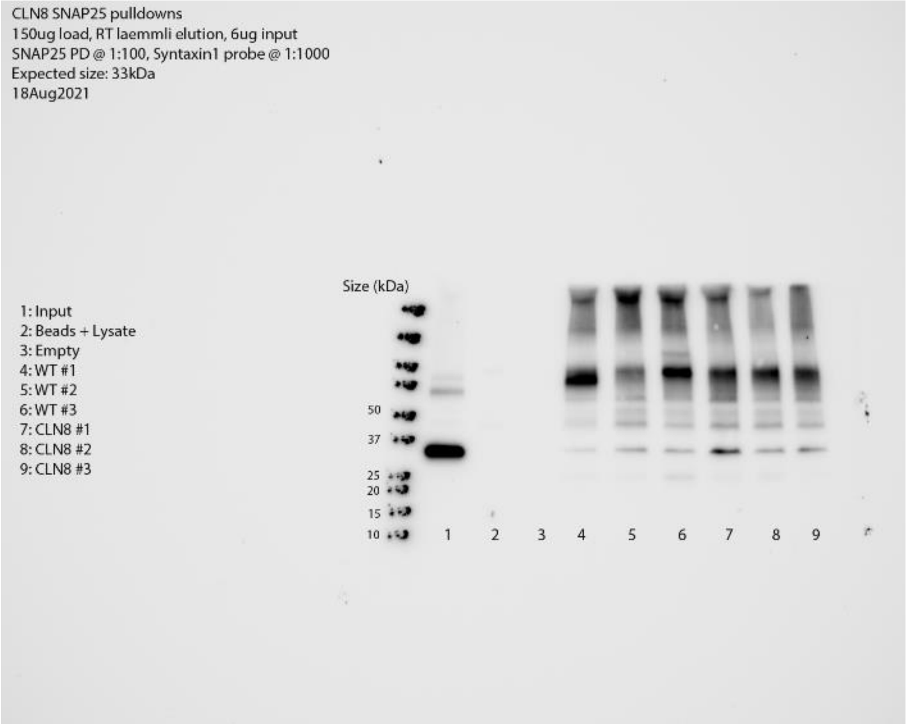
CLN8 SNAP25 pulldowns
150ug load, RT laemmli elution, 6ug input
SNAP25 PD @ 1:100, Syntaxin1 probe @ 1:1000
Expected size: 33kDa
18Aug2021
1: Input
2: Beads + Lysate
3: Empty
4: WT #1
5: WT #2
6: WT #3
7: CLN8 #1
8: CLN8 #2
9: CLN8 #3
Size (kDa)
50
37
25
20
15
10
1 2 3 4 5 6 7 8 9

Blots from Supplemental Figure 2 (that were not included in figure 2)

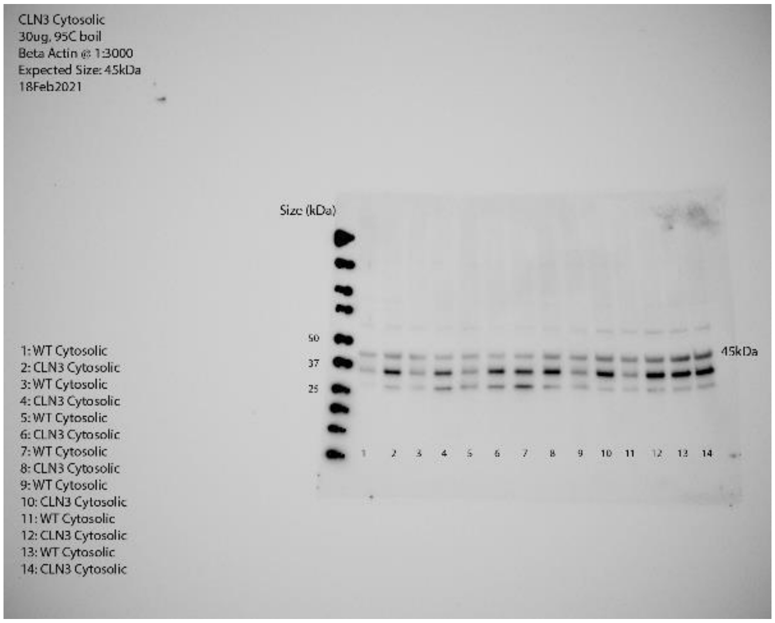
CLN3 Cytosolic
30ug, 95C boil
Beta Actin @ 1:3000
Expected Size: 45kDa
18Feb2021
Size (kDa)
50
37
25
45kDa
1 2 3 4 5 6 7 8 9 10 11 12 13 14
1: WT Cytosolic
2: CLN3 Cytosolic
3: WT Cytosolic
4: CLN3 Cytosolic
5: WT Cytosolic
6: CLN3 Cytosolic
7: WT Cytosolic
8: CLN3 Cytosolic
9: WT Cytosolic
10: CLN3 Cytosolic
11: WT Cytosolic
12: CLN3 Cytosolic
13: WT Cytosolic
14: CLN3 Cytosolic

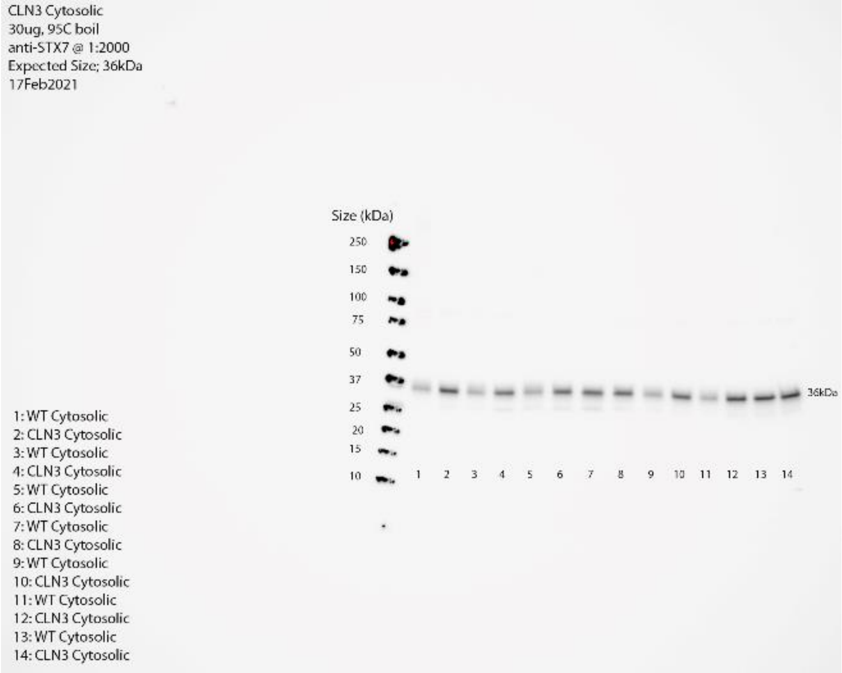
CLN3 Cytosolic
30ug, 95C boil
anti-STX7 @ 1:2000
Expected Size; 36kDa
17Feb2021
Size (kDa)
250
150
100
75
50
37
25
20
15
10
36kDa
1 2 3 4 5 6 7 8 9 10 11 12 13 14
1: WT Cytosolic
2: CLN3 Cytosolic
3: WT Cytosolic
4: CLN3 Cytosolic
5: WT Cytosolic
6: CLN3 Cytosolic
7: WT Cytosolic
8: CLN3 Cytosolic
9: WT Cytosolic
10: CLN3 Cytosolic
11: WT Cytosolic
12: CLN3 Cytosolic
13: WT Cytosolic
14: CLN3 Cytosolic

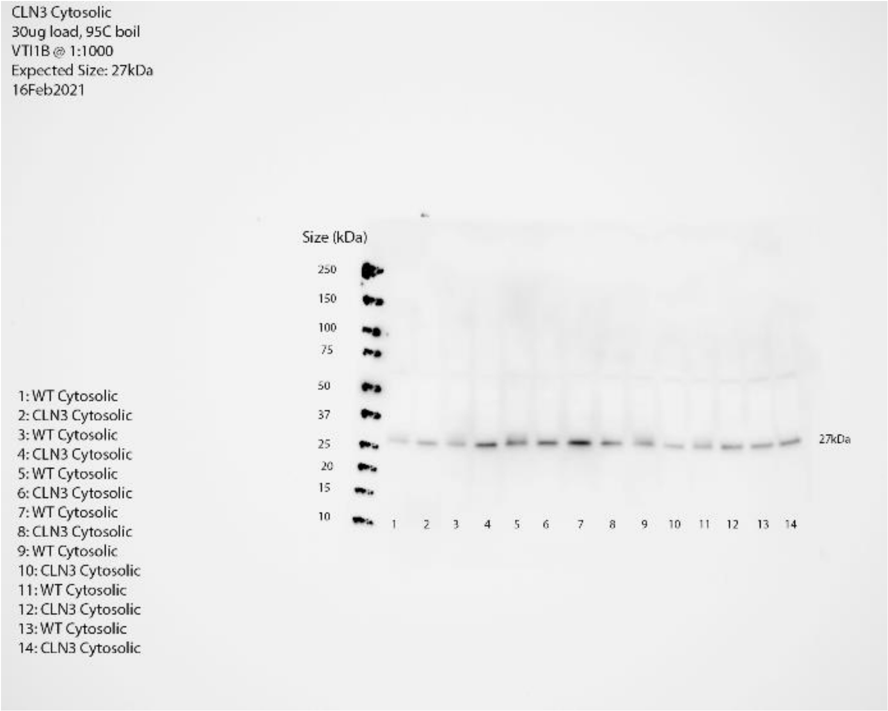
CLN3 Cytosolic
30ug load, 95C boil
VTI1B @ 1:1000
Expected Size: 27kDa
16Feb2021
Size (kDa)
250
150
100
75
50
37
25
20
15
10
27kDa
1 2 3 4 5 6 7 8 9 10 11 12 13 14
1: WT Cytosolic
2: CLN3 Cytosolic
3: WT Cytosolic
4: CLN3 Cytosolic
5: WT Cytosolic
6: CLN3 Cytosolic
7: WT Cytosolic
8: CLN3 Cytosolic
9: WT Cytosolic
10: CLN3 Cytosolic
11: WT Cytosolic
12: CLN3 Cytosolic
13: WT Cytosolic
14: CLN3 Cytosolic

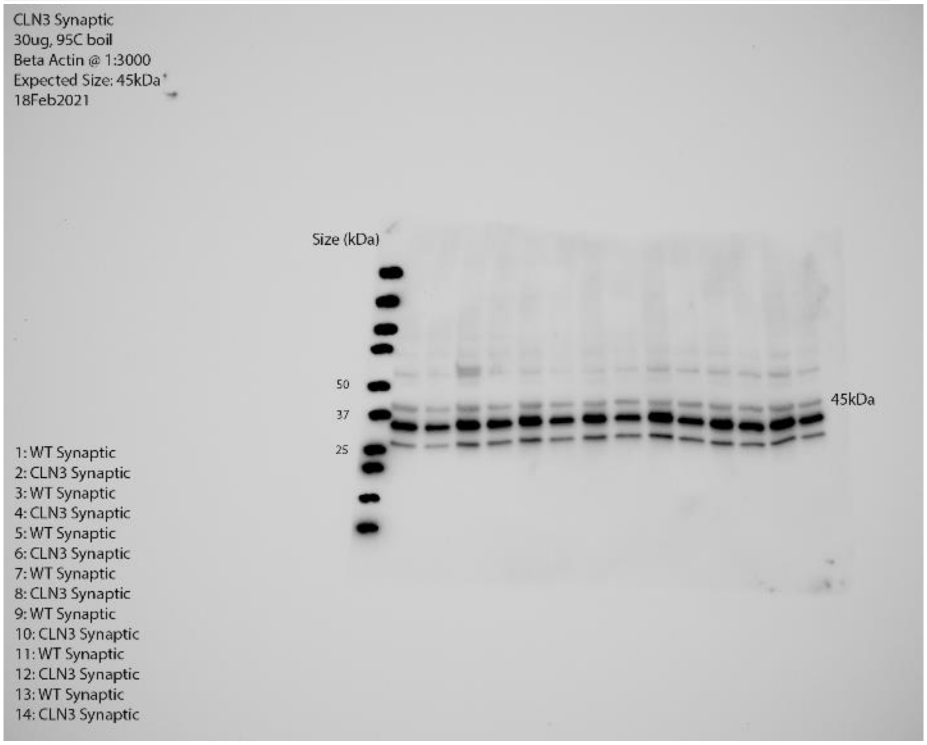
CLN3 Synaptic
30ug, 95C boil
Beta Actin @ 1:3000
Expected Size: 45kDa
18Feb2021
Size (kDa)
50
37
25
45kDa
1: WT Synaptic
2: CLN3 Synaptic
3: WT Synaptic
4: CLN3 Synaptic
5: WT Synaptic
6: CLN3 Synaptic
7: WT Synaptic
8: CLN3 Synaptic
9: WT Synaptic
10: CLN3 Synaptic
11: WT Synaptic
12: CLN3 Synaptic
13: WT Synaptic
14: CLN3 Synaptic

CLN3 Synaptic
30ug, 95C boil
anti-STX7 @ 1:2000
Expected Size; 36kDa
17Feb2021

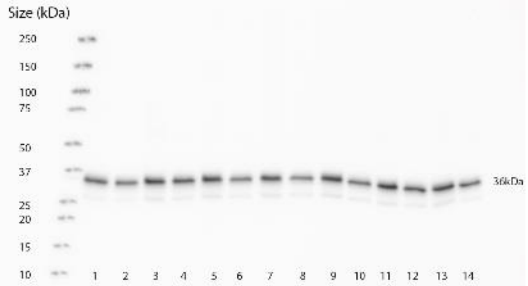

1: WT Synaptic
2: CLN3 Synaptic
3: WT Synaptic
4: CLN3 Synaptic
5: WT Synaptic
6: CLN3 Synaptic
7: WT Synaptic
8: CLN3 Synaptic
9: WT Synaptic
10: CLN3 Synaptic
11: WT Synaptic
12: CLN3 Synaptic
13: WT Synaptic
14: CLN3 Synaptic

CLN3 Synaptic
30ug load, 95C boil
VTI1B @ 1:1000
Expected Size: 27kDa
16Feb2021

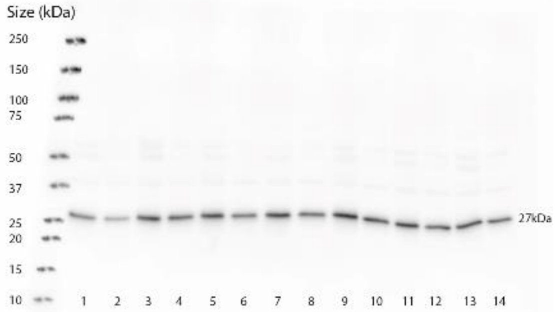

1: WT Synaptic
2: CLN3 Synaptic
3: WT Synaptic
4: CLN3 Synaptic
5: WT Synaptic
6: CLN3 Synaptic
7: WT Synaptic
8: CLN3 Synaptic
9: WT Synaptic
10: CLN3 Synaptic
11: WT Synaptic
12: CLN3 Synaptic
13: WT Synaptic
14: CLN3 Synaptic

CLN3 Cytosolic (blot 9)
30ug load, 95C boil
GAPDH @ 1:2000
Expected Size: 37kDa
02Jul2021

1: WT Cytosolic
2: CLN3 Cytosolic
3: WT Cytosolic
4: CLN3 Cytosolic
5: WT Cytosolic
6: CLN3 Cytosolic
7: WT Cytosolic
8: CLN3 Cytosolic
9: WT Cytosolic
10: CLN3 Cytosolic
11: WT Cytosolic
12: CLN3 Cytosolic
13: WT Cytosolic
14: CLN3 Cytosolic

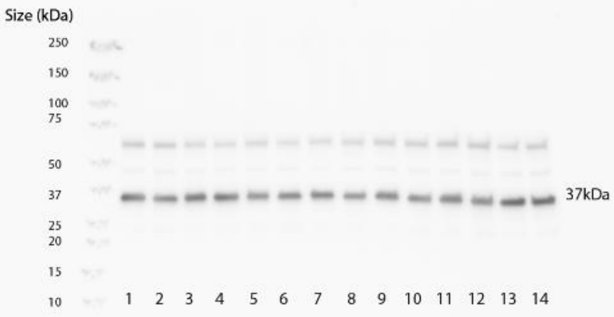

CLN3 Cytosolic (blot 9)
30ug load, 95C boil
MUNC18 @ 1:1000
Expected Size: 65kDa
01Jul2021

1: WT Cytosolic
2: CLN3 Cytosolic
3: WT Cytosolic
4: CLN3 Cytosolic
5: WT Cytosolic
6: CLN3 Cytosolic
7: WT Cytosolic
8: CLN3 Cytosolic
9: WT Cytosolic
10: CLN3 Cytosolic
11: WT Cytosolic
12: CLN3 Cytosolic
13: WT Cytosolic
14: CLN3 Cytosolic

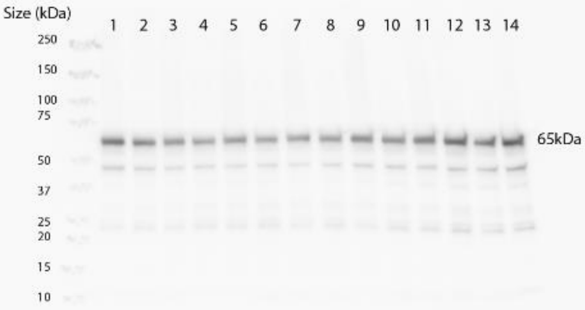

CLN3 Synaptic (blot 9)
30ug load, 95C boil
GAPDH @ 1:2000
Expected Size: 37kDa
02Jul2021

1: WT Synaptic
2: CLN3 Synaptic
3: WT Synaptic
4: CLN3 Synaptic
5: WT Synaptic
6: CLN3 Synaptic
7: WT Synaptic
8: CLN3 Synaptic
9: WT Synaptic
10: CLN3 Synaptic
11: WT Synaptic
12: CLN3 Synaptic
13: WT Synaptic
14: CLN3 Synaptic

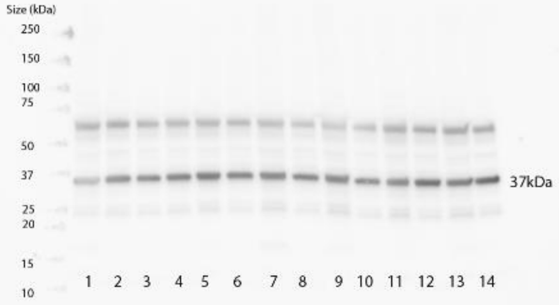

CLN3 Synaptic (blot 9)
30ug load, 95C boil
MUNC18 @ 1:1000
Expected Size: 65 kDa
01Jul2021

1: WT Synaptic
2: CLN3 Synaptic
3: WT Synaptic
4: CLN3 Synaptic
5: WT Synaptic
6: CLN3 Synaptic
7: WT Synaptic
8: CLN3 Synaptic
9: WT Synaptic
10: CLN3 Synaptic
11: WT Synaptic
12: CLN3 Synaptic
13: WT Synaptic
14: CLN3 Synaptic

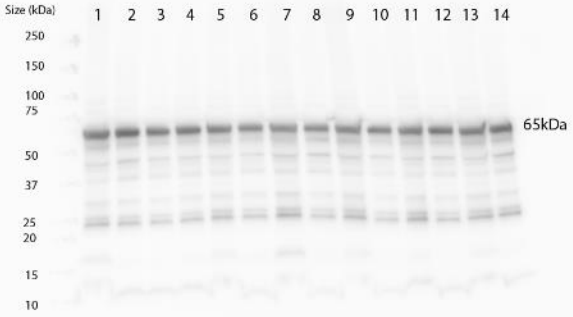

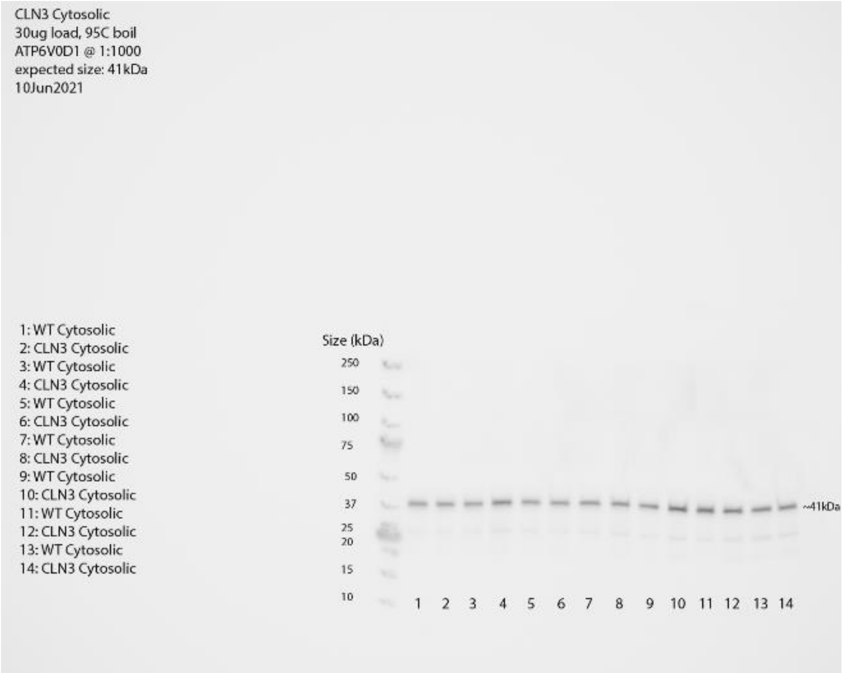
CLN3 Cytosolic
30ug load, 95C boil
ATP6V0D1 @ 1:1000
expected size: 41kDa
10Jun2021
1: WT Cytosolic
2: CLN3 Cytosolic
3: WT Cytosolic
4: CLN3 Cytosolic
5: WT Cytosolic
6: CLN3 Cytosolic
7: WT Cytosolic
8: CLN3 Cytosolic
9: WT Cytosolic
10: CLN3 Cytosolic
11: WT Cytosolic
12: CLN3 Cytosolic
13: WT Cytosolic
14: CLN3 Cytosolic
Size (kDa)
250
150
100
75
50
37
25
20
15
10
~41kDa
1 2 3 4 5 6 7 8 9 10 11 12 13 14

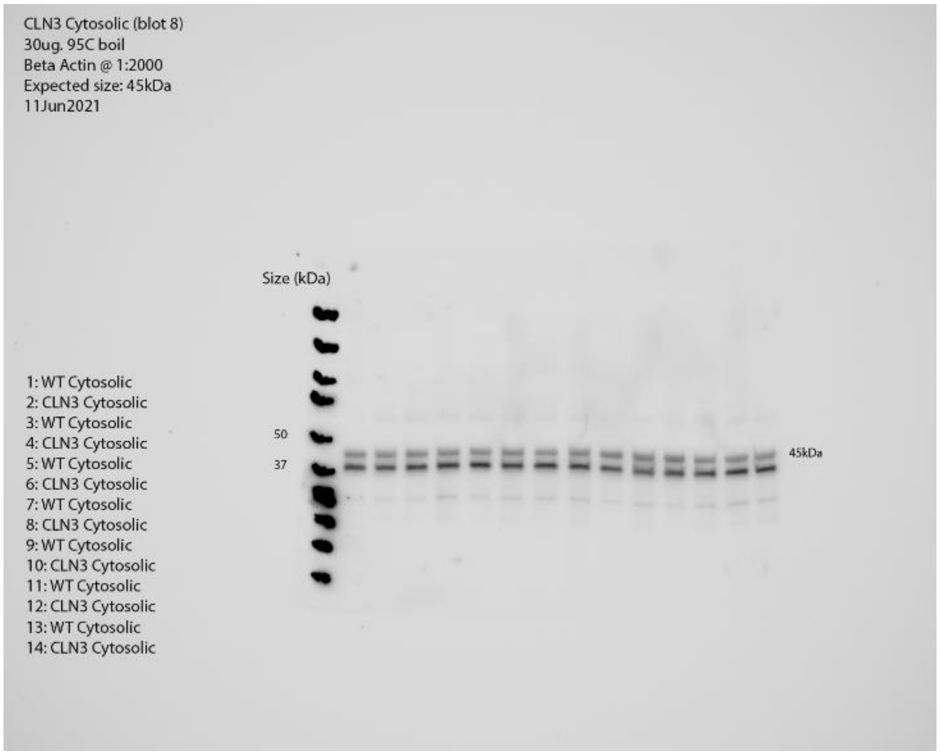
CLN3 Cytosolic (blot 8)
30ug. 95C boil
Beta Actin @ 1:2000
Expected size: 45kDa
11Jun2021
Size (kDa)
1: WT Cytosolic
2: CLN3 Cytosolic
3: WT Cytosolic
4: CLN3 Cytosolic
5: WT Cytosolic
6: CLN3 Cytosolic
7: WT Cytosolic
8: CLN3 Cytosolic
9: WT Cytosolic
10: CLN3 Cytosolic
11: WT Cytosolic
12: CLN3 Cytosolic
13: WT Cytosolic
14: CLN3 Cytosolic
50
37
45kDa

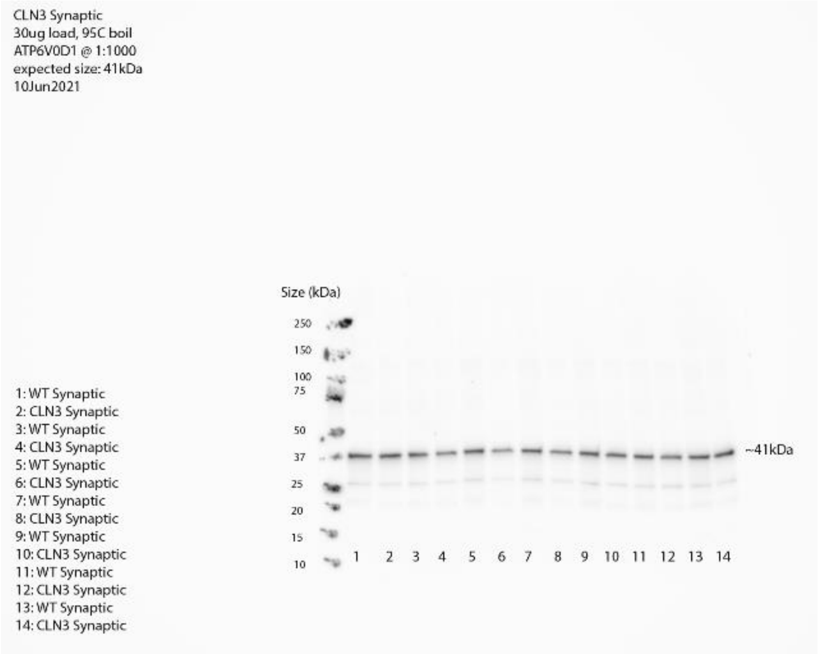
CLN3 Synaptic
30ug load, 95C boil
ATP6V0D1 @ 1:1000
expected size: 41kDa
10Jun2021
Size (kDa)
250
150
100
75
50
37
25
20
15
10
~41kDa
1 2 3 4 5 6 7 8 9 10 11 12 13 14
1: WT Synaptic
2: CLN3 Synaptic
3: WT Synaptic
4: CLN3 Synaptic
5: WT Synaptic
6: CLN3 Synaptic
7: WT Synaptic
8: CLN3 Synaptic
9: WT Synaptic
10: CLN3 Synaptic
11: WT Synaptic
12: CLN3 Synaptic
13: WT Synaptic
14: CLN3 Synaptic

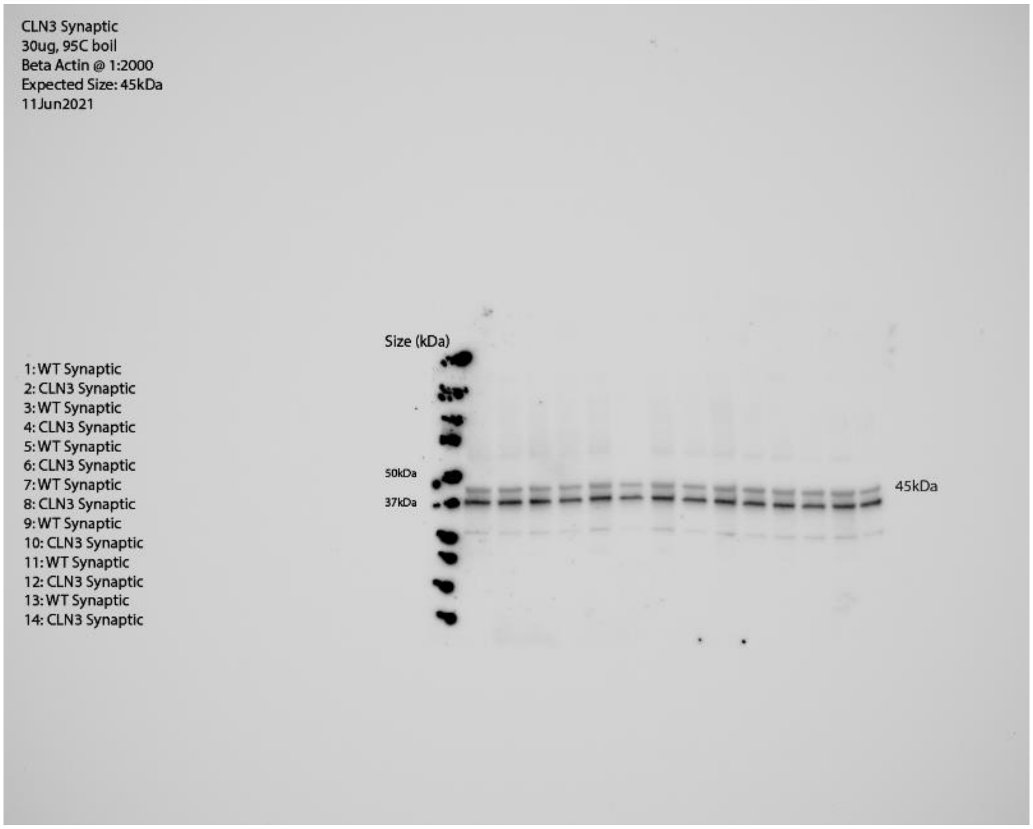
CLN3 Synaptic
30ug, 95C boil
Beta Actin @ 1:2000
Expected Size: 45kDa
11Jun2021
Size (kDa)
50kDa
37kDa
45kDa
1: WT Synaptic
2: CLN3 Synaptic
3: WT Synaptic
4: CLN3 Synaptic
5: WT Synaptic
6: CLN3 Synaptic
7: WT Synaptic
8: CLN3 Synaptic
9: WT Synaptic
10: CLN3 Synaptic
11: WT Synaptic
12: CLN3 Synaptic
13: WT Synaptic
14: CLN3 Synaptic

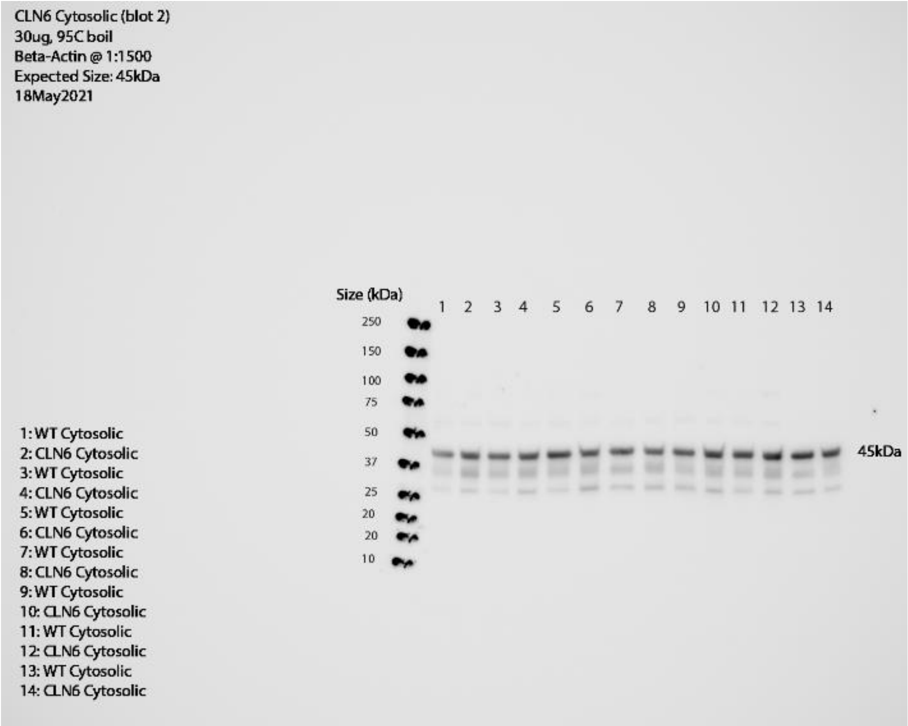
CLN6 Cytosolic (blot 2)
30ug, 95C boil
Beta-Actin @ 1:1500
Expected Size: 45kDa
18May2021
Size (kDa)
1 2 3 4 5 6 7 8 9 10 11 12 13 14
250
150
100
75
50
37
25
20
20
10
45kDa
1: WT Cytosolic
2: CLN6 Cytosolic
3: WT Cytosolic
4: CLN6 Cytosolic
5: WT Cytosolic
6: CLN6 Cytosolic
7: WT Cytosolic
8: CLN6 Cytosolic
9: WT Cytosolic
10: CLN6 Cytosolic
11: WT Cytosolic
12: CLN6 Cytosolic
13: WT Cytosolic
14: CLN6 Cytosolic

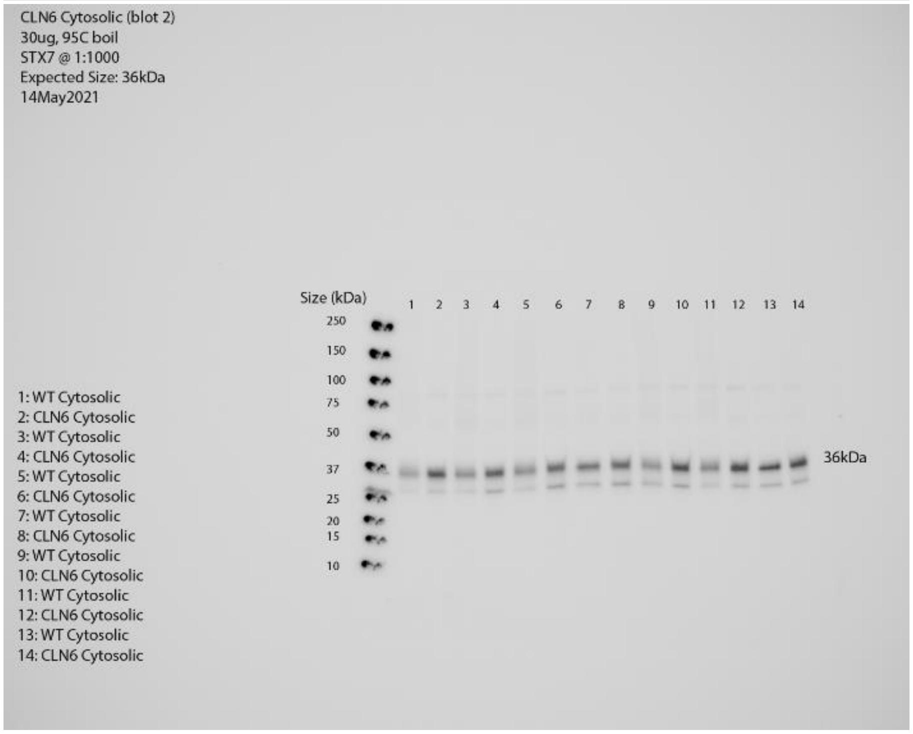
CLN6 Cytosolic (blot 2)
30ug, 95C boil
STX7 @ 1:1000
Expected Size: 36kDa
14May2021
Size (kDa)
1 2 3 4 5 6 7 8 9 10 11 12 13 14
250
150
100
75
50
37
25
20
15
10
36kDa
1: WT Cytosolic
2: CLN6 Cytosolic
3: WT Cytosolic
4: CLN6 Cytosolic
5: WT Cytosolic
6: CLN6 Cytosolic
7: WT Cytosolic
8: CLN6 Cytosolic
9: WT Cytosolic
10: CLN6 Cytosolic
11: WT Cytosolic
12: CLN6 Cytosolic
13: WT Cytosolic
14: CLN6 Cytosolic

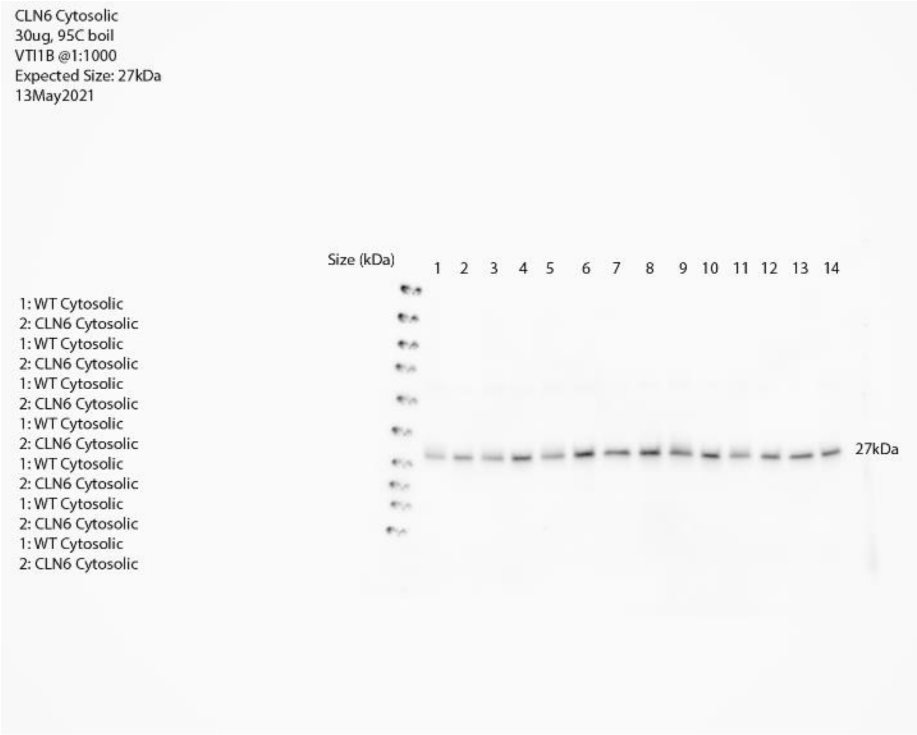
CLN6 Cytosolic
30ug, 95C boil
VTI1B @1:1000
Expected Size: 27kDa
13May2021
Size (kDa)
1 2 3 4 5 6 7 8 9 10 11 12 13 14
27kDa
1: WT Cytosolic
2: CLN6 Cytosolic
1: WT Cytosolic
2: CLN6 Cytosolic
1: WT Cytosolic
2: CLN6 Cytosolic
1: WT Cytosolic
2: CLN6 Cytosolic
1: WT Cytosolic
2: CLN6 Cytosolic
1: WT Cytosolic
2: CLN6 Cytosolic
1: WT Cytosolic
2: CLN6 Cytosolic

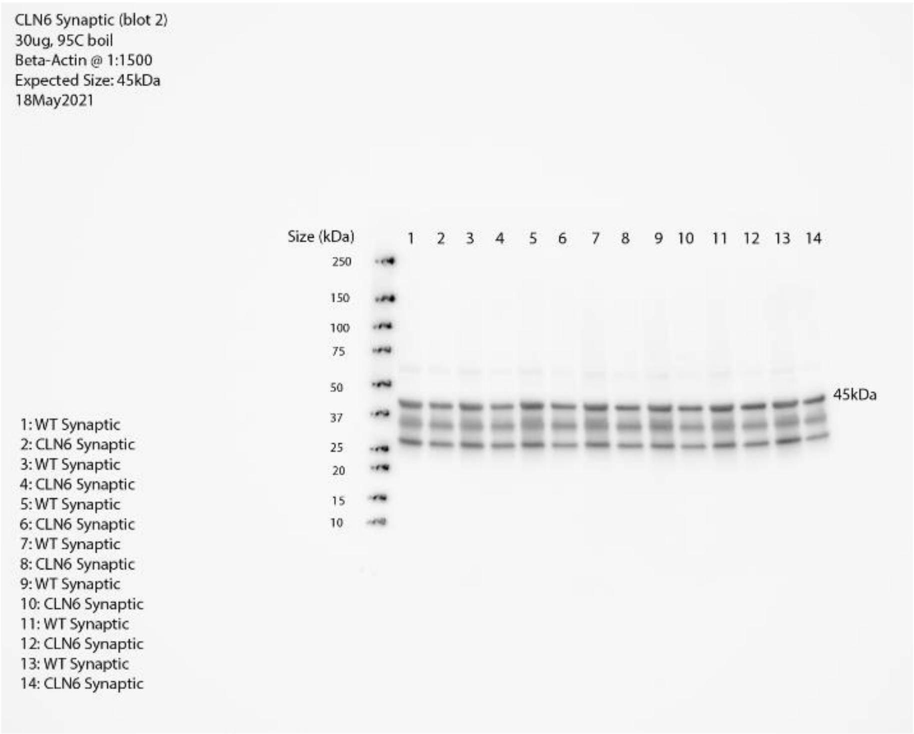
CLN6 Synaptic (blot 2)
30ug, 95C boil
Beta-Actin @ 1:1500
Expected Size: 45kDa
18May2021
Size (kDa)
1 2 3 4 5 6 7 8 9 10 11 12 13 14
250
150
100
75
50
37
25
20
15
10
45kDa
1: WT Synaptic
2: CLN6 Synaptic
3: WT Synaptic
4: CLN6 Synaptic
5: WT Synaptic
6: CLN6 Synaptic
7: WT Synaptic
8: CLN6 Synaptic
9: WT Synaptic
10: CLN6 Synaptic
11: WT Synaptic
12: CLN6 Synaptic
13: WT Synaptic
14: CLN6 Synaptic

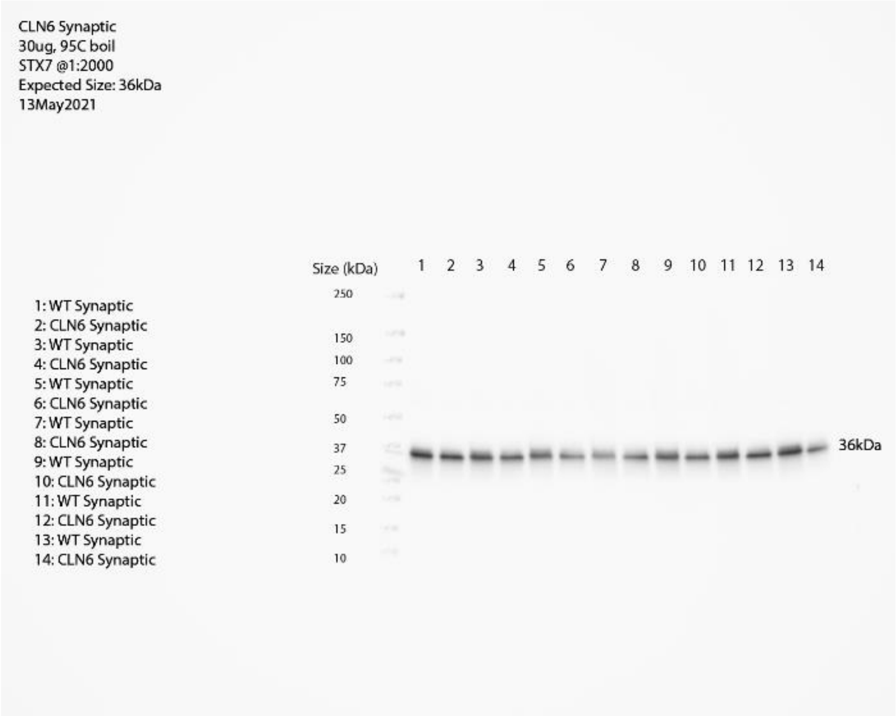
CLN6 Synaptic
30ug, 95C boil
STX7 @1:2000
Expected Size: 36kDa
13May2021
Size (kDa)
1 2 3 4 5 6 7 8 9 10 11 12 13 14
250
150
100
75
50
37
25
20
15
10
36kDa
1: WT Synaptic
2: CLN6 Synaptic
3: WT Synaptic
4: CLN6 Synaptic
5: WT Synaptic
6: CLN6 Synaptic
7: WT Synaptic
8: CLN6 Synaptic
9: WT Synaptic
10: CLN6 Synaptic
11: WT Synaptic
12: CLN6 Synaptic
13: WT Synaptic
14: CLN6 Synaptic

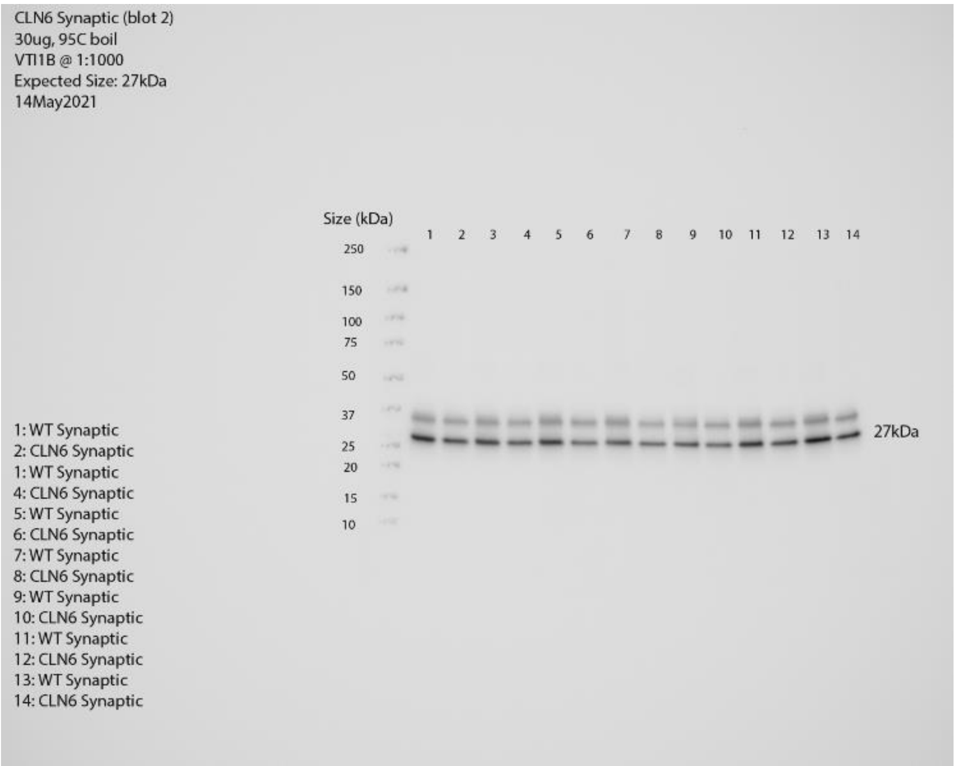
CLN6 Synaptic (blot 2)
30ug, 95C boil
VTI1B @ 1:1000
Expected Size: 27kDa
14May2021
Size (kDa)
1 2 3 4 5 6 7 8 9 10 11 12 13 14
250
150
100
75
50
37
25
20
15
10
27kDa
1: WT Synaptic
2: CLN6 Synaptic
1: WT Synaptic
4: CLN6 Synaptic
5: WT Synaptic
6: CLN6 Synaptic
7: WT Synaptic
8: CLN6 Synaptic
9: WT Synaptic
10: CLN6 Synaptic
11: WT Synaptic
12: CLN6 Synaptic
13: WT Synaptic
14: CLN6 Synaptic

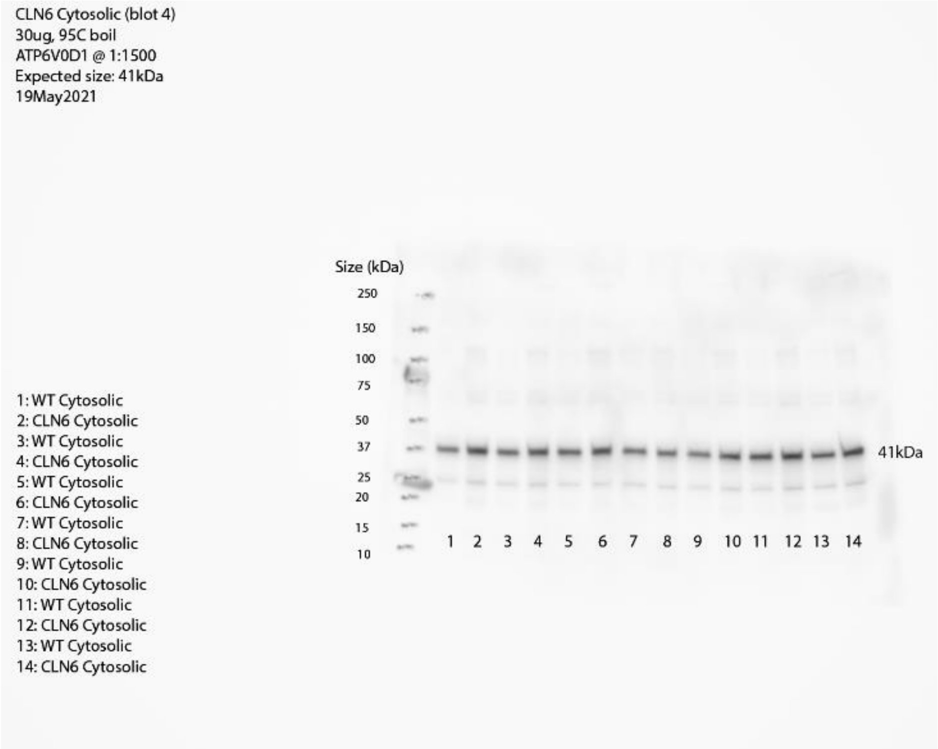
CLN6 Cytosolic (blot 4)
30ug, 95C boil
ATP6V0D1 @ 1:1500
Expected size: 41kDa
19May2021
1: WT Cytosolic
2: CLN6 Cytosolic
3: WT Cytosolic
4: CLN6 Cytosolic
5: WT Cytosolic
6: CLN6 Cytosolic
7: WT Cytosolic
8: CLN6 Cytosolic
9: WT Cytosolic
10: CLN6 Cytosolic
11: WT Cytosolic
12: CLN6 Cytosolic
13: WT Cytosolic
14: CLN6 Cytosolic
Size (kDa)
250
150
100
75
50
37
25
20
15
10
1 2 3 4 5 6 7 8 9 10 11 12 13 14
41kDa

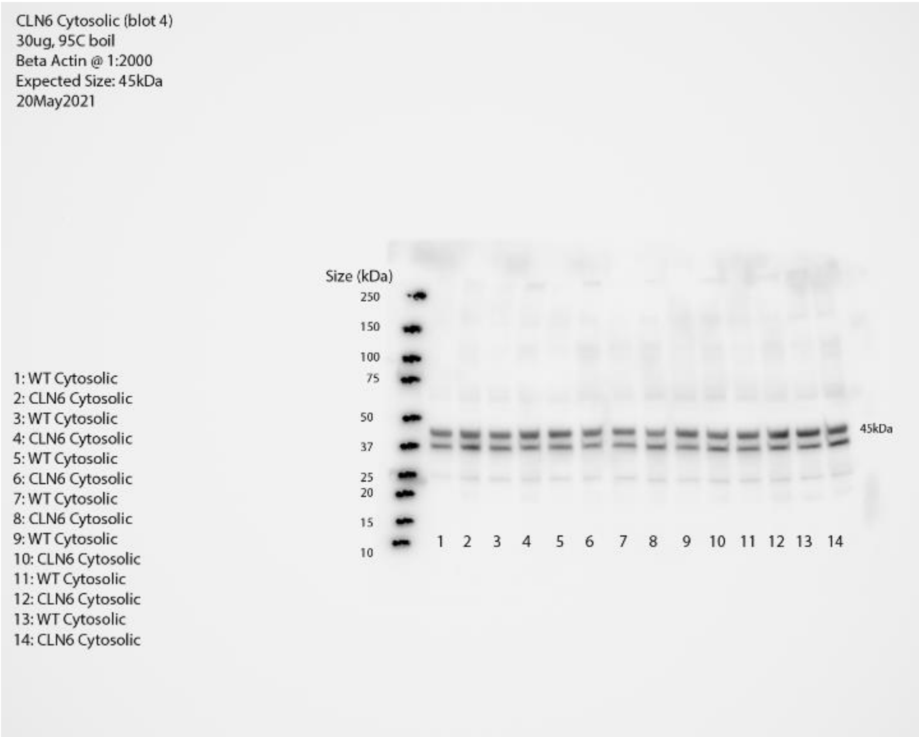
CLN6 Cytosolic (blot 4)
30ug, 95C boil
Beta Actin @ 1:2000
Expected Size: 45kDa
20May2021
1: WT Cytosolic
2: CLN6 Cytosolic
3: WT Cytosolic
4: CLN6 Cytosolic
5: WT Cytosolic
6: CLN6 Cytosolic
7: WT Cytosolic
8: CLN6 Cytosolic
9: WT Cytosolic
10: CLN6 Cytosolic
11: WT Cytosolic
12: CLN6 Cytosolic
13: WT Cytosolic
14: CLN6 Cytosolic
Size (kDa)
250
150
100
75
50
37
25
20
15
10
1 2 3 4 5 6 7 8 9 10 11 12 13 14
45kDa

CLN6 Synaptic (blot 4)
30ug, 95C boil
ATP6V0D1 @ 1:1500
Expected size: 41kDa
19May2021

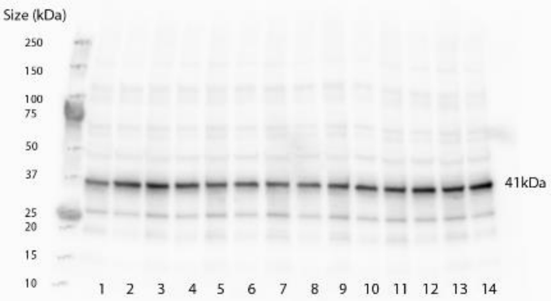

1: WT Synaptic
2: CLN6 Synaptic
3: WT Synaptic
4: CLN6 Synaptic
5: WT Synaptic
6: CLN6 Synaptic
7: WT Synaptic
8: CLN6 Synaptic
9: WT Synaptic
10: CLN6 Synaptic
11: WT Synaptic
12: CLN6 Synaptic
13: WT Synaptic
14: CLN6 Synaptic

CLN6 Synaptic (blot 4)
30ug, 95C boil
Beta Actin @ 1:2000
Expected Size: 45kDa
20May2021

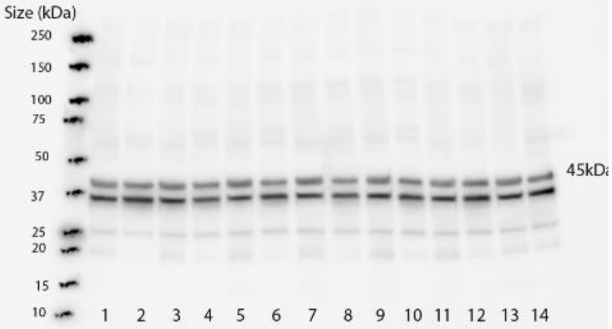

1: WT Synaptic
2: CLN6 Synaptic
3: WT Synaptic
4: CLN6 Synaptic
5: WT Synaptic
6: CLN6 Synaptic
7: WT Synaptic
8: CLN6 Synaptic
9: WT Synaptic
10: CLN6 Synaptic
11: WT Synaptic
12: CLN6 Synaptic
13: WT Synaptic
14: CLN6 Synaptic

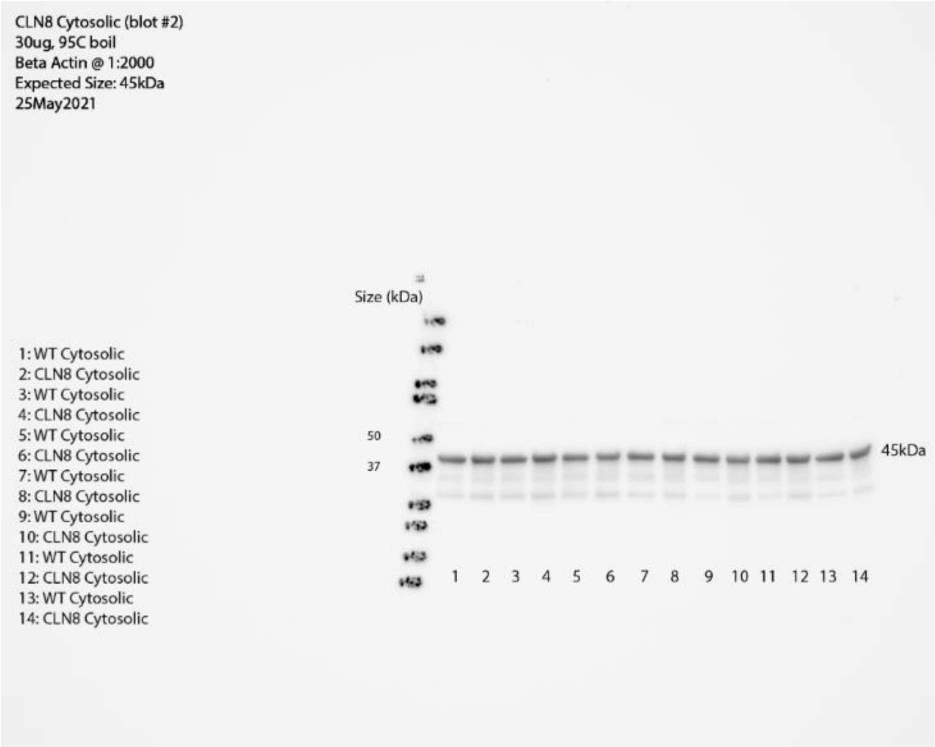
CLN8 Cytosolic (blot #2)
30ug, 95C boil
Beta Actin @ 1:2000
Expected Size: 45kDa
25May2021
Size (kDa)
50
37
45kDa
1 2 3 4 5 6 7 8 9 10 11 12 13 14
1: WT Cytosolic
2: CLN8 Cytosolic
3: WT Cytosolic
4: CLN8 Cytosolic
5: WT Cytosolic
6: CLN8 Cytosolic
7: WT Cytosolic
8: CLN8 Cytosolic
9: WT Cytosolic
10: CLN8 Cytosolic
11: WT Cytosolic
12: CLN8 Cytosolic
13: WT Cytosolic
14: CLN8 Cytosolic

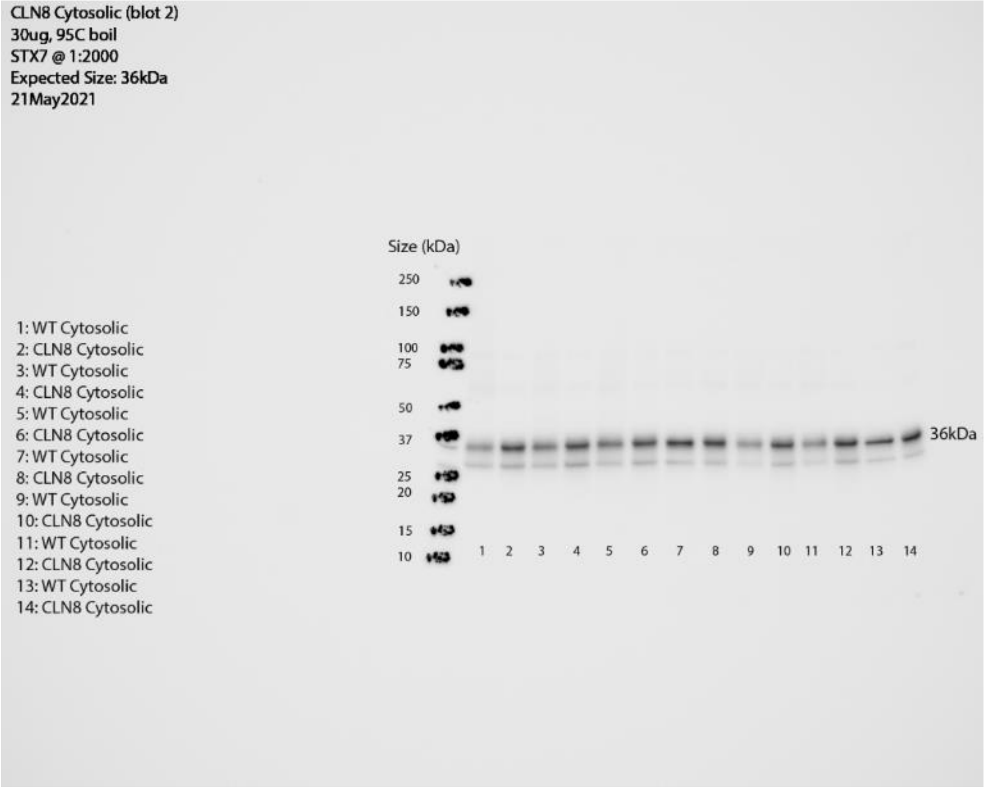
CLN8 Cytosolic (blot 2)
30ug, 95C boil
STX7 @ 1:2000
Expected Size: 36kDa
21May2021
Size (kDa)
250
150
100
75
50
37
25
20
15
10
36kDa
1 2 3 4 5 6 7 8 9 10 11 12 13 14
1: WT Cytosolic
2: CLN8 Cytosolic
3: WT Cytosolic
4: CLN8 Cytosolic
5: WT Cytosolic
6: CLN8 Cytosolic
7: WT Cytosolic
8: CLN8 Cytosolic
9: WT Cytosolic
10: CLN8 Cytosolic
11: WT Cytosolic
12: CLN8 Cytosolic
13: WT Cytosolic
14: CLN8 Cytosolic

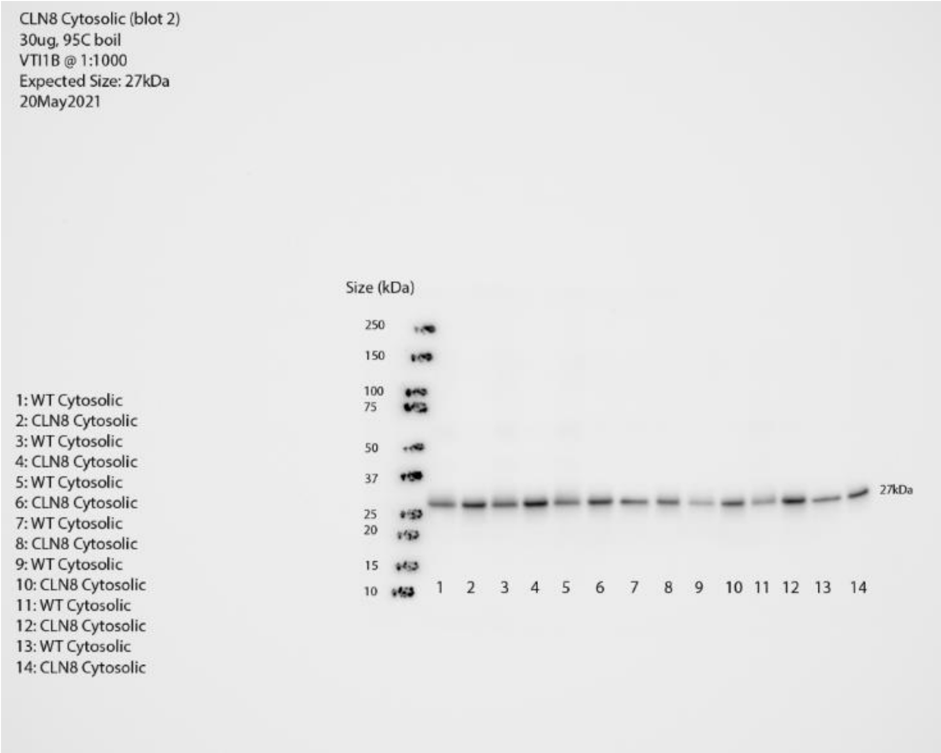
CLN8 Cytosolic (blot 2)
30ug, 95C boil
VTI1B @ 1:1000
Expected Size: 27kDa
20May2021
Size (kDa)
250
150
100
75
50
37
25
20
15
10
1 2 3 4 5 6 7 8 9 10 11 12 13 14
27kDa
1: WT Cytosolic
2: CLN8 Cytosolic
3: WT Cytosolic
4: CLN8 Cytosolic
5: WT Cytosolic
6: CLN8 Cytosolic
7: WT Cytosolic
8: CLN8 Cytosolic
9: WT Cytosolic
10: CLN8 Cytosolic
11: WT Cytosolic
12: CLN8 Cytosolic
13: WT Cytosolic
14: CLN8 Cytosolic

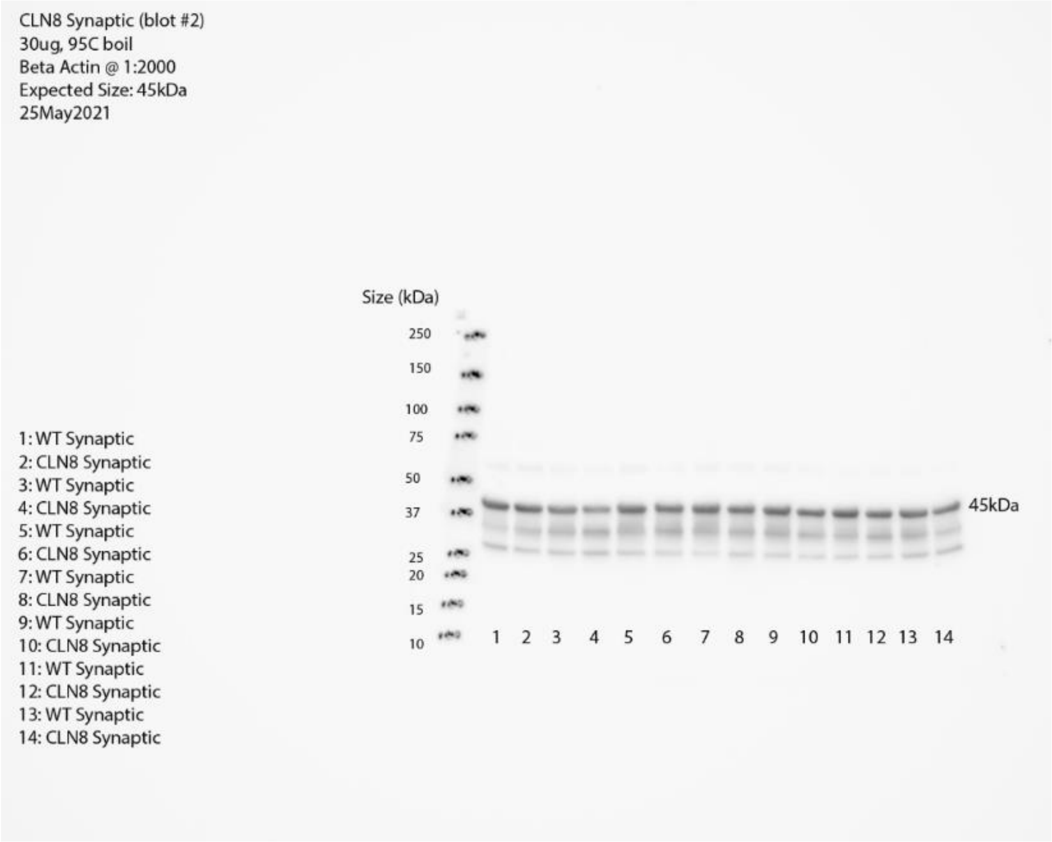
CLN8 Synaptic (blot #2)
30ug, 95C boil
Beta Actin @ 1:2000
Expected Size: 45kDa
25May2021
Size (kDa)
250
150
100
75
50
37
25
20
15
10
1 2 3 4 5 6 7 8 9 10 11 12 13 14
45kDa
1: WT Synaptic
2: CLN8 Synaptic
3: WT Synaptic
4: CLN8 Synaptic
5: WT Synaptic
6: CLN8 Synaptic
7: WT Synaptic
8: CLN8 Synaptic
9: WT Synaptic
10: CLN8 Synaptic
11: WT Synaptic
12: CLN8 Synaptic
13: WT Synaptic
14: CLN8 Synaptic

CLN8 Synaptic (blot 2)
30ug, 95C boil
STX7 @ 1:2000
Expected Size: 36kDa
20May2021

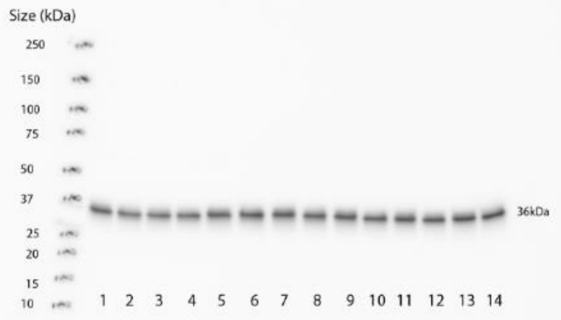

1: WT Synaptic
2: CLN8 Synaptic
3: WT Synaptic
4: CLN8 Synaptic
5: WT Synaptic
6: CLN8 Synaptic
7: WT Synaptic
8: CLN8 Synaptic
9: WT Synaptic
10: CLN8 Synaptic
11: WT Synaptic
12: CLN8 Synaptic
13: WT Synaptic
14: CLN8 Synaptic

CLN8 Synaptic (blot 2)
30ug, 95C boil
VTI1B @ 1:1000
Expected Size: 27kDa
21May2021

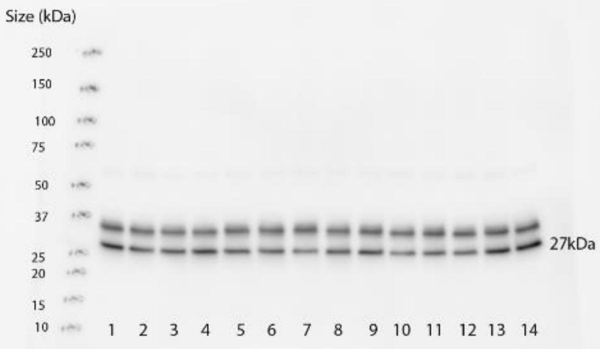

1: WT Synaptic
2: CLN8 Synaptic
3: WT Synaptic
4: CLN8 Synaptic
5: WT Synaptic
6: CLN8 Synaptic
7: WT Synaptic
8: CLN8 Synaptic
9: WT Synaptic
10: CLN8 Synaptic
11: WT Synaptic
12: CLN8 Synaptic
13: WT Synaptic
14: CLN8 Synaptic
