# Supplementary material for: Transmembrane Batten Disease Proteins Interact With a Shared Network of Vesicle Sorting Proteins, Impacting Their Synaptic Enrichment
Source: Front Neurosci. 2022 May 25;16:834780. doi: 10.3389/fnins.2022.834780 (PMC9174988; doi:10.3389/fnins.2022.834780)

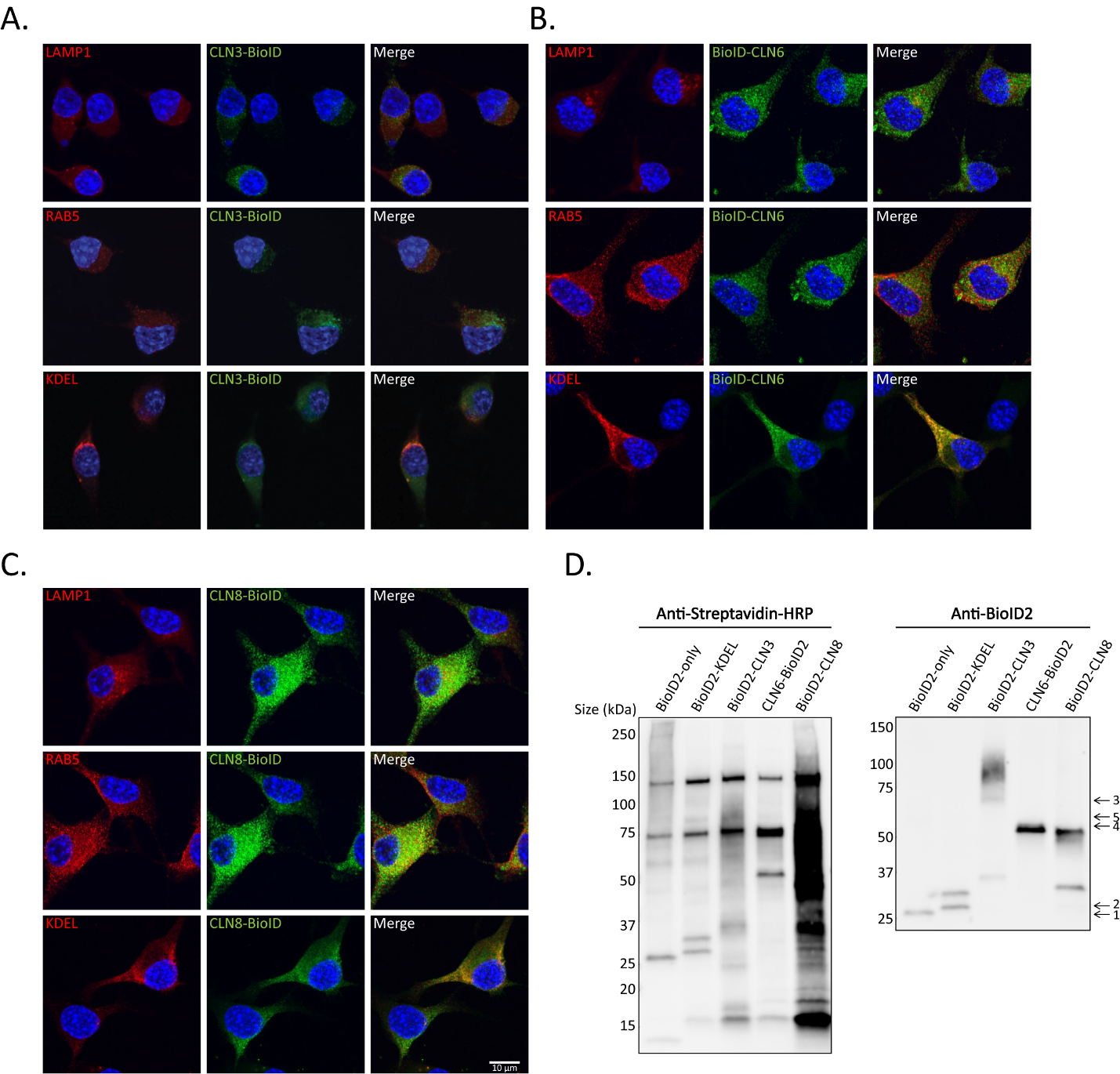


**Supplemental Figure 1: BioID2 expression and protein biotinylation confirmed by IF and western blot**

(A) CLN3-BioID, (B) BioID-CLN6, and (C)CLN8-BioID constructs colocalize with markers for lysosomes (anti-LAMP1), endosomes (anti-RAB5) and endoplasmic reticulum (anti-KDEL). Expected band sizes denoted as follows: BioID-only (1) - 26kDa, BioID2-KDEL (2) - 28kDa, BioID2-CLN3 (3) - 68kDa, CLN6-BioID2 (4) - 52kDa, BioID2-CLN8 (5) - 59kDa. Scale bar indicates 10 microns and applies to all images. (D) Western blot analysis of BioID lysates demonstrates successful expression of BioID constructs and resulting biotinylation of proximal proteins.


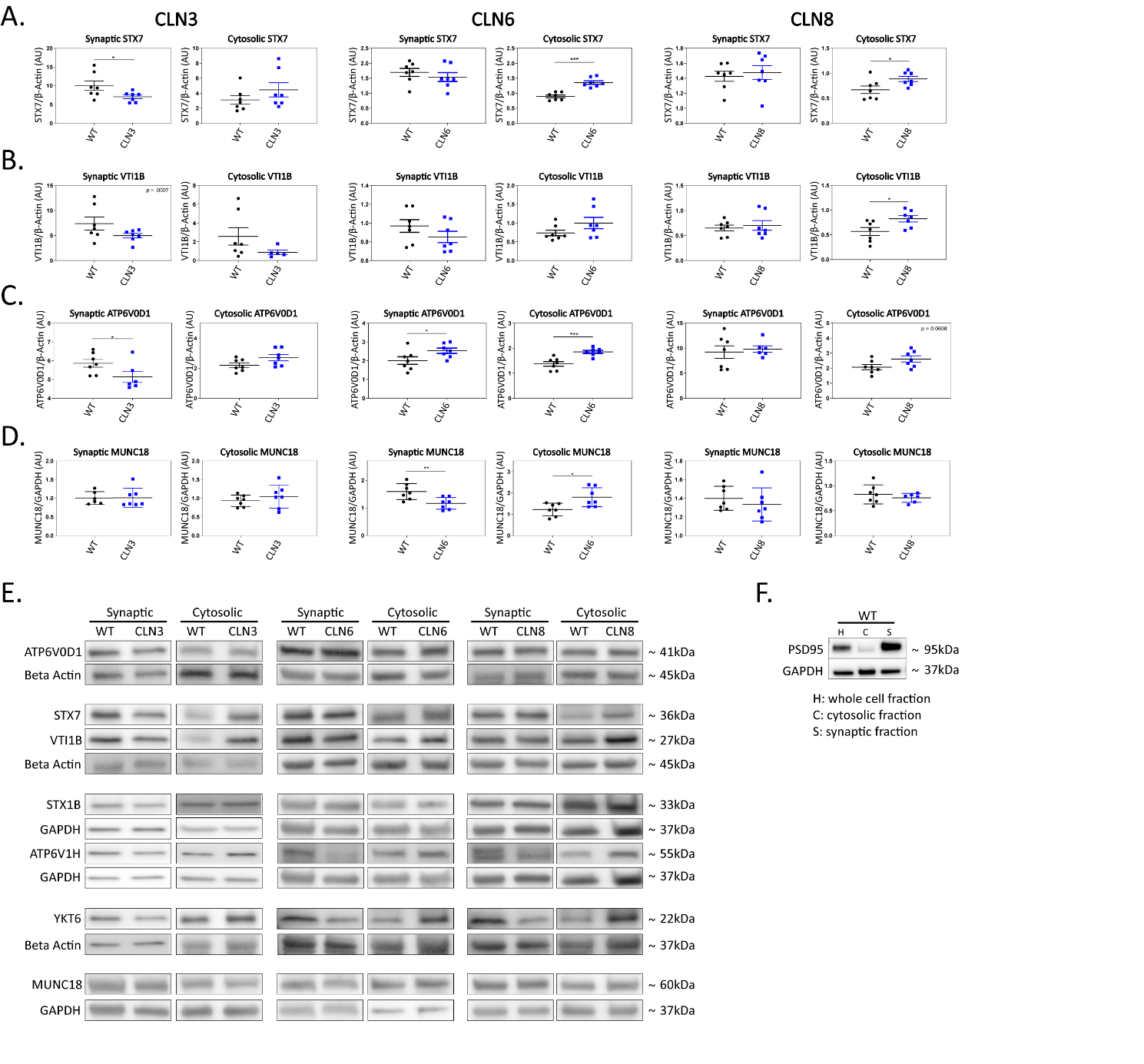


**Supplemental Figure 2: Western blot analysis of P30 mouse cortex brain fractions reveals significant changes in target protein levels when compared to WT.**

(A) Analysis of target interactor STX7 shows synaptic depletion in CLN3, and cytosolic accumulation in both CLN6 and CLN8. (B) Also demonstrated was cytosolic accumulation of VTI1B in CLN8, (C) synaptic depletion of APT6V0D1 in CLN3 and CLN6, and cytosolic accumulation of ATP6V0D1 in CLN6 mutants. (D) CLN3 and CLN8 mutants show no changes in MUNC18 levels, however significant synaptic depletion and cytosolic accumulation was noted in CLN6 mutants. (E) Western blot representative images show visually apparent changes in mutant synaptic and cytosolic fractions compared to WT. (F) Brain fractionation was validated by probing samples for synaptic marker PSD95 and GAPDH to control for protein loading, confirming successful separation of synaptic and cytosolic compartments. Outliers identified by ROUT analyses, Q=5%. One-tailed t-test (synaptic), two-tailed t-test (cytosolic), *p<0.05, **p<0.01, ***p<0.001, n=7 mice, mean +/- SEM.

**Supplementary Table 1: Protein-protein interactions validated by coimmunoprecipitation**

Shared BioID-identified interactors of CLN3, CLN6 and CLN8 validated by coimmunoprecipitation. “Yes” denotes stable interaction confirmed while “-“ denotes interactions that were not stable under the conditions used for coimmunoprecipitations.


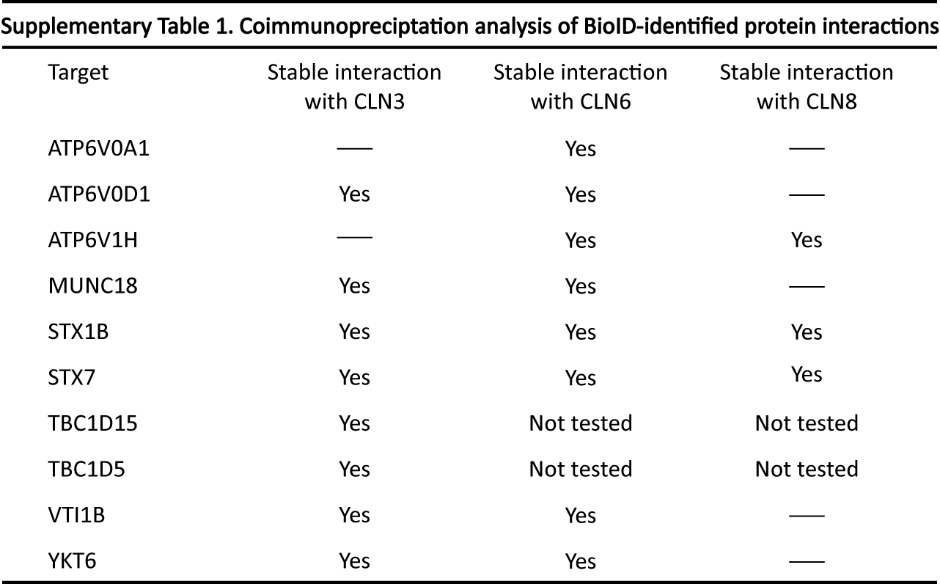


**Supplementary Table 2: Synaptic depletion and cytosolic accumulation of targets was observed in P30 mouse cortex brain fractions.**

Outliers identified by ROUT analyses, Q=5%. One-tailed t-test (synaptic), two-tailed t-test (cytosolic), *p<0.05, **p<0.01, ***p<0.001, n=7, mean +/- SEM


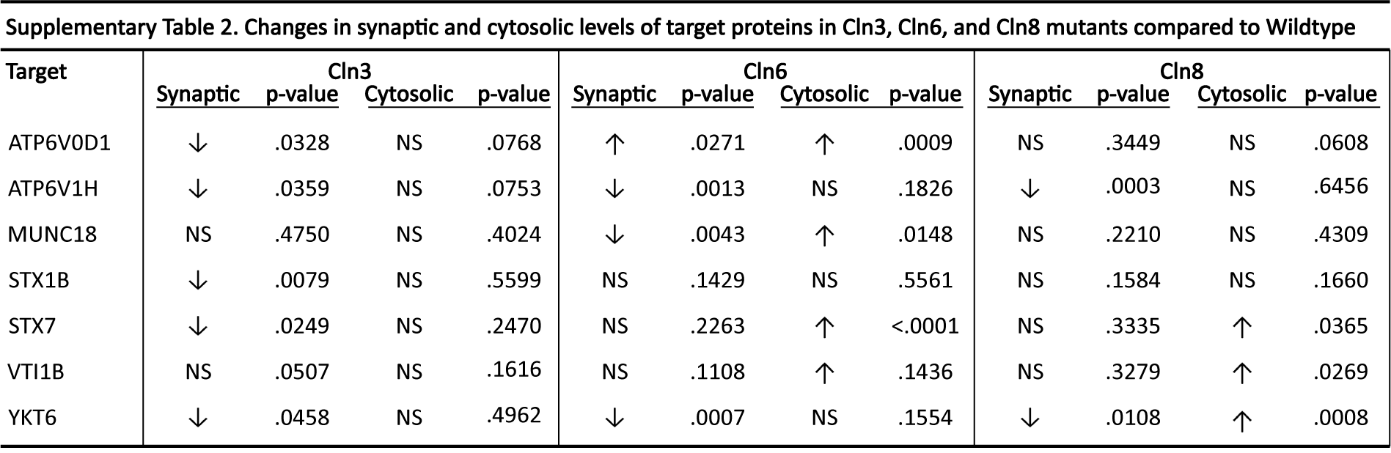

Supplement: Supplementary file 3 [file Data_Sheet_2.docx]
