# Supplementary material for: Transmembrane Batten Disease Proteins Interact With a Shared Network of Vesicle Sorting Proteins, Impacting Their Synaptic Enrichment
Source: Front Neurosci. 2022 May 25;16:834780. doi: 10.3389/fnins.2022.834780 (PMC9174988; doi:10.3389/fnins.2022.834780)
Supplement: Supplementary file 4 [file Data_Sheet_3.docx]

**Online Methods**

**Generation of stable expression BioID2 cell lines:**

Cell lines were maintained in DMEM with 10% fetal bovine serum at 37°C with 5% CO_2_. Phoenix cells (ATCC #CRL-3213) were transfected with retroviral BioID2^1^ only pBabe, BioID2-KDEL pBabe (translated within the secretory pathway by means of a human albumin signal sequence), BioID2-hCLN3 pRetroX, mCln6-BioID2 pRetroX, or BioID2-hCLN8 pRetroX using the lipofectamine 3000 lipofection kit (ThermoFisher #L3000008). Viral supernatant was transferred to Neuro2a (ATCC #CCL-131) cells with polybrene (4μg/ml) for 72h transduction prior to selection in maintenance media with puromycin (40 μg/ml) for selection of properly expressing cells.

**Preparation of cell lines and biotinylated samples:**

BioID protocol was followed as previously described with minor modifications^2^. Briefly, six 10 cm plates were seeded with Neuro2a cells stably expressing BioID2 only, BioID2-KDEL, BioID2-CLN3, Cln6-BioID2, or BioID-CLN8. Control cells received 50 μM biotin supplementation for 18h prior to lysis and CLN3/6/8 cells were cultured with doxycycline (20 μg/ml) for 18h prior to supplementation with 50 μM biotin for an additional 18h. Cell lysates were pre-cleared with gelatin sepharose 4B (Cytvia, #17095601) prior to overnight pulldown with streptavidin (Cytvia, #90100484). After several washes, biotinylated proteins bound to streptavidin beads were shipped to Sanford-Burnham-Prebys Medical Institute for MS analysis.

**Post-pulldown SDS-Page and Immunoblot:**

Post-pulldown samples were analyzed via western blot by resuspending 1% of streptavidin beads in SDS-PAGE sample buffer and boiling for 5 minutes. Proteins were separated on a 4-20% gradient gel (BioRad #4561096) and transferred to nitrocellulose membrane (Fisher Scientific #PI88018). To detect biotinylated proteins, blots were probed with Streptavidin-HRP diluted in 0.4% Triton X-100/phosphate-buffered saline and imaged via enhanced chemiluminescent (ECL) substrate detection on LiCor Odyssey FC. Following the detection of biotinylated proteins, HRP activity was quenched via 20-minute incubation in 30% H_2_O_2_. Blots were then blocked with 10% adult bovine serum and 0.2% Triton X-100 in PBS for 30 minutes, and then probed with chicken anti-BioID2. An HRP-conjugated anti-chicken antibody was used to repeat ECL detection.

**Protein Digestion:**

Biotinylated proteins were digested directly on-beads. Briefly, beads were thawed, and cysteine disulfide bonds were reduced with 10 mM tris (2-carboxyethyl) phosphine (TCEP) at 30°C for 60 minutes followed by cysteine alkylation with 30 mM iodoacetamide (IAA) in the dark at room temperature for 30 min. Following alkylation, urea was diluted to 1 M urea using 50 mM ammonium bicarbonate, and proteins were finally subjected to overnight digestion with mass spec grade Trypsin/Lys-C mix (Promega #PRV5071). Finally, beads were pulled down, and the solution with peptides collected into a new tube. The beads were then washed once with 50mM ammonium bicarbonate to increase peptide recovery. Following digestion, samples were acidified with formic acid (FA) and subsequently desalted using AssayMap C18 cartridges mounted on an Agilent AssayMap BRAVO liquid handling system, C18 cartridges were first conditioned with 100% acetonitrile (ACN), followed 0.1% FA. Sample was then loaded onto the conditioned C18 cartridge, washed with 0.1% FA, and eluted with 60% ACN, 0.1% FA. Finally, the organic solvent was removed in a SpeedVac concentrator prior to LC-MS/MS analysis.

**LC-MS**

Dried samples were reconstituted with 2% acetonitrile, 0.1% formic acid and analyzed by LC-MS/MS using a Proxeon EASY nanoLC system (Thermo Fisher Scientific) coupled to a Q-Exactive Plus mass spectrometer (Thermo Fisher Scientific). Peptides were separated using an analytical C18 Acclaim PepMap column 75µm x 500 mm, 2µm particles (Thermo Scientific) using a 121-min gradient (6 to 23% in 72 min followed by 23 to 34% in 45 min) of solvent B (80% acetonitrile, 0.1% formic acid) at a flow rate of 300nL/min. The mass spectrometer was operated in positive data-dependent acquisition mode. MS1 spectra were measured with a resolution of 70,000, an AGC target of 1e6 and a mass range from 350 to 1700 m/z. Up to 12 MS2 spectra per duty cycle were triggered, fragmented by HCD, and acquired with a resolution of 17,500 and an AGC target of 5e4, an isolation window of 1.6 m/z and a normalized collision energy of 25. Dynamic exclusion was enabled with duration of 25 sec. (see details in annex section).

**Data Analysis**

All mass spectra were analyzed with MaxQuant software version 1.5.5.1. MS/MS spectra were searched against the Mus musculus Uniprot protein sequence database (downloaded in January 2018) and GPM cRAP sequences (commonly known protein contaminants). Precursor mass tolerance was set to 20ppm and 4.5ppm for the first search where initial mass recalibration was completed and for the main search, respectively. Product ions were searched with a mass tolerance 0.5 Da. The maximum precursor ion charge state used for searching was 7. Carbamidomethylation of cysteine was searched as a fixed modification, while oxidation of methionine and acetylation of protein N-terminal were searched as variable modifications. Enzyme was set to trypsin in a specific mode and a maximum of two missed cleavages was allowed for searching. The target-decoy-based false discovery rate (FDR) filter for spectrum and protein identification was set to 1%.

**Definition of candidate interactors**

Candidate interactors for CLN3, CLN6, and CLN8 by the respective BioID2 data set were defined as proteins showing at least 3-fold greater enrichment by label free quantification in CLN-BioID2 samples vs. BioID2 (CLN3) or BioID2-KDEL (Cln6 and CLN8) only samples, in accordance with previous applications of this method^3^.

**Functional analysis of significant interactors:**

GO analysis and pathway analysis of significant interactors was performed using the DAVID functional annotation tool (https://david.ncifcrf.gov/summary.jsp). Candidate interactors were converted to official gene symbol then selected as the identifier in this tool. Mus musculus was selected as the species of origin. Threshold for the number of genes per term was set to 4 and EASE threshold set to 0.1.

GO network visualization was performed using Cytoscape version 3.8.2 with ClueGO version 2.5.7 and CluePedia version 1.5.7 applications. Significant interactors were input using symbol ID. Organism was set to *Mus musculus*, with grouping based on functional group and fusion of parent and child terms based upon gene abundance. Redundant groups with >50% similar gene identity were merged. Significance defined as p < 0.05 with Bonferroni post hoc correction.

**Animals:**

All animals used for this study were maintained in an AAALAC accredited facility in accordance with IACUC approval (Sanford Research, Sioux Falls, SD). Wildtype (WT), CLN3 (*Cln3^Δex7/8^*), CLN6 *(Cln6^nclf^)*, and CLN8 (*Cln8^mnd^*) on C57BL/6J backgrounds were used for *in vivo* experiments. For all *in vivo* experiments, individual animals were used as the representative replicates.

**Tissue collection:**

Mice were sacrificed by carbon dioxide inhalation at a flow rate of 3L/min, diaphragm puncture and cardiac perfusion performed with ice-cold 1X Phosphate Buffered Saline (PBS) (Corning #21-040-CV). Brains were harvested and cortices collected on dry ice for protein lysates or frozen fresh for RNAscope. For synaptic and cytosolic preparations, the cortices were collected on wet ice.

**RNAscope**:

*Cln3*, *Cln6*, *Cln8* (ACDBio #497591, 546671, and 1029351 respectively) transcript were visualized using ACD RNAscope Multiplex Fluorescent V2 Assay per manufacturers protocol and counterstained with DAPI. Brains were sectioned coronally at 16μm with post fixation in 10% Formalin and serial dehydration according to manufacturer’s protocol and mounted with Dako Faramount. Images were captured at 40x on a Nikon Ni-E microscope

**Immunocytochemistry (ICC)**:

Cells were cultured as outlined above on glass coverslips prior to fixation using 2% PFA/2% Sucrose in 0.1M PBS for 30 minutes. Coverslips were then permeabilized and blocked using blocking solution (0.3% Triton X-100, 1% Bovine Serum Albumin, and 5% goat serum in 0.1M PBS) for 30 minutes followed by incubation with primary antibodies diluted in blocking solution for one hour at room temperature. Coverslips were washed with 0.1M PBS and incubated with secondary antibodies diluted in blocking solution for 30 minutes at room temperature prior to mounting with Dako Faramount media ^3^. Cells were imaged using a 60X oil-immersion objective on a Nikon A1 inverted confocal.

**Plasmid transfection:**

Minipreps of cloned plasmids were prepared per manufacturer’s protocol (Zymo Research #D4210). Resulting DNA was transfected into neuroblastoma cell cultures using Lipofectamine transfection following provided protocol (Fisher #L3000015). PCAG-FLAG-CLN3 and pCAG-HA-CLN8 expression plasmids were co-transfected with pCAG-GFP (Addgene #11150) to confirm efficient transfection. Cells were transfected for 24-48 hours, washed 3 times with 1X PBS, and collected in cOmplete-Lysis M lysis buffer supplemented with protease and phosphatase inhibitors.

**AAV transduction:**

scAAV9.CB.CLN6 (AAV9-CLN6) was administered by intracerebroventricular injection into postnatal day1 *Cln6^nclf^* animals as previously described.^4^

**Protein Lysis (IP/Western Blotting):**

Transfected Neuro2A cells (CLN3/CLN8) and AAV9-CLN6 transduced cortical tissue (CLN6) were homogenized with a pestle homogenizer in 500μL cOmplete Lysis-M lysis buffer (Roche #04719956001) containing protease inhibitors (Roche #04693124001) and phosphatase inhibitors (Sigma #P5726 & P0044). Samples were sonicated on ice for 45 seconds per sample at 30% output (Branson #EDP 100-14-239)

**Brain Fractionation:**

Cortex samples from post-natal day 30 WT, *Cln3^Δex7/8^*, *Cln6^nclf^*, and *Cln8^mnd^* mice were weighed and homogenized in 10mL/gram Syn-PER buffer (Thermo #87793) containing protease and phosphatase inhibitors. Brain fractions were separated by density separation following the manufacturer’s protocol.

**Protein Quantification:**

Total protein concentration of samples were determined in triplicate using the Pierce BCA Protein Assay Kit (Pierce #23225) following manufacturer’s protocol.

**SDS Page and Immunoblotting:**

Equal amounts of lysed protein were diluted in water to a total volume of 37.5μL with 1X Laemmli Buffer (BioRad #1610747) supplemented with 10% β-Mercaptoethanol and incubated at 95°C for 10 minutes. Samples were loaded on 4-20% Mini-Protean TGX (BioRad #4561096) precast gels and ran at 225V for 30 minutes on ice. Immobilized proteins were transferred to nitrocellulose membranes in 20% Methanol Towbin transfer buffer for 60 minutes at 100V. Membranes were washed three times for five minutes in 1X TBS with 0.1% Tween-20 and blocked for 2 hours in TBST supplemented with 5% Adult Bovine Serum. Blots were incubated in primary antibody diluted in 5% ABS blocking buffer overnight at 4°C, washed three times, and incubated at 4°C for 2 hours in secondary antibody diluted in 5% blocking buffer. Blots were washed an additional three times, exposed to chemiluminescent substrate, and imaged with the ChemiDoc MP imaging system (BioRad #17001402). Re-probing of membranes was accomplished by incubating the membrane in Restore PLUS Western Blot Stripping Buffer (Thermo #46430) for 10 minutes at room temperature. These membranes were then washed five times for five minutes in TBST prior to a one-hour blocking period in 5% ABS blocking buffer. Antibodies used for immunoblotting are described below.

**Immunoprecipitation (CLN3/CLN6):**

Due to the lack of a commercially available antibody, FLAG-CLN3-expressing Neuro2A cells were used for immunoprecipitation experiments. 200μg FLAG-CLN3 protein lysate was diluted in a total volume of 500uL with cOmplete-Lysis M lysis buffer (Roche #04719956001) supplemented with protease and phosphatase inhibitors. Protein was incubated with anti-FLAG antibody at 1:50 dilution overnight at 4C. The following day, samples were added to 25μL pre-washed Pierce A/G magnetic beads (Pierce #88802) and incubated at room temperature for 75 minutes with mixing. Beads were collected using a magnetic stand and flow-through was removed. Beads were washed twice in 1X PBS with 0.05% Tween-20, followed by purified water. Target antigen was eluted at room temperature in 1X Laemmli buffer supplemented with 10% β-Mercaptoethanol for 15 minutes with agitation and analyzed by immunoblotting. CLN6 immunoprecipitation experiments were carried out as described above with AAV9-hCLN6-expressing *Cln6^nclf^* brain lysate. Samples were incubated with target antibody, and immobilized proteins were probed with anti-hCLN6.

**Immunoprecipitation (CLN8):**

Neuro2A cell lysate transfected with an HA-tagged CLN8 expression plasmid was used for CLN8 immunoprecipitation experiments. 500uL cOmplete-Lysis M containing 150μg total protein was added to 25μL pre-washed Pierce Anti-HA Magnetic beads (Pierce #88836). Beads were incubated at room temperature for 30 minutes, collected with a magnetic stand, washed, and eluted in 1X Laemmli buffer supplemented with 10% β-Mercaptoethanol. Eluate was analyzed by western blotting protocol described above.

**Immunoprecipitation (SNARE pulldowns):**

200μg total protein from P30 WT, *Cln3^Δex7/8^*, *Cln6^nclf^*, and *Cln8^mnd^* cortical mouse brain lysate was diluted in a total volume of 500μL with cOmplete-Lysis M lysis buffer (Roche #04719956001) supplemented with protease and phosphatase inhibitors. Samples were incubated with anti-SNAP25 antibody at 1:50 dilution overnight at 4°C. The following morning, samples were added to 25μL pre-washed Pierce A/G magnetic beads (Pierce #88802) and incubated at room temperature for 75 minutes with agitation. Beads were collected with a magnetic stand, washed, and eluted in 1X Laemmli buffer supplemented with 10% β-Mercaptoethanol for 15 minutes. Eluate was analyzed by western blotting protocol described above.

**Antibodies:**

Antibodies used included anti-ATP6V0A1 (Abnova #H00000535-A01), anti-ATP6V0D1 (Proteintech #18274-1-AP), anti-ATP6V1H (Invitrogen #PA5-22134), anti-Beta Actin (Cell Signaling #4967), anti-BioID2 (BioFront #BID2-CP-100), anti-hCLN6 (proprietary), anti-FLAG (Sigma Aldrich #F1804), anti-GAPDH (Cell Signaling #5174), anti-KDEL (Novus #NBP1-97469), anti-LAMP1 (Abcam #ab24871), anti-MUNC18 (Invitrogen #PA1-742), anti-PSD95 (Cell Signaling #3409), anti-RAB5 (Cell Signaling #3547), anti-SNAP25 (Abcam #ab5666), anti-Streptavidin (Abcam #ab7403), anti-STX1B (R&D Systems #MAB6848), anti-STX7 (Invitrogen #PA5-81945), anti-Synaptobrevin2 (Synaptic Systems #104 211), anti-Syntaxin1 (Synaptic Systems #110 011), anti-TBC1D15 (Proteintech #17252-1-AP), anti-TBC1D5 (Proteintech #17078-1-AP), anti-VTI1B (Abcam #184170), anti-YKT6 (Abcam #ab236583), anti-Chicken HRP (Abcam #ab97135) anti-Mouse HRP (Cell Signaling #7076S), anti-Rabbit HRP (Invitrogen #31462), anti-Rabbit 568 (Invitrogen #A11011), anti-Mouse 568 (Invitrogen #A21134), anti-Chicken 647 (Invitrogen #A21449).

**Cloning:**

PCAG-FLAG-CLN3 and pCAG-HA-CLN8 expression vectors were generated using restriction enzyme-based cloning.

**Statistical Analyses:**

Statistical analyses were performed using GraphPad Prism (v9.1.2; San Diego, CA). Details of the specific tests are noted in the figure legends. In general, one-tailed and two-tailed unpaired t-tests were employed without correcting for multiple comparisons. *p<0.05, **p<0.01, ***p<0.001. Outliers were removed with the ROUT method, Q=5%.

References

1 Kim, D. I. *et al.* An improved smaller biotin ligase for BioID proximity labeling. *Molecular biology of the cell* **27**, 1188-1196, doi:10.1091/mbc.E15-12-0844 (2016).

2 May, D. G. & Roux, K. J. BioID: A Method to Generate a History of Protein Associations. *Methods in molecular biology (Clifton, N.J.)* **2008**, 83-95, doi:10.1007/978-1-4939-9537-0_7 (2019).

3 Brudvig, J. J. *et al.* MARCKS Is Necessary for Netrin-DCC Signaling and Corpus Callosum Formation. *Molecular neurobiology* **55**, 8388-8402, doi:10.1007/s12035-018-0990-3 (2018).

4 Cain, J. T. *et al.* Gene Therapy Corrects Brain and Behavioral Pathologies in CLN6-Batten Disease. *Molecular therapy : the journal of the American Society of Gene Therapy* **27**, 1836-1847, doi:10.1016/j.ymthe.2019.06.015 (2019).
